# Communication around HPV vaccination for adolescents in low- and middle-income countries: a systematic scoping overview of systematic reviews

**DOI:** 10.1186/s13643-019-1100-y

**Published:** 2019-08-01

**Authors:** Hakan Safaralilo Foss, Ann Oldervoll, Atle Fretheim, Claire Glenton, Simon Lewin

**Affiliations:** 1 Faculty of Medicine, University of Oslo, Oslo, Norway; 2 Division of Health Services, Norwegian Institute of Public Health, Oslo, Norway; 3 Health Systems Research Unit, South African Medical Research Council, Cape Town, South Africa

**Keywords:** HPV, Human papillomavirus, Vaccine, Communication, Intervention, Adolescents, Low-income countries, Middle-income countries, Low- and middle-income countries, Systematic scoping overview of systematic reviews

## Abstract

**Background:**

Human papillomavirus (HPV) infection causes cervical cancer. More than 80% of those diagnosed with cervical cancer live in low- and middle-income countries (LMICs). The World Health Organization recommends vaccination as a public health measure against cervical cancer. Communication interventions are able to change how people think about vaccination and are thus instrumental in addressing vaccine hesitancy. Our aim was to provide a broad scoping overview of the available evidence on communication with adolescents, parents, and other stakeholders around HPV vaccination for adolescents, with a specific focus on LMICs.

**Methods:**

We conducted a systematic scoping overview of systematic reviews addressing a range of questions regarding communication around HPV vaccination. We considered reviews published between 2007 and 2018 focusing on communication around HPV vaccination and that searched for qualitative or quantitative studies for inclusion. We searched the Epistemonikos database which includes reviews from multiple electronic databases. Two overview authors screened titles and abstracts and examined potentially eligible reviews in full text. Data extraction was performed by one overview author and verified by a second. We assessed the reliability of the included reviews using an adapted version of AMSTAR 2.

**Results:**

We included twelve reviews in our overview. Four reviews assessed the effectiveness of communication interventions. These interventions intended to inform or educate about HPV and HPV vaccination, such as videos and fact sheets, or to remind or recall, such as text message reminders. Eight reviews assessed factors associated with HPV vaccination uptake, including communication-related factors such as whether the vaccine was recommended by a physician and people’s knowledge regarding the vaccine. Nine reviews searched for studies from LMICs, but most found only a small number of studies from these countries.

**Conclusions:**

The small number of studies identified from LMICs is of concern as these countries face the largest burden of disease related to HPV. This scoping overview also found and excluded a number of reviews because of important methodological limitations, highlighting the need for future reviews to use appropriate methods. The overview indicates areas in which further primary studies are needed on HPV vaccination communication in LMICs.

**Systematic review registration:**

Open Science Framework https://osf.io/agzb4/

**Electronic supplementary material:**

The online version of this article (10.1186/s13643-019-1100-y) contains supplementary material, which is available to authorized users.

## Background

Cervical cancer is the fourth most frequent cancer among women worldwide. Around 530,000 women are diagnosed with cervical cancer and around 265,000 women die from the disease every year [1]. Human papillomavirus (HPV) infection causes cervical cancer and there is growing evidence of HPV being a relevant factor in other anogenital and head and neck cancers. More than 80% of those diagnosed with cervical cancer live in low- and middle-income countries (LMICs) [1] where it is one of the leading causes of death by cancer. In Africa, cervical cancer is the primary cause of cancer death [1].

The HPV vaccine has one of the highest estimated per-person impacts on mortality of all vaccines [2] and is anticipated to prevent death in over 15 per 1000 persons vaccinated [3]. The World Health Organization (WHO) recommends the vaccination of 9–13-year-old girls as the most cost-effective public health measure against cervical cancer. The WHO also recommends investing in an HPV vaccine communication strategy at a country level that reflects the vaccine’s unique characteristics, including the newness of the vaccine, the fact that it is targeted at adolescents and pre-adolescents, and that it protects against a sexually transmitted disease [2].

Communication interventions can impact how people think and feel about vaccination and can be used to address aspects or factors contributing to vaccine hesitancy [2, 4]. It is therefore important to understand communication needs and gaps in relation to HPV vaccination and how these gaps are being addressed. It is also important to understand stakeholders’ views of these strategies; including those of adolescents, parents, other caregivers, and communities. Understanding these communication needs and gaps is especially important where the burden of cervical cancer and the need for implementation of HPV vaccination are highest. This systematic scoping overview of reviews, commissioned to inform discussion at a meeting of stakeholders in the WHO Africa Region, therefore, focuses on LMICs as defined by the World Bank [5].

Our findings can be used to prioritize areas where new or updated systematic reviews are needed on communication around HPV vaccination for adolescents, especially in LMICs. The findings can also be used to quickly identify reviews in this field. However, scoping overviews are not intended to synthesize results from the included reviews and this type of synthesis is therefore not presented in this overview. To our knowledge, no overviews of reviews have been conducted that summarize the available evidence on this topic.

## Aim

Our aim was to undertake a systematic scoping overview of systematic reviews of the available evidence on communication with adolescents, parents, and other stakeholders around HPV vaccination for adolescents.

Our specific objectives were to do the following:

- Identify systematic reviews on communication around HPV vaccination for adolescents
- Briefly describe and summarize the scope of each review and the evidence identified, considering specifically the relevance of these reviews for LMICs
- Identify areas in which new or updated systematic reviews are needed on communication around HPV vaccination for adolescents

As this was a scoping overview of reviews, we did not aim to synthesize the findings of the included systematic reviews.

## Methods

This systematic scoping overview of systematic reviews used methods adapted from those used for scoping reviews of individual studies [6, 7]. Like scoping reviews, this scoping overview “aim[s] to map *rapidly* the key concepts underpinning a research area and the main sources and types of evidence available” [8]. Its methods are reported according to the PRISMA Extension for Scoping Reviews, and the PRISMA-ScR checklist is included as Additional file 1.

### Criteria for considering reviews for this overview

#### Types of reviews

We included systematic reviews that focused on communication around HPV vaccination for male and female adolescents and addressed at least one of the following topic areas:

- Reviews of quantitative and/or qualitative studies of HPV vaccination communication issues or problems identified by any of the stakeholders (see below). This could include issues such as people’s HPV information needs and how they would like to receive that information
- Reviews of descriptive studies of the types of HPV vaccination communication interventions or strategies being used in different settings
- Reviews of qualitative and/or quantitative studies of stakeholders’ views of different HPV vaccination communication interventions or strategies, as well as factors affecting the implementation of these interventions
- Reviews of quantitative studies of the effectiveness of HPV vaccination communication interventions or strategies
- Reviews of quantitative studies of the costs or cost-effectiveness of HPV vaccination communication interventions or strategies

Exclusion criteria are the following:

- Reviews published before 2007 as we aimed to only include reviews published after the implementation of HPV vaccination programs [1]
- Reviews published in languages other than English, Norwegian, Swedish or Danish as we did not have the capacity within the team to extract data from these reviews. We kept a list of those reviews that appeared eligible but were not published in these languages
- Reviews that did not have a “Methods” section with explicit selection criteria for the inclusion of primary studies, or that had other important methodological limitations, as assessed using the approach described below

We did not exclude reviews that labeled themselves as scoping or rapid reviews.

#### Types of participants

Participants included one or both of the following groups:

- Adolescents (defined as aged 10–26 years for the purposes of this review). Where the ages of participants were not disaggregated, we included reviews where there was explicit mention that at least 70% of participants were between the ages of 10–26 years
- Other stakeholders such as parents, caregivers, families, communities, health care providers, and health service managers and policy-makers involved in HPV vaccination for adolescents

#### Types of outcomes and other types of information

We included reviews of the effectiveness and cost-effectiveness of communication strategies that measured any of the following outcomes:

- Knowledge of HPV, the HPV vaccine, and HPV vaccine services
- Attitudes towards HPV, the HPV vaccine, and HPV vaccine services
- HPV vaccination status
- Participants’ involvement in decision-making regarding HPV vaccination
- Participants’ confidence in the decision made regarding HPV vaccination
- Participants’ satisfaction with the health care provider
- Unintended effects linked to HPV vaccination interventions
- Health care provider outcomes (such as satisfaction with their involvement in the HPV vaccination communication program)
- Social outcomes such as school enrollment

We included other types of reviews that reported on any of the following types of information:

- Different types of HPV vaccination communication problems or interventions
- Participants’ attitudes towards and views regarding HPV vaccination (the term “attitudes” covers beliefs about vaccination, and may include intention to vaccinate) and HPV vaccination communication interventions or strategies
- Factors affecting the implementation of HPV vaccination communication interventions or strategies

The outcomes and other types of information were selected by three of the overview authors (HSF, AO, and SL) as the most relevant outcomes and other types of information in relation to providing an overview of the topic. As this was a systematic scoping overview of systematic reviews, we wanted to be as broad as possible regarding outcomes and other types of information.

### Search methods for identification of studies

We searched for relevant systematic reviews in the Epistemonikos database of systematic reviews (https://www.epistemonikos.org/), published between 2007 and 2018 (database searched 31 May 2018). The following databases are searched to populate the Epistemonikos database, with no language or publication status restrictions: Cochrane Database of Systematic Reviews (CDSR), PubMed, Embase, CINAHL (The Cumulative Index to Nursing and Allied Health Literature), PsycINFO, LILACS (Literatura Latinoamericana y del Caribe en Ciencias de la Salud), Database of Abstracts of Reviews of Effects (DARE), The Campbell Collaboration online library, JBI Database of Systematic Reviews and Implementation, and EPPI-Centre Evidence Library. The full search strategy is provided in Additional file 2.

### Data collection and analysis

#### Selection of reviews

Two overview authors independently screened titles and abstracts to identify potentially eligible reviews. We conducted a pilot screening of 20 full-text reviews to ensure agreement on our interpretation of the inclusion and exclusion criteria. Two overview authors examined potentially eligible reviews in full text to make a final decision on inclusion. Discrepancies were resolved either by a third overview author deciding on inclusion or through discussion between the two overview authors. Additional file 3 provides a complete list of reviews assessed in full text with reasons for exclusion. Table 1 provides a complete list of reviews excluded after data extraction for having important methodological limitations, using the approach described below.

**Table 1:** Table of reviews excluded from data synthesis due to important methodological limitations

Review	Methodological limitations (criterion number^1^)	Geographic settings of the included studies
Allen et al., 2010 [9]	Twelve minor limitations (2, 3, 4, 5, 7, 9, 10, 12, 13, 14, 15, 16)	U.S. (41 studies), Australia (5), Belgium (3), Brazil (2), Canada (*n* = 4), Columbia (*n* = 1), Finland (*n* = 1), Germany (*n* = 1): Hong Kong (*n* = 2), Iceland (*n* = 1), Mexico (*n* = 2), Netherlands (*n* = 1), Sweden (*n* = 1), Turkey (*n* = 1), UK (*n* = 10), and Vietnam (*n* = 1). Not specified: 2
Brewer et al., 2007 [10]	One major limitation (9). Ten minor limitations (2, 3, 4, 6, 7, 10, 12, 13, 14, 15).	USA
Catalan-Matamoros et al., 2017 [11]	One major limitation (9). Nine minor limitations (4, 5, 6, 7, 10, 12, 13, 15, 16).	Print media: US (*n* = 27), UK (*n* = 5), Canada (*n* = 8), Australia (*n* = 3), Israel (*n* = 1), Panama (*n* = 1), India (*n* = 1), China (*n* = 1), and Iran (*n* = 1). Television: US (*n* = 9). Radio: Australia (*n* = 1). Combination of media: US (*n* = 4) and Italy (*n* = 1)
Chan et al., 2012 [12]	One major limitation (9). Eleven minor limitations (2, 4, 5, 6, 7, 10, 12, 13, 14, 15).	Studies were conducted in Asia (*n* = 11), North America (*n* = 15), UK (*n* = 2), Europe (*n* = 6), and Australia (*n* = 2)
Crocker-Buque et al., 2017 [13]	One major limitation (9). Seven minor limitations (3, 6, 7, 10, 12, 13, 15).	US (*n* = 31), UK (*n* = 5), Canada (*n* = 3) and Australia (*n* = 2)
Cunningham et al., 2014 [14]	One major limitation (9). Nine minor limitations (2, 4, 5, 6, 10, 12, 13, 14, 15).	SSA: Botswana (1), South Africa (2), Nigeria (2), Kenya (3), Ghana (1), Uganda (1), Mali (1), Zambia (1), Tanzania (1) and Malawi (1)
Das et al., 2016 [15]	One major limitation (9). Nine minor limitations (2, 3, 4, 7, 10, 11, 12, 14, 15).	HICs.
Francis et al., 2017 [16]	One major limitation (9). Nine minor limitations (2, 4, 6, 7, 10, 12, 13, 15, 16).	USA
Galbraith et al., 2016 [17]	One major limitation (9). Eight minor limitations (2, 4, 5, 6, 7, 10, 13, 14).	N/R^2^
Gilkey et al., 2016 [18]	One major limitation (9). Eight minor limitations (2, 4, 5, 6, 7, 10, 12, 14).	N/R^2^
Holman et al., 2014 [19]	Two major limitations (4, 9). Nine minor limitations (1, 2, 5, 7, 10, 11, 12, 13, 14).	N/R^2^
Hyde et al., 2012 [20]	One major limitation (9). Eleven minor limitations (2, 4, 5, 6, 7, 8, 10, 12, 13, 14, 16).	97 (75%) were from high-income countries, 21 (16%) were from middle-income countries, and 4 (3%) were from low-income countries.
Kabakama et al., 2016 [21]	One major limitation (9). Nine minor limitations (2, 4, 5, 6, 7, 8, 10, 12, 13).	37 low- and middle-income countries
Karafillakis et al., 2017 [22]	One major limitation (9). Eleven minor limitations (2, 3, 4, 5, 6, 7, 8, 10, 12, 13, 14).	The majority of articles included were from the UK (35.2%), the Netherlands (11.7%), France (11.7%), Germany (8.3%), Greece (7.6%), and Sweden (6.2%).
Kessels et al., 2012 [23]	Eight minor limitations (2, 3, 4, 5, 6, 7, 10, 16).	Mostly USA
Loke et al., 2017 [24]	Two major limitations (9, 13). Seven minor limitations (2, 3, 4, 5, 7, 10, 15).	Information only provided for 28 studies. Geographic settings such as countries or cities: 17, secondary schools: 4, unspecified schools: 4, health center or community clinic: 3, colleges: 1. One study had both random digit dialing as their study setting for mothers and schools for adolescents as their setting.
Mishra, 2011 [25]	Two major limitations (9, 13). Eight minor limitations (2, 4, 5, 6, 7, 10, 12, 14, 16).	N/R^2^
Niccolai et al., 2015 [26]	One major limitation (9). Nine minor limitations (2, 3, 4, 5, 7, 10, 12, 13, 14).	USA
Paul et al., 2014 [27]	Two major limitations (4, 9). Eight minor limitations (2, 5, 6, 7, 10, 12, 13, 16).	Seventeen countries from Africa, Asia, Australia, Europe, Latin America, and North America are represented
Perlman et al., 2014 [28]	One major limitation (9). Nine minor limitations (2, 4, 5, 6, 7, 10, 12, 13, 14).	Cameroon: 5. Nigeria: 5. South Africa: 4. Tanzania: 4. Uganda: 3. Kenya: 2. Botswana: 1. Ghana: 1. Lesotho: 1. Mali: 1. Rwanda: 1. Zambia: 1. Zimbabwe: 1
Rosen et al., 2018 [29]	One minor limitation (9). Nine minor limitations (2, 4, 5, 6, 7, 10, 12, 14, 15).	USA
Ryan et al., 2018 [30]	One major limitation (9). Eight minor limitations (2, 3, 4, 7, 10, 12, 13, 16).	Appalachian states and also all states including Appalachian regions (USA)
Small et al., 2014 [31]	One major limitation (9). Nine minor limitations (2, 4, 5, 6, 7, 10, 12, 13, 14).	USA
Smulian et al., 2016 [32]	Two major limitations (4, 9). Ten minor limitations (2, 3, 5, 6, 7, 10, 12, 13, 14, 15).	N/R^2^
Walling et al., 2016 [33]	One major limitation (11). Seven minor limitations (2, 4, 5, 7, 10, 12, 14).	N/R^2^
Wigle et al., 2013 [34]	One major limitation (9). Ten minor limitations (2, 4, 5, 6, 7, 8, 10, 12, 13, 14).	Items identified by the search included studies and experiences from individual countries (Peru, Vietnam, Uganda, India, Rwanda, Ghana, Tanzania, Malaysia, Indonesia, Kenya, Bhutan, Bolivia, Cambodia, Haiti, Lesotho and Nepal) and broad world regions.
Young, 2010 [35]	One major limitation (9). Eight minor limitations (2, 4, 5, 6, 10, 12, 14, 16).	The 18 articles selected for inclusion in this review represent nine countries. Australia (*n* = 6) and China (*n* = 4) accounted for over half of the studies; India, Korea, Malaysia, New Zealand, Taiwan, Thailand, and Vietnam were also represented.

^1^As numbered in Additional file 4 which lists the criteria for assessing the reliability of reviews, adapted from AMSTAR 2

^2^*N/R:* not reported

#### Assessment of the reliability of included reviews

One overview author assessed the reliability of the individual reviews using an adapted version of A MeaSurement Tool to Assess systematic Reviews (AMSTAR 2) [36]. A second overview author verified the assessments. We adapted AMSTAR 2 to allow its application across the range of types of reviews included in this overview and to try to ensure its appropriateness to a scoping overview of this kind. This involved adapting the wording of some questions to allow assessment of reviews of descriptive studies, qualitative studies, surveys, and cost-effectiveness studies as well as reviews of studies of the effects of interventions. Adapting the wording of questions also involved simplifying the tool as we did not attempt to develop revised, comprehensive response categories for the adapted questions, as found in AMSTAR 2.

As we aimed to include as many relevant reviews as possible in this descriptive scoping overview, we assessed a review to have important methodological limitations only if it had one or more major methodological limitations or if, in the judgment of the overview authors, it had a large number of minor methodological limitations. A review was categorized as having a major limitation if it did not use a comprehensive literature search strategy, if it did not use a satisfactory technique for assessing the methodological limitations/risk of bias (RoB) for individual studies included in the review, or if it did not account for methodological limitations/RoB in individual studies when interpreting/discussing the results of the review. All other concerns were described as minor limitations. The adapted AMSTAR 2 criteria used in this overview are available in Additional file 4.

#### Data extraction and management

We designed a data extraction form (included as Additional file 5)  and piloted it on two reviews to test the form and ensure agreement on which content was to be extracted.

For each included review, one overview author extracted the following data, which were then verified by a second overview author:

- Review characteristics such as review objectives, number of included studies, proposed and included publication range, study designs, populations, geographic and health system settings, interventions, and comparison

Secondly, for reviews that did not have important limitations as assessed using the adapted AMSTAR 2 criteria listed in Additional file 4, we extracted data on the key findings and conclusions relevant to our overview question.

#### Assessment of the applicability of the evidence to LMICs

We assessed the applicability of the evidence to LMIC settings using an approach similar to that used in several recent Cochrane Effective Practice and Organisation of Care Group (EPOC) overviews of reviews [37]. This approach was also adapted to the range of reviews included in this scoping overview. Our assessment was based on the following questions:

- Were some or all of the studies included in the systematic review conducted in LMICs or were the findings in the review consistent across settings or time periods and therefore suggest wide applicability?
- Are there important differences in on-the-ground realities and constraints in LMICs, such as people’s access to health services or to communication sources that might substantially alter the feasibility and acceptability of the intervention (where applicable) or raise questions about the applicability of the review findings?
- Are there important differences in health system arrangements, such as how HPV vaccination is financed or delivered, that may mean an intervention could not work in the same way in LMICs or review findings may not be applicable to LMICs?

The methods for this scoping overview were specified in a protocol prior to the piloting of the study selection process. It included our overview questions, search strategy, inclusion and exclusion criteria, and the methods for a future reliability assessment. The protocol is available via the Open Science Framework (see https://osf.io/agzb4/).

## Results

**Fig. 1:**
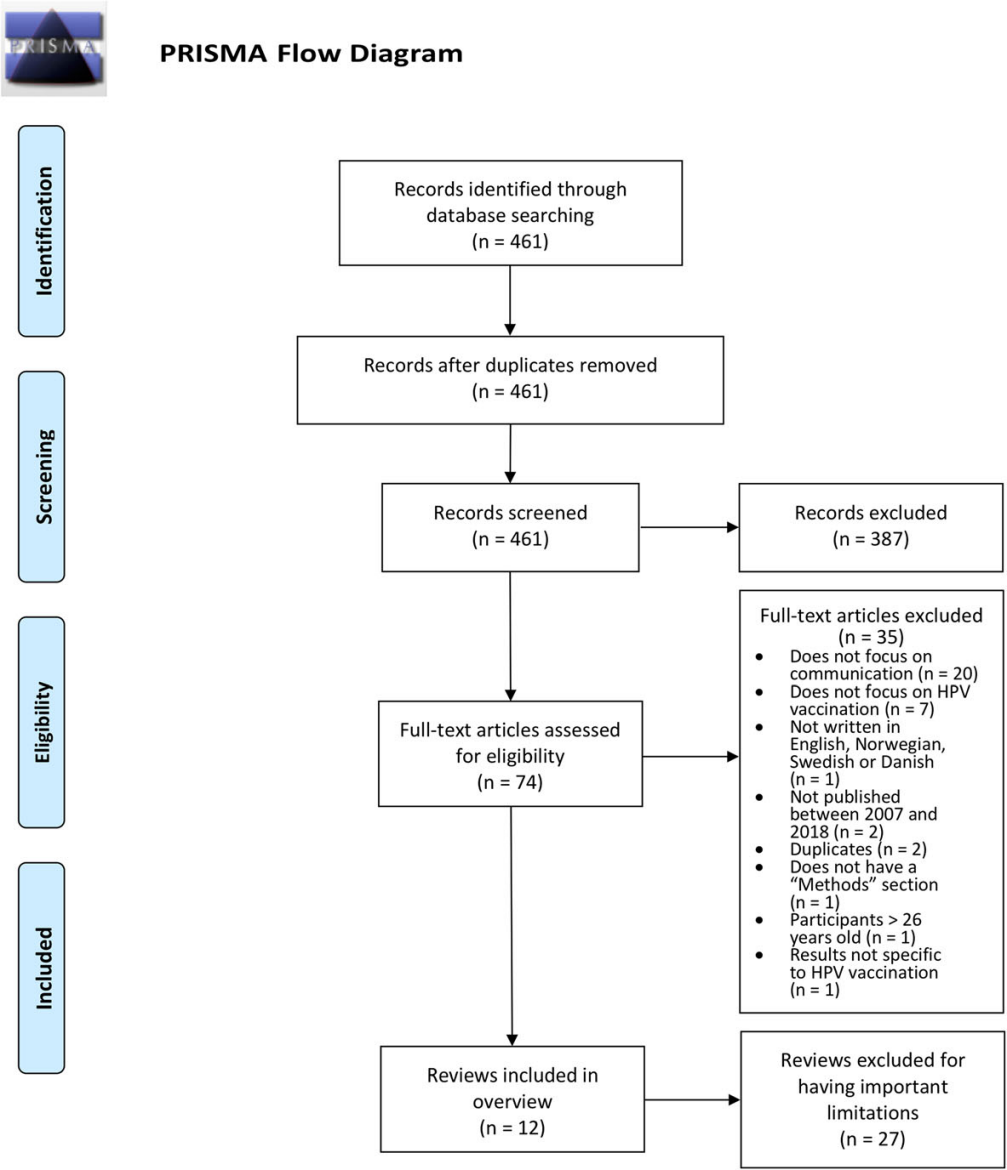
PRISMA Flow Diagram

Our search yielded 461 records. After screening titles and abstracts, we excluded 387 records. We assessed 74 reviews in full text of which 39 were potentially eligible for inclusion. Our assessment of methodological limitations led to the exclusion of a further 27 reviews, resulting in 12 included reviews. Additional file 3 lists the reasons for exclusion for reviews assessed in full text and Table 1 describes the methodological limitations of the reviews excluded because of concerns regarding their reliability (see also Additional file 6 for a list of reviews for which no full text was available). A PRISMA Flow Diagram [38] is included as Fig. 1.

### Summary description of included reviews

A summary of the characteristics of the studies included in the reviews can be found in Table 2. The number of included studies included in the reviews ranged from five to 79, and over half of the reviews included over 20 studies. The years of publication for studies included in the reviews ranged from 1996 to 2017. Over half of the reviews only found eligible studies published after 2008. The types of studies found included quantitative designs, such as randomized controlled trials, quasi-experimental designs, cross-sectional studies and surveys, and studies that used qualitative methods. The types of studies that were included in each review differed based on the review aim. The populations of the studies were mostly adolescent females, but some reviews included other stakeholders such as parents [45, 47, 48]. The geographic settings were mostly high-income countries (HICs) (see Table 2). Where reported, the health systems settings were mostly primary care [4, 40–42, 44, 45, 48, 49]. The reviews also found studies conducted in educational settings such as schools and universities [4, 40–42, 44, 49]. Further information on the funding and conflicts of interests reported for the included reviews is provided in Additional file 7.

**Table 2:** Table of characteristics of reviews with only minor methodological limitations

Review	*N*	Years of publication of included studies	Study designs^1^	Interventions or strategies^3^	Populations^2^	Geographical settings
Abdullahi et al., 2016 [39]	18	2008–2014	Cross-sectional: 17. Qualitative: 1	Sensitization campaigns and communication strategies: 3	Adolescent: 9 Parents: 14 Teachers: 2	South Africa: 6. Cameroon: 2. Uganda: 2. Nigeria: 2. Kenya: 1. Ghana: 1. Tanzania: 1. Botswana: 1. Mali: 1. Malawi: 1
Badawy et al., 2017 [40]	19	2010–2016	RCT: 11. Pre-post pilot design: 6. QES: 2	Reminder: 15 Mobile phone app platform intervention: 4	Adolescents (12-24): 19	USA: 9. Switzerland: 3. Hong Kong: 2. Colombia: 1. Wales: 1. Italy: 1. New Zealand: 1. Germany: 1
Ferrer et al., 2014 [41]	41	2004–2012	N/R^4^	N/A^5^	N/R^4^	USA: 24. Uke: 9. Australia: 3. Sweden: 2. Hong Kong. 2. Canada: 1
Fu et al., 2014 [4]	33	2004–2013	RCT: 10 N/R^4^: 23	Educational intervention: 15 Comparative message persuasiveness: 18	Parents: 12. Parents of girls: 5. Parents of either sex: 2. Adolescents (12-26): 21. Only women: 7. Only men: 3. Both: 3 Adults: 1	USA: 21. Canada: 3. Australia: 2. England: 2. China: 1. India: 1. Ireland: 1. Hong Kong: 1. Sweden: 1
Hendry et al., 2013 [42]	72	2004–2011	Qualitative: 28. Surveys: 44	N/A^5^	Adolescents (7-26) or their parents. Mostly women	USA: 32. UK: 15. Australia: 4. Malaysia: 3. India: 3. Canada: 3. Hong Kong: 2. Sweden: 2. Italy: 2. Thailand: 1. Brazil: 1. Vietnam: 1. Netherlands: 1. Korea: 1. Korea, Taiwan, Thailand and Malaysia: 1
Johnson et al., 2018 [43]	53	1996–2017	Cross-sectional: 34 Pre-posttest: 10 RCT: 8 Non-RCT: 1	Education strategies: 38 Restructure strategies: 26 Quality strategies: 13	N/R^4^	Southern Africa: 16. Western Africa: 16. Eastern Africa: 14. Middle Africa: 7
Kang et al., 2018 [44]	5	2013–2016	RCT: 3 Cluster RCT: 2	Reminders: 5 Reminders and education: 3	Mostly female adolescents (9-26) One study included males (11-17)	USA
Kim et al., 2017 [45]	22	2009–2015	Quantitative: 16 Qualitative 6	A culturally tailored Spanish educational radionovela	Parents, mostly of female adolescents: 22 Only mothers (18-64): 15	USA
Newman et al., 2013 [46]	24	N/R^4^	Cross-sectional studies: 27. Cohort studies: 2	N/A^5^	Adult men: 21 Boys (14-19): 2	USA: 12. Australia: 3. Sweden: 2. Canada: 1. Germany: 1. Netherlands: 1. New Zealand: 1. Philippines: 1. Singapore: 1. South Korea: 1
Newman et al., 2018 [47]	79	2009–2017	Cross-sectional: 67 Longitudinal: 7 Cohort: 1 Case-control: 1 QES: 1 Clustered non-RCT: 1 Cluster-RCT: 1	N/A^5^	Parents of girls: 45 Parents of boys: 10 Parents of either: 24 Sex of parents: Both: 44. Mothers: 24. Not specified: 11	USA: 55. Canada: 4. Denmark: 2. Norway: 2. Puerto Rico: 2. Australia: 1. Fiji: 1. Hong Kong: 1. Italy: 1. Kenya: 1. South Africa: 1. Tanzania: 1. Turkey: 1. United Arab Emirates: 1. Vietnam: 1
Radisic et al., 2016 [48]	18	2010–2015	Quantitative: 14 Qualitative: 3 Mixed-methods: 1	N/A^5^	Parents, mostly of male adolescents (9-26)	USA: 12. Canada: 2. Italy: 2. Denmark: 2
Rambout et al., 2013 [49]	22	2008–2011	Quantitative: 19 Qualitative: 1 Mixed-methods: 2	N/A^5^	Adolescents (26 years or younger)	USA: 21. Canada: 1

^1^The study design terms listed here are those reported in the individual reviews. They are therefore not consistent across reviews

^2^The ages of the participants have been included when reported

^3^This column reports only the number of studies of interventions of strategies included in the reviews. Not all reviews included interventions as some focused on stakeholders’ views of communication interventions and of factors affecting their implementation

^4^*N/R:* not reported

^5^*N/A:* not applicable

### Focus of the included reviews

**Fig. 2:**
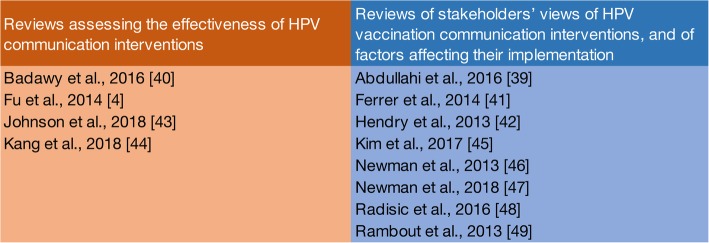
Overview of categorization of reviews

In relation to our overview objectives, we found that the available evidence from reviews on communication with stakeholders around HPV vaccination for adolescents could be organized into two groups, as shown in Fig. 2.

The first group assesses the effectiveness of HPV vaccination communication interventions or strategies and includes four reviews [4, 40, 43, 44]. The second group assesses factors associated with vaccination uptake as part of stakeholders’ views of HPV vaccination communication interventions or strategies and factors affecting their implementation. It includes eight reviews [39, 41, 42, 45–49].

For the following categories defined in the protocol, we found no reviews that met our eligibility criteria:

- HPV vaccination communication issues or problems identified by any of the stakeholders, such as people’s information needs and how they would like to receive that information (rather than stakeholders’ views of vaccination communication interventions)
- The cost or cost-effectiveness of HPV vaccination communication interventions or strategies

The concepts assessed by the included reviews were often poorly defined, not defined at all or differed across reviews. For example, one review [48] conceptualized vaccination acceptability as “holding a positive intention or willingness towards vaccinating sons in the future (vaccine intention), or by having consented to their sons being vaccinated in the past (vaccine initiation).” Another review [43] defined acceptability as “Perception among implementation stakeholders that a given treatment, service, practice, or innovation is agreeable, palatable, or satisfactory.” Differences such as these made assessing the focus of each review more challenging.

#### Reviews of the effectiveness of HPV vaccination communication interventions or strategies

Four reviews [4, 40, 43, 44] assessed the effectiveness of communication strategies by assessing their impact on vaccination acceptability, intention, or uptake. Their aims are described in Table 3. One review had a broader focus than HPV vaccination communication and included only a single study addressing HPV vaccination [40].

**Table 3:** Aims of the reviews of the effectiveness of HPV vaccination communication interventions or strategies

Review	Stated aims of the reviews
Badawy et al., 2016 [40]	Evaluate the efficacy of text messaging and mobile phone app interventions to improve adherence to preventive behavior, describe intervention approaches
Fu et al., 2014 [4]	Focus on educational interventions designed to increase HPV vaccine acceptance
Johnson et al., 2018 [43]	Uncover breadth and diversity of implementation strategies used to improve the uptake and sustainability of cervical cancer prevention programs
Kang et al., 2018 [44]	Evaluate the impact of interventions implemented after the first dose of HPV vaccination on the rate of HPV vaccine completion

The communication interventions identified in these reviews were nearly all interventions to inform or educate [50], including written information fact sheets, house-to-house education, and radio [4, 43] (see Table 4). Other communication interventions intended to inform or educate, and remind or recall and included reminder messages and education as well as DVD-based instructions with telephone reminders. Table 4 shows the outcomes assessed by these reviews. Findings regarding the effectiveness of the communication interventions are not discussed in this overview.

**Table 4:** Outcomes assessed by reviews on the effectiveness of HPV vaccination communication interventions

Reviews	Outcomes assessed	Communication intervention purpose^1^	Details of intervention
[4]	Vaccine acceptability, uptake	Inform or educate	Written information fact sheets from 1 to 2 pages in length
[4]			Not fact sheet based
[4]			1-h slide presentation
[4]			Videos ranging in length from 3 to 10 min
[4]			Hour-long, live presentations delivered at school
[4]			An online fact sheet with a question-and-answer section and a self-quiz
[4]			Spanish-language radio advertisement (referred to as radionovela in the study)
[43]			Community outreach with lectures, pamphlets, posters, radio messages, and dramas
[43]	Vaccine uptake		House-to-house education given on a one-on-one basis by community health workers
[43]			Staff training in program policy, sensitize school leadership, community outreach
[43]	Vaccine acceptability		Educational session to inform adults and adolescents
[44]	Completion rate	Inform or educate and remind or recall	Reminder messages and education
[44]			Reminder letters vs. standard care
[44]			DVD-based instruction with telephone reminder vs. standard care
[40]			Text messages

^1^Categories based on the “Communicate to Vaccinate” taxonomy of communication interventions for childhood vaccination [50]

#### Reviews of factors associated with HPV vaccination uptake

Eight reviews assessed factors associated with or influencing HPV vaccination uptake [39, 41, 42, 45–49] but did not look at the effectiveness of interventions to improve vaccination uptake. Seven of these reviews found only or mostly quantitative studies or surveys [39, 42, 45–49] while one review only searched for qualitative studies [41]. Some of the factors identified, such as knowledge and awareness, may be categorized as both barriers and facilitators in reviews, which is why we have decided to use the term “factor” to describe these.

Seven reviews in this group identified studies assessing factors thought to affect vaccination uptake and the association between these factors and uptake of the vaccine [41, 42, 45–49]. One review only identified studies that measured knowledge, attitudes, and practices and did not assess directly the association between these factors and reported uptake of the vaccine [39]. Rather, they hypothesized that these factors would impact on uptake.

The stated aims of the reviews are shown in Table 5. We have divided these reviews into two groups: the first group includes those looking at attitudes, views and preferences, and acceptability with regard to HPV vaccination (*n* = 4) [39, 42, 46, 48]. The second group includes those reviews focused on factors affecting vaccination uptake (*n* = 4) [41, 45, 47, 49].

**Table 5:** Aims of the reviews looking at factors associated with vaccination uptake

Systematic reviews looking at attitudes, views and preferences and acceptability with regard to HPV vaccination	Stated aim
Abdullahi et al., 2016 [39]	Knowledge, attitudes, and practices among stakeholders
Hendry et al., 2013 [42]	Information needs, views, and preferences
Newman et al., 2013 [46]	Acceptability and factors correlated with acceptability
Radisic et al., 2016 [48]	Factors associated with acceptability in parents of adolescent boys
Systematic reviews focusing on factors affecting vaccination uptake	Stated aim
Ferrer et al., 2014 [41]	Facilitators and barriers to decision-making by key stakeholders
Kim et al., 2017 [45]	Awareness, intention, and uptake among immigrant parents
Newman et al., 2018 [47]	Parents’ uptake, examine factors correlated with parents’ uptake, possible moderating influences of sex of child and parent on uptake
Rambout et al., 2013 [49]	Barriers and facilitators to vaccination

In the first group of reviews, two aimed to explore the attitudes, views, or preferences of stakeholders towards HPV vaccination and included mainly survey studies [39, 42]. One of these reviews looked at knowledge, attitudes, and practices among stakeholders [39], while a second review looked at stakeholders’ information needs, views, and preferences [42]. Two more reviews assessed the acceptability of HPV vaccination [46, 48]. One review looked at acceptability in parents of adolescent boys [48] and included mostly survey studies. A second review explored HPV vaccine acceptability, and factors linked to this, among men and included mostly cross-sectional, quantitative studies [46].

The four reviews in the second group looked at factors affecting HPV vaccination uptake [41, 45, 47, 49]. Two of the reviews [45, 49] considered uptake as well as intention to vaccinate and included mostly survey and other cross-sectional studies. One review [41] included only qualitative studies of vaccination decision-making (which we viewed as related to intention to vaccinate), with the aim of providing an understanding of the factors affecting vaccination uptake. The last review [47] included only quantitative studies and found mostly studies using cross-sectional designs. It looked at factors associated with parents’ uptake of HPV vaccines for their children.

Table 6 provides a summary of the range of factors assessed in reviews of stakeholders’ views of HPV vaccination communication interventions and of the factors affecting the implementation of these interventions. The most commonly assessed factors were HPV education, health care provider influence, and the perceived benefits of HPV vaccination.

**Table 6:** Summary of the range of factors assessed in reviews of stakeholders’ views of HPV vaccination communication interventions and of factors affecting the implementation of these interventions

Overview authors’ groupings of factors	Factors as specified in reviews^1^	Outcomes used in quantitative studies to measure factors affecting stakeholders’ views and the implementation of HPV vaccination communication^2^	Reviews	Number of reviews in this grouping
HPV education	Knowledge [39, 48], information needs [42], HPV awareness [46], parents’ HPV-related knowledge and awareness—HPV vaccine knowledge and awareness [47], cervical cancer / HPV knowledge [47]	Level of knowledge [39, 42], acceptability [46, 48], uptake [47]	[39, 42, 46–48]	5
Health care provider influence	Physician recommendation [42], provider’s influence [45], health care provider recommendation [49]	Positive/negative [49], acceptability [42, 46], intention, uptake [45, 47]	[42, 45–47, 49]	5
Perceived benefits of HPV vaccination	Perceived HPV vaccine benefits [46–49]	Attitudes, acceptance [46, 48], intention or uptake [49], uptake [47]	[46–49]	4
Attitudes and beliefs	Parents’ vaccine attitudes—HPV vaccine safety concerns [47], fear of side effects/safety [48]	Acceptability [48], uptake [47]	[47–49]	3
	Anticipatory regret [46, 47]	Attitudes, uptake [46, 47]	[46, 47]	2
	Feeling that the vaccine was not needed for various reasons [49]	Positive/negative [49]	[49]	1
	Perceived HPV vaccine effectiveness [46]	Attitudes [46]	[46]	1
	Fear of needles [46]	Attitudes [46]	[46]	1
	Belief in vaccines in general	Uptake [47]	[47]	1
Structural barriers	Vaccine cost [45–47, 49]	Acceptability [46], intention or uptake [49], uptake [45, 47, 49]	[45–47, 49]	4
	Perceived HPV severity [46]	Acceptability [46]	[46]	1
	Logistical barriers [46]	Acceptability [46]	[46]	1
	Need for multiple shots/doses [46]	Acceptability [46]	[46]	1
Acceptability	Acceptability [42, 46]	Level of acceptability [42, 46]	[42, 46]	2
	Parent’s vaccine beliefs, attitudes and intentions—intention to vaccinate child for HPV [47]	Uptake [47]	[47]	1
Sexual risk behavior	Number of lifetime sexual partners [46]	Acceptability [46]	[46]	1
	Having a current sex partner [46]	Acceptability [46]	[46]	1
	History of STI [46]	Acceptability [46]	[46]	1
	Not being sexually active [49]	Positive/negative [49]	[49]	1
Other risk behaviors	Smoking cigarettes [46]	Acceptability [46]	[46]	1
	Non-receipt of hepatitis B vaccine [46]	Acceptability [46]	[46]	1
Socio-demographic factors	Effects of neighborhoods [45]	Uptake [45]	[45]	1
	Acculturation (e.g., language use, origin of birth) [45]	HPV vaccination [45]	[45]	1
	Being employed [46]	Acceptability [46]	[46]	1
	Educational level [46]	Acceptability [46]	[46]	1
	Non-white (vs white) ethnicity [46]	Acceptability [46]	[46]	1
HPV vaccine endorsements	Endorsement from a governmental source [41]	N/A [41]	[41]	1
	Partner thinks one should get the vaccine [46]	Acceptability [46]	[46]	1
Other	Cultural sexual health values (such as social norms regarding adolescent sexuality and stigma related to sexually transmitted diseases) [45]	N/A [45]	[45]	1
	Immigration laws [45]	N/A [45]	[45]	1
	Mother as HPV vaccine decision-maker (vs both parents) [47]	Uptake [47]	[47]	1
	Social norms [49]	Intention or uptake [49]	[49]	1

^1^Not all reviews in each group assessed all factors

^2^Not all reviews in each group assessed all outcomes

Additional file 8 shows the aims of the included reviews, in relation to our overview objectives, and the results as expressed by the review authors. We do not discuss the review results as this falls outside of the scope of this overview.

### Applicability of the evidence

Our assessments of the applicability of the evidence in the reviews to LMICs shows that this differs among the included reviews (see Table 7). With two exceptions [39, 43], the reviews included few studies from LMICs. For many reviews, we believe there are likely to be important differences in on-the-ground realities and constraints in LMICs that might alter the feasibility and acceptability of the intervention or raise questions about the applicability of the review findings. For many reviews, we also assessed there to be important differences in health system arrangements that may mean an intervention would not work in the same way in LMICs or review findings may not be applicable to LMICs. The reasoning behind these assessments can be found in Additional file 9.

**Table 7:** Applicability of the evidence to LMICs

Review	Were some or all of the studies included in the systematic review conducted in LMICs or were the findings in the review consistent across settings or time periods and therefore suggest wide applicability? [Proportion of studies conducted in LMICs]	Are there important differences in on-the-ground realities and constraints in LMICs that might substantially alter the feasibility and acceptability of the intervention (where applicable) or raise questions about the applicability of the review findings?	Are there important differences in health system arrangements that may mean an intervention could not work in the same way in LMICs or review findings may not be applicable to LMICs?
Abdullahi et al., 2016 [39]	Yes [18/18]	Likely no	Largely not applicable
Badawy et al., 2017 [40]	No^1^	Likely yes	Yes
Ferrer et al., 2014 [41]^2^	No	Likely yes	Likely yes
Fu et al., 2014 [4]^2^	Yes [2/33]	Likely yes	Likely yes
Hendry et al., 2013 [42]	Yes [11/72]	Likely no	Likely no
Johnson et al., 2018 [43]	Yes [53/53]	Likely no	Likely no^2^
Kang et al., 2018 [44]	No	Likely yes	Likely yes
Kim et al., 2017 [45]	No	Likely no	Likely no
Newman et al., 2013 [46]	Yes [2/29]	Likely yes	Likely yes
Newman et al., 2018 [47]	Yes [6/79]	Likely no	Likely no
Radisic et al., 2017 [48]	No	Yes	Likely yes
Rambout et al., 2014 [49]^3^	No	Likely yes	Likely yes

^1^The review includes one study from a LMIC; however, this study does not focus on HPV

^2^Difficult to assess due to inadequate reporting of the results

^3^Did not intend to include studies from LMICs

### Methodological limitations of included reviews

All of the 12 included reviews had minor methodological limitations. We assessed 11 reviews to have used only a partially comprehensive literature search strategy. Ten reviews did not contain an explicit statement that the review methods were established prior to the conduct of the review. Ten reviews also did not provide a list of excluded studies or justify their exclusions. Nine of the included reviews did not report on the sources of funding for their included studies. Seven reviews did not perform data extraction in duplicate and five reviews failed to adequately describe or perform study selection in duplicate.

Table 1 summarizes the AMSTAR 2 assessments of the reviews excluded for having major methodological limitations or a large number of minor methodological limitations. Table 8 provides these assessments for the included reviews. Additional file 10 provides the full AMSTAR 2 assessments.

**Table 8:** Table of methodological limitations of included reviews

Review	Number of methodological limitations (criterion numbers^1^)
Abdullahi et al., 2016 [39]	Four minor limitations (3, 4, 10, 14)
Badawy et al., 2017 [40]^2^	One major limitation (9). Six minor limitations (4, 7, 10, 11, 12, 14)
Ferrer et al., 2014 [41]	Seven minor limitations (2, 4, 6, 7, 10, 12, 14)
Fu et al., 2014 [4]	Five minor limitations (2, 3, 4, 7, 10)
Hendry et al., 2013 [42]	Seven minor limitations (2, 3, 4, 6, 7, 12, 13)
Johnson et al., 2018 [43]	Eight minor limitations (2, 3, 4, 6, 7, 9, 10, 14)
Kang et al., 2018 [44]	Seven minor limitations (2, 3, 4, 5, 6, 7, 10)
Kim et al., 2017 [45]	Eight minor limitations (2, 4, 5, 6, 7, 10, 13, 14)
Newman et al., 2013 [46]	Eight minor limitations (2, 3, 4, 5, 6, 7, 10, 15)
Newman et al., 2018 [47]	Four minor limitations (2, 3, 4, 7)
Radisic et al., 2017 [48]	Six minor limitations (2, 4, 5, 6, 7, 10)
Rambout et al., 2014 [49]	Five minor limitations (2, 4, 5, 7, 14)

^1^As numbered in Additional file 4 which lists the criteria for assessing the reliability of reviews, adapted from AMSTAR 2

^2^This review did not include assessments of the methodological limitations of each included study and therefore could be viewed as having a major limitation, according to our assessment tool. However, the overall results from the assessments were reported and the review was therefore included in our overview on this basis

## Discussion

### Summary of main results

This scoping overview aimed to provide a broad overview of the evidence available on communication with stakeholders around HPV vaccination for adolescents, with a specific focus on LMICs. We included 12 reviews in the overview after excluding 27 eligible reviews because of important methodological limitations. Of these 12 reviews, four reviews [4, 40, 43, 44] assessed the effectiveness of communication strategies by assessing their impact on vaccination acceptability, intention, or uptake. The interventions or strategies described intended to either *inform or educate* (including videos, live presentations, and fact sheets [4, 43, 44]) or to *remind or recall* (including text messages, letters, and telephone reminders [40, 44]) regarding HPV vaccination. Eight reviews reported on factors associated with vaccination uptake and which may affect the implementation of communication interventions. The most commonly assessed factors were HPV education, health care provider influence, and the perceived benefits of vaccination. A plain language summary of these results is available in Additional file 11.

We experienced challenges in categorizing the reviews included in this overview because some reviews had more than one aim. In addition, some of the concepts used across reviews, such as attitudes towards HPV vaccination, were defined in different ways in these reviews. Future reviews might benefit from the development of a shared terminology and consistent definitions that can be applied across reviews.

### Overall completeness and applicability of evidence

In this scoping overview, we did not find any reviews addressing communication issues or communication problems related to HPV vaccination. Nor did we find any reviews on the cost or cost-effectiveness of HPV vaccination communication interventions or strategies.

Most of the included reviews found few studies from LMICs. Our assessment of the evidence that was identified in the reviews was that its applicability to LMICs differed (Table 7). However, we acknowledge that these assessments are subjective judgments and we recognize that others may reach different conclusions regarding the degree of differences in on-the-ground realities and health systems between high-income countries and LMICs in relation to the reviews assessed. The reasoning behind the assessments in Table 7 can be found in Additional file 9. Overall, the low numbers of studies from LMICs included in the reviews highlight an evidence gap in relation to primary studies of communication around HPV vaccination for adolescents.

### Potential biases in the scoping overview process

We conducted a search using the most comprehensive and up-to-date global database of systematic reviews [51] that is, in turn, based on searches of a very large number of other health study databases. However, we may have missed reviews that described their focus using terms other than those included in our search strategy. In addition, we did not hand search the references of the included reviews, identify eligible gray literature, or ask experts in the field for eligible reviews. These limitations mean that we may not have identified all existing eligible reviews.

Although the overview only included reviews written in English, Norwegian, Swedish, or Danish, only one review was excluded at the full-text stage for being published in another language (Portuguese).

One of the included reviews only included one study regarding HPV vaccination. Although this is a deviation from our protocol, we decided to include this review as the included study contributed to the overview aim. However, this could be a potential source of bias as other similar reviews could have been excluded by our inclusion criteria.

Strengths of the overview include the exclusion of reviews with important methodological limitations that may have misleading results and verification by a second overview author of both the data extraction and the adapted AMSTAR 2 assessments.

### Agreements and disagreements with other studies or reviews

We did not find any other systematic scoping overviews or other overview of reviews with which we could compare our results.

## Conclusions

Twelve reviews were included in this overview: four reviews assessed their effectiveness while eight reviews assessed factors associated with HPV vaccination uptake. Out of the 12 included reviews, nine reviews searched for studies from LMICs. However, most of these found only a small number of studies from these countries. The small number of studies from LMICs is of concern as these countries face the largest burden of disease related to HPV. We excluded a number of existing reviews because of important methodological limitations. This highlights the need for future reviews to use appropriate methods and to adhere to reporting standards such as PRISMA [38].

Our findings suggest that there may still be important knowledge gaps, for example, in relation to educational interventions to increase HPV acceptance and studies of HPV vaccination for males. Further primary research in these areas may be needed. We also identified gaps in relation to reviews of issues or problems identified by any of the stakeholders and regarding the cost or cost-effectiveness of HPV vaccination communication interventions or strategies. New reviews in these areas may be helpful. These reviews may indicate areas in which further primary studies are needed on HPV vaccination communication in LMICs.

## Additional files

Additional file 1: PRISMA-ScR Checklist. (PDF 82 kb)

Additional file 2: Full search strategy. (PDF 13 kb)

Additional file 3: List of excluded reviews assessed in full-text with reasons for exclusion. (PDF 47 kb)

Additional file 4: Criteria for assessing the reliability of included reviews. (PDF 66 kb)

Additional file 5: Data extraction sheet. (XLSX 54 kb)

Additional file 6: List of reviews excluded where no full-text was available. (PDF 23 kb)

Additional file 7: Table of funding and conflicts of interest as reported in the reviews. (PDF 38 kb)

Additional file 8: Summary of the results of each review, in relation to the scoping overview objectives. (PDF 59 kb)

Additional file 9: Applicability of the evidence to LMICs, with explanations of assessments. (PDF 56 kb)

Additional file 10: Individual AMSTAR 2 assessments. (PDF 770 kb)

Additional file 11: Plain language summary of the overview. (PDF 37 kb)

## Data Availability

All data generated or analyzed during this study are included in this published article and its additional files.

## Abbreviations

AMSTAR 2: A MeaSurement Tool to Assess systematic Reviews 2
EPOC: Cochrane Effective Practice and Organisation of Care Group
HIC: High-income country
HPV: Human papillomavirus
LMICs: Low- and middle-income countries
N/A: Not applicable
N/R: Not reported
NIPH: Norwegian Institute of Public Health
PRISMA: Preferred Reporting Items for Systematic reviews and Meta-Analyses
RCT: Randomized controlled study
WHO: World Health Organization
