# Supplementary material for: Communication around HPV vaccination for adolescents in low- and middle-income countries: a systematic scoping overview of systematic reviews
Source: Syst Rev. 2019 Aug 1;8:190. doi: 10.1186/s13643-019-1100-y (PMC6670236; doi:10.1186/s13643-019-1100-y)
Supplement: Supplementary file 2 — Full search strategy. (PDF 13 kb) [file 13643_2019_1100_MOESM2_ESM.pdf]

Additional file 2: The full search strategy

https://www.epistemonikos.org/advanced_search?q=(title:((title:((HPV OR papilloma* OR "cervical cancer" OR "cervical cancers" OR "cervic cancer" OR "cervic cancers" OR "cervix cancer" OR "cervix cancers" OR "cervical neoplasm" OR "cervical neoplasms" OR "cervic neoplasm" OR "cervic neoplasms" OR "cervix neoplasm" OR "cervix neoplasms") AND (vacc* OR immun* OR communic* OR educat* OR inform* OR campaign*)) OR abstract:((HPV OR papilloma* OR "cervical cancer" OR "cervical cancers" OR "cervic cancer" OR "cervic cancers" OR "cervix cancer" OR "cervix cancers" OR "cervical neoplasm" OR "cervical neoplasms" OR "cervic neoplasm" OR "cervic neoplasms" OR "cervix neoplasm" OR "cervix neoplasms") AND (vacc* OR immun* OR communic* OR educat* OR inform* OR campaign*)))) OR abstract:((title:((HPV OR papilloma* OR "cervical cancer" OR "cervical cancers" OR "cervic cancer" OR "cervic cancers" OR "cervix cancer" OR "cervix cancers" OR "cervical neoplasm" OR "cervical neoplasms" OR "cervic neoplasm" OR "cervic neoplasms" OR "cervix neoplasm" OR "cervix neoplasms") AND (vacc* OR immun* OR communic* OR educat* OR inform* OR campaign*)) OR abstract:((HPV OR papilloma* OR "cervical cancer" OR "cervical cancers" OR "cervic cancer" OR "cervic cancers" OR "cervix cancer" OR "cervix cancers" OR "cervical neoplasm" OR "cervical neoplasms" OR "cervic neoplasm" OR "cervic neoplasms" OR "cervix neoplasm" OR "cervix neoplasms") AND (vacc* OR immun* OR communic* OR educat* OR inform* OR campaign*)))))&protocol=no&classification=systematic-review&min_year=2007&max_year=2017
