# Supplementary material for: Communication around HPV vaccination for adolescents in low- and middle-income countries: a systematic scoping overview of systematic reviews
Source: Syst Rev. 2019 Aug 1;8:190. doi: 10.1186/s13643-019-1100-y (PMC6670236; doi:10.1186/s13643-019-1100-y)
Supplement: Supplementary file 3 — List of excluded reviews assessed in full-text with reasons for exclusion. (PDF 47 kb) [file 13643_2019_1100_MOESM3_ESM.pdf]

Additional file 3: List of excluded reviews assessed in full-text with reasons for exclusion

Review	Reason for exclusion
**de Andrade, 2010 [1]**	Not written in English, Norwegian, Swedish or Danish.
**Baroy et al., 2016 [2]**	Does not focus on HPV vaccination.
**Ben Hadj Yahia et al., 2015 [3]**	Does not focus on communication.
**Bird et al., 2017 [4]**	Does not focus on communication.
**Black et al., 2002 [5]**	Not published between 2007 and 2018.
**de Casadevante et al., 2015 [6]**	Does not focus on communication.
**Coles et al., 2015 [7]**	Does not focus on communication.
**Corcoran et al., 2014 [8]**	Does not focus on communication.
**Drolet et al., 2015 [9]**	Does not focus on communication.
**Finocchario-Kessler et al., 2016 [10]**	Does not focus on HPV vaccination.
**Fisher et al., 2013 [11]**	Does not focus on communication.
**Gallagher et al., 2016 [12]**	Does not focus on communication.
**Garcini et al., 2012 [13]**	Does not focus on communication.
**Han et al., 2011 [14]**	Does not focus on HPV vaccination.
**Islam et al., 2017 [15]**	Does not focus on HPV vaccination.
**Jarrett et al., 2015 [16]**	Results not specific to HPV vaccination.
**Kasting et al., 2016 [17]**	Does not focus on communication.
**Kasting et al., 2016 [18]**	Does not focus on communication.
**Katz et al., 2010 [19]**	Does not have a “Methods” section.
**Kaufman et al., 2013 [20]**	Does not focus on HPV vaccination.
**Macki et al., 2016 [21]**	Does not focus on HPV vaccination.
**Madhivanan et al., 2016 [22]**	Does not focus on communication.
**Marlow, 2011 [23]**	Does not focus on communication.
**Maver et al., 2017 [24]**	Does not focus on communication.
**Newman et al., 2012 [25]**	Duplicate.
**Nadarzynski et al., 2014 [26]**	Participants >26 years old.
**Patel et al., 2016 [27]**	Does not focus on communication.
**Posadzki et al., 2016 [28]**	Does not focus on HPV vaccination.
**Prue et al., 2016 [29]**	Does not focus on communication.
**Rambout et al., 2012 [30]**	Duplicate.
**Ratanasiripong, 2012 [31]**	Does not focus on communication.
**Santhanes et al., 2018 [32]**	Does not focus on communication.
**Stone et al., 2002 [33]**	Not published between 2007 and 2018.
**Trim et al., 2012 [34]**	Does not focus on communication.
**Zhang et al., 2016 [35]**	Does not focus on communication.

1. Andrade CJCd. Economic evaluations of the use of a vaccine against human papillomavirus (HPV) in adolescent girls: a systematic review. 2010:123-.

2. Baroy J, Chung D, Frisch R, Apgar D, Slack MK. The impact of pharmacist immunization programs on adult immunization rates: A systematic review and meta-analysis. Journal of the American Pharmacists Association : JAPhA. 2016;56(4):418-26.

3. Ben Hadj Yahia MB, Jouin-Bortolotti A, Dervaux B. Extending the Human Papillomavirus Vaccination Programme to Include Males in High-Income Countries: A Systematic Review of the Cost-Effectiveness Studies. Clin Drug Investig. 2015;35(8):471-85.

4. Bird Y, Obidiya O, Mahmood R, Nwankwo C, Moraros J. Human Papillomavirus Vaccination Uptake in Canada: A Systematic Review and Meta-analysis. Int J Prev Med. 2017;8:71.

5. Black ME, Yamada J, Mann V. A systematic literature review of the effectiveness of community-based strategies to increase cervical cancer screening. Can J Public Health. 2002;93(5):386-93.

6. Fernández de Casadevante V, Gil Cuesta J, Cantarero-Arévalo L. Determinants in the Uptake of the Human Papillomavirus Vaccine: A Systematic Review Based on European Studies. Front Oncol. 2015;5:141.

7. Coles VA, Patel AS, Allen FL, Keeping ST, Carroll SM. The association of human papillomavirus vaccination with sexual behaviours and human papillomavirus knowledge: a systematic review. Int J STD AIDS. 2015;26(11):777-88.

8. Corcoran J, Crowley M. Latinas' attitudes about cervical cancer prevention: a meta-synthesis. J Cult Divers. 2014;21(1):15-21.

9. Drolet M, Bénard É, Boily M-C, Ali H, Baandrup L, Bauer H, et al. Population-level impact and herd effects following human papillomavirus vaccination programmes: a systematic review and meta-analysis. The Lancet Infectious Diseases. 2015;15(5):565-80.

10. Finocchario-Kessler S, Wexler C, Maloba M, Mabachi N, Ndikum-Moffor F, Bukusi E. Cervical cancer prevention and treatment research in Africa: a systematic review from a public health perspective. BMC Womens Health. 2016;16(1):29.

11. Fisher H, Trotter CL, Audrey S, MacDonald-Wallis K, Hickman M. Inequalities in the uptake of human papillomavirus vaccination: a systematic review and meta-analysis. Int J Epidemiol. 2013;42(3):896-908.

12. Gallagher KE, Kadokura E, Eckert LO, Miyake S, Mounier-Jack S, Aldea M, et al. Factors influencing completion of multi-dose vaccine schedules in adolescents: a systematic review. BMC Public Health. 2016;16:172.

13. Garcini LM, Galvan T, Barnack-Tavlaris JL. The study of human papillomavirus (HPV) vaccine uptake from a parental perspective: a systematic review of observational studies in the United States. Vaccine. 2012;30(31):4588-95.

14. Han HR, Kim J, Lee JE, Hedlin HK, Song H, Song Y, et al. Interventions that increase use of Pap tests among ethnic minority women: a meta-analysis. Psychooncology. 2011;20(4):341-51.

15. Islam RM, Billah B, Hossain MN, Oldroyd J. Barriers to Cervical Cancer and Breast Cancer Screening Uptake in Low-Income and Middle-Income Countries: A Systematic Review. Asian Pacific journal of cancer prevention : APJCP. 2017;18(7):1751-63.

16. Jarrett C, Wilson R, O'Leary M, Eckersberger E, Larson HJ, Hesitancy SWGoV. Strategies for addressing vaccine hesitancy - A systematic review. Vaccine. 2015;33(34):4180-90.

17. Kasting ML, Shapiro GK, Rosberger Z, Kahn JA, Zimet GD. Tempest in a Teapot: A Systematic Review of HPV Vaccination and Risk Compensation Research. Hum Vaccin Immunother. 2016;12(6):0.

18. Kasting M, Shapiro G, Rosberger Z, Kahn J, Zimet G. Does HPV vaccination impact sexual behaviour? A systematic literature review. Psychooncology. 2016:159.

19. Katz IT, Ware NC, Gray G, Haberer JE, Mellins CA, Bangsberg DR. Scaling up human papillomavirus vaccination: a conceptual framework of vaccine adherence. Sexual health. 2010;7(3):279-86.

20. Kaufman J, Synnot A, Ryan R, Hill S, Horey D, Willis N, et al. Face to face interventions for informing or educating parents about early childhood vaccination. Cochrane Database of Systematic Reviews. 2013;5(5):CD010038.

21. Macki M, Dabaja AA. Literature review of vaccine-related adverse events reported from HPV vaccination in randomized controlled trials. Basic and clinical andrology. 2016;26:16.

22. Madhivanan P, Pierre-Victor D, Mukherjee S, Bhoite P, Powell B, Jean-Baptiste N, et al. Human Papillomavirus Vaccination and Sexual Disinhibition in Females: A Systematic Review. Am J Prev Med. 2016;51(3):373-83.

23. Marlow LA. HPV vaccination among ethnic minorities in the UK: knowledge, acceptability and attitudes. Br J Cancer. 2011;105(4):486-92.

24. Maver PJ, Poljak M. Progress in prophylactic human papillomavirus (HPV) vaccination in 2016: A literature review. Vaccine. 2017.

25. Newman PA, Logie C, Asakura K. Meta-analysis of correlates of HPV vaccine acceptability among men: supporting vaccine implementation science. Retrovirology. 2012.

26. Nadarzynski T, Smith H, Richardson D, Jones CJ, Llewellyn CD. Human papillomavirus and vaccine-related perceptions among men who have sex with men: a systematic review. Sex Transm Infect. 2014;90(7):515-23.

27. Patel H, Jeve YB, Sherman SM, Moss EL. Knowledge of human papillomavirus and the human papillomavirus vaccine in European adolescents: a systematic review. Sex Transm Infect. 2016;92(6):474-9.

28. Posadzki P, Mastellos N, Ryan R, Gunn LH, Felix LM, Pappas Y, et al. Automated telephone communication systems for preventive healthcare and management of long-term conditions. Cochrane Database of Systematic Reviews. 2016;12:CD009921.

29. Prue G, Shapiro G, Maybin R, Santin O, Lawler M. Knowledge and acceptance of human papillomavirus (HPV) and HPV vaccination in adolescent boys worldwide: A systematic review. Journal of Cancer Policy. 2016;10:1-15.

30. Rambout L, Tashkandi M, Hopkins L, Tricco AC. Self-reported barriers and facilitators to preventive hpv vaccination among adolescent girls and young women: A systematic review. Int J Gynecol Cancer. 2012.

31. Ratanasiripong NT. A review of human papillomavirus (HPV) infection and HPV vaccine-related attitudes and sexual behaviors among college-aged women in the United States. Journal of American college health : J of ACH. 2012;60(6):461-70.

32. Santhanes D, Yong CP, Yap YY, Saw PS, Chaiyakunapruk N, Khan TM. Factors influencing intention to obtain the HPV vaccine in South East Asian and Western Pacific regions: A systematic review and meta-analysis. Sci Rep. 2018;8(1):3640.

33. Stone EG, Morton SC, Hulscher ME, Maglione MA, Roth EA, Grimshaw JM, et al. Interventions that increase use of adult immunization and cancer screening services: a meta-analysis. Ann Intern Med. 2002;136(9):641-51.

34. Trim K, Nagji N, Elit L, Roy K. Parental Knowledge, Attitudes, and Behaviours towards Human Papillomavirus Vaccination for Their Children: A Systematic Review from 2001 to 2011. Obstet Gynecol Int. 2012;2012:921236.

35. Zhang Y, Wang Y, Liu L, Fan Y, Liu Z, Wang Y, et al. Awareness and knowledge about human papillomavirus vaccination and its acceptance in China: a meta-analysis of 58 observational studies. BMC Public Health. 2016;16:216.
