# Supplementary material for: Communication around HPV vaccination for adolescents in low- and middle-income countries: a systematic scoping overview of systematic reviews
Source: Syst Rev. 2019 Aug 1;8:190. doi: 10.1186/s13643-019-1100-y (PMC6670236; doi:10.1186/s13643-019-1100-y)
Supplement: Supplementary file 4 — Criteria for assessing the reliability of included reviews. (PDF 66 kb) [file 13643_2019_1100_MOESM4_ESM.pdf]

**Additional file 4: Criteria for assessing the reliability of included reviews (adapted from AMSTAR 2[1])**

**1. Did the review have a clear research question and inclusion criteria?** Yes/No

**2. Did the report of the review contain an explicit statement that the review methods were established prior to the conduct of the review and did the report justify any significant deviations from the protocol?** Yes/No

**3. Did the review authors explain their selection of the study designs for inclusion in the review?** Yes/No

**4. Did the review authors use a comprehensive literature search strategy?**

| For Partial Yes (all the following): | For Yes, should also have (all the following): | |
|---|---|---|
| ☐ searched at least 2 databases (relevant to research question) | ☐ searched the reference lists / bibliographies of included studies | ☐ Yes<br>☐ Partial Yes<br>☐ No |
| ☐ provided key word and/or search strategy | ☐ included/consulted content experts in the field | |
| ☐ justified publication restrictions (e.g. language) | | |
| | ☐ where relevant, searched for grey literature | |
| | ☐ conducted search within 24 months of completion of the review | |

**5. Did the review authors perform study selection in duplicate?**

**6. Did the review authors perform data extraction in duplicate?**

**7. Did the review authors provide a list of excluded studies and justify the exclusions?**

| For Partial Yes: | For Yes, must also have: | |
|---|---|---|
| ☐ provided a list of all potentially relevant studies that were read in full-text form but excluded from the review | ☐ Justified the exclusion from the review of each potentially relevant study | ☐ Yes<br>☐ Partial Yes<br>☐ No |

**8. Did the review authors describe the included studies in adequate detail?**

**9. Did the review authors use a satisfactory technique for assessing the methodological limitations / risk of bias (RoB) in individual studies that were included in the review?**

**10. Did the review authors report on the sources of funding for the studies included in the review?**

**11. If a synthesis was performed did the review authors use appropriate methods to combine the results of individual studies?**

**12. If synthesis was performed, did the review authors assess the potential impact of the methodological limitations / RoB in individual studies on the results of the evidence synthesis?**

**13. Did the review authors account for methodological limitations / RoB in individual studies when interpreting/ discussing the results of the review?**

**14. Did the review authors provide a satisfactory explanation for, and discussion of, any heterogeneity observed in the results of the review? (*Omit for QES and for some reviews of survey data*)**

**15. If they performed quantitative synthesis did the review authors carry out an adequate investigation of publication bias (small study bias) and discuss its likely impact on the results of the review? (*Omit for QES*)**

**16. Did the review authors report any potential sources of conflict of interest, including any funding they received for conducting the review?**

1. Shea BJ, Reeves BC, Wells G, Thuku M, Hamel C, Moran J, Moher D, Tugwell P, Welch V, Kristjansson E, Henry DA. AMSTAR 2: a critical appraisal tool for systematic reviews that include randomised or non-randomised studies of healthcare interventions, or both. BMJ. 2017;358.
