# Supplementary material for: Communication around HPV vaccination for adolescents in low- and middle-income countries: a systematic scoping overview of systematic reviews
Source: Syst Rev. 2019 Aug 1;8:190. doi: 10.1186/s13643-019-1100-y (PMC6670236; doi:10.1186/s13643-019-1100-y)
Supplement: Supplementary file 6 — List of reviews excluded where no full-text was available. (PDF 23 kb) [file 13643_2019_1100_MOESM6_ESM.pdf]

Additional file 6: List of reviews excluded where no full-text was available

| Review | Reason for exclusion |
|---|---|
| **Adedoyin, 2014 [1]** | No full-text available. |
| **Fernandez, 2016 [2]** | No full-text available – poster abstract. |
| **Kiekenbush, 2012 [3]** | No full-text available. |
| **Kuk et al., 2015 [4]** | No full-text available – only abstract available. |
| **Wilson et al., 2015 [5]** | No full-text available – only abstract available. |

1. Adedoyin ACA. A systematic review of evidence-based cancer education media interventions to improve cancer screening behaviors among African Americans in the United States. Dissertation Abstracts International Section A: Humanities and Social Sciences. 2014;75(6-A(E)).
2. Fernandez M. Perceptions of the HPV vaccine among U.S. Hispanic females: A theory-guided systematic review. Sex Transm Dis. 2016:S161.
3. Kiekenbush C. The human papillomavirus vaccine: A systematic review elucidating the dynamics of ethical concerns and public health policies. Journal of the American Society of Cytopathology. 2012.
4. Kuk N, To J, McBride C, Hong A, Ng E, Li N, et al. Systematic review: Does a positive human papillomavirus vaccination status increase the risk of unsafe sexual health practice in Australian women? Sex Transm Infect. 2015.
5. Wilson AR, Hashibe M, Gren LH, Chou C-C, Gardener AT, Greenwood J, et al. Factors predicting human papillomavirus vaccination intention and uptake: A meta-analysis and systematic review. Pharmacoepidemiol Drug Saf. 2015.
