# Supplementary material for: Communication around HPV vaccination for adolescents in low- and middle-income countries: a systematic scoping overview of systematic reviews
Source: Syst Rev. 2019 Aug 1;8:190. doi: 10.1186/s13643-019-1100-y (PMC6670236; doi:10.1186/s13643-019-1100-y)
Supplement: Supplementary file 7 — Table of funding and conflicts of interest as reported in the reviews. (PDF 38 kb) [file 13643_2019_1100_MOESM7_ESM.pdf]

Additional file 7: Table of funding and conflicts of interest as reported in the reviews

Review	Funding	Conflicts of interest
**Abdullahi et al., 2016 [1]**	None	None
**Badawy et al., 2017 [2][2]**	None	None
**Ferrer et al., 2014 [3]**	The work was supported by the Centre for the Development and Evaluation of Complex Interventions for Public Health Improvement (DECIPHer) which receives funding from the British Heart Foundation, Cancer Research UK, Economic and Social Research Council (RES-590-28-0005), Medical Research Council, the Welsh Government and the Wellcome Trust (WT087640MA), under the auspices of the UK Clinical Research Collaboration	None
**Fu et al., 2014 [4]**	This work was funded in part by the Eunice Kennedy Shriver National Institute of Child Health & Human Development grant no. 1K23HD068394-01A1 (L.Y.F.)	Dr. Fu has served on advisory boards on reducing barriers to immunization and review committees for Independent Grants for Learning & Change for Pfizer, Inc. She has also received an investigator-initiated, unrestricted research grant from Pfizer, Inc;<br>Ms. Bonhomme has no disclosures to report;<br>Dr. Robbins has received an unrestricted research grant from CSL Biotherapies;<br>Dr. Joseph has no disclosures to report;<br>Dr. Zimet has been a co-investigator on investigator-initiated HPV-related research projects funded by Merck and has received an unrestricted program development grant from GlaxoSmithKline
**Hendry et al., 2013 [5]**	This review is part of the multi-method HPV Core Messages project, funded by Cancer Research UK (Ref. C1273/A9479)	No information
**Johnson et al., 2018 [6]**	At the time of this review, LJ was supported with a Hillman Scholars Program in Nursing Innovation Predoctoral Fellowship, National Cancer Institute Diversity Supplement (U54CA190158—Parent Grant), and American Cancer Society Doctoral Degree Scholarship in Cancer Nursing (DSCN-16-112-01)	None
**Kang et al., 2018 [7]**	None	No information
**Kim et al., 2017 [8]**	None	No information
**Newman et al., 2013 [9]**	This research was funded in part by grants from the Canadian Institutes of Health Research (funding reference number THA-118570) through the Canadian HIV Vaccine	None

	Initiative, and the Canada Research Chairs program (950-204522)	
**Newman et al,. 2018 [10]**	This work was supported in part by the Canadian Institutes of Health Research (THA-118570), the Canada Research Chairs Program (Canada Research Chair in Health and Social Justice) and the Canada Foundation for Innovation	None
**Radisic et al., 2017 [11]**	None	None
**Rambout et al., 2014 [12]**	All authors have completed the Conflict of Interest Policy available on request from the corresponding author and declare that: (1) LR, MT, LH, & ACT did not receive any financial support for the submitted work; (2) LR, MT, & LH have no relationships with any companies that might have an interest in the submitted work in the previous 3 years, ACT was a consultant for GlaxoSmithKline Canada vaccine products, including their human papillomavirus vaccine from December 2003 until September 2011; (3) their spouses, partners, or children have no financial relationships that may be relevant to the submitted work; and (4) LR, MT, LH, & ACT have no non-financial interests that may be relevant to the sub- mitted work	Completed Conflict of Interest Policy available on request from the corresponding author

1. Abdullahi LH, Kagina BM, Cassidy T, Adebayo EF, Wiysonge CS, Hussey GD. Knowledge, attitudes and practices on adolescent vaccination among adolescents, parents and teachers in Africa: A systematic review. Vaccine. 2016;34(34):3950-60.
2. Badawy SM, Kuhns LM. Texting and Mobile Phone App Interventions for Improving Adherence to Preventive Behavior in Adolescents: A Systematic Review. JMIR mHealth and uHealth. 2017;5(4):e50.
3. Ferrer HB, Trotter C, Hickman M, Audrey S. Barriers and facilitators to HPV vaccination of young women in high-income countries: a qualitative systematic review and evidence synthesis. BMC Public Health. 2014;14:700.
4. Fu LY, Bonhomme LA, Cooper SC, Joseph JG, Zimet GD. Educational interventions to increase HPV vaccination acceptance: a systematic review. Vaccine. 2014;32(17):1901-20.
5. Hendry M, Lewis R, Clements A, Damery S, Wilkinson C. "HPV? Never heard of it!": a systematic review of girls' and parents' information needs, views and preferences about human papillomavirus vaccination. Vaccine. 2013;31(45):5152-67.
6. Johnson LG, Armstrong A, Joyce CM, Teitelman AM, Buttenheim AM. Implementation strategies to improve cervical cancer prevention in sub-Saharan Africa: a systematic review. Implementation science : IS. 2018;13(1):28.
7. Kang HS, De Gagne JC, Son YD, Chae S-M. Completeness of Human Papilloma Virus Vaccination: A Systematic Review. J Pediatr Nurs. 2018;39:7-14.
8. Kim K, LeClaire AR. A systematic review of factors influencing human papillomavirus vaccination among immigrant parents in the United States. Health Care Women Int. 2017:0.
9. Newman PA, Logie CH, Doukas N, Asakura K. HPV vaccine acceptability among men: a systematic review and meta-analysis. Sex Transm Infect. 2013;89(7):568-74.

10. Newman PA, Logie CH, Lacombe-Duncan A, Baiden P, Tepjan S, Rubincam C, Doukas N, Asey F. Parents' uptake of human papillomavirus vaccines for their children: a systematic review and meta-analysis of observational studies. BMJ open. 2018;8(4):e019206.
11. Radisic G, Chapman J, Flight I, Wilson C. Factors associated with parents' attitudes to the HPV vaccination of their adolescent sons : A systematic review. Prev Med. 2017;95:26-37.
12. Rambout L, Tashkandi M, Hopkins L, Tricco AC. Self-reported barriers and facilitators to preventive human papillomavirus vaccination among adolescent girls and young women: a systematic review. Prev Med. 2014;58(1):22-32.
