# Supplementary material for: Communication around HPV vaccination for adolescents in low- and middle-income countries: a systematic scoping overview of systematic reviews
Source: Syst Rev. 2019 Aug 1;8:190. doi: 10.1186/s13643-019-1100-y (PMC6670236; doi:10.1186/s13643-019-1100-y)
Supplement: Supplementary file 8 — Summary of the results of each review, in relation to the scoping overview objectives. (PDF 59 kb) [file 13643_2019_1100_MOESM8_ESM.pdf]

Additional file 8: Summary of the results of each review, in relation to the scoping overview objectives

Review	Aims as stated in the review	Aims in relation to overview objectives	Results as expressed by the review authors, in relation to overview objectives
**Badawy et al., 2016 [1][1]**	Evaluate efficacy of text messaging and mobile phone app interventions to improve adherence to preventive behavior, describe intervention approaches	Efficacy of text messaging and mobile phone app interventions, describe intervention approaches	"In conclusion, despite the promising feasibility and acceptability data of texting and mobile phone apps in improving preventive behavior among adolescents, the evidence for actual behavior change is modest, with most studies of relatively low to moderate quality."
**Fu et al., 2014 [2]**	Focus on educational interventions designed to increase HPV vaccine acceptance	Educational interventions	"Studies of educational interventions to increase HPV vaccination uptake have to review's date largely focused on written informational handouts targeted toward educated populations. There is not strong evidence to recommend any specific educational intervention for wide-spread implementation."
**Johnson et al., 2018 [3]**	Uncover breadth and diversity of implementation strategies used to improve the uptake and sustainability of cervical cancer prevention programs	Implementation strategies	"This systematic review elicits the need to diversify strategies that are used to improve implementation for cervical cancer prevention programs. While education is important, implementation science literature reveals that dissemination of information in isolation is not as effective in generating change."
**Kang et al., 2018 [4]**	Evaluate impact of interventions implemented after the first dose of HPV vaccination on the rate of HPV vaccine completion	Interventions after vaccination initiation	"A comprehensive systematic review of the literature found few studies on intervention for HPV vaccine completeness. This review found evidence that interventions, such as reminders, contributed to the promotion of HPV vaccination completion. However, the overall completeness rates of HPV vaccination remained low. This review shows that establishing a reminder system and actively reaching out to all target populations that are recommended to receive the HPV vaccine, including children from age 9, adolescents, and women up to 26 are important at the health center level. At the personal level, women who receive messages about their vaccinations are more likely to place a high priority on their health and get vaccinated on time."
**Abdullahi et al., 2016 [5]**	Knowledge, attitudes and practices among stakeholders	Attitudes	"There are two main themes that emerged in this review. Firstly, there are high levels of acceptability and willingness to vaccinate adolescents against HPV by all the three key groups: adolescents, teachers and parents. This holds true even when knowledge levels are low. Secondly, it is evident in the review that participants are in need for an increased

			education and awareness of adolescents' vaccination among all the three key groups. Although attitudes were generally positive, lack of optimal knowledge may lead to misconceptions which in turn hinders vaccine uptake."
**Hendry et al., 2013 [6]**	Information needs, views and preferences	Views and preferences	"Overall the acceptability of HPV vaccination was high. However, people had insufficient knowledge and understanding about HPV vaccination and a poor grasp of the facts was revealed, with participants struggling to interpret limited information in the context of existing knowledge, impacting on the ability to make informed choices. This lack of understanding permeated all our findings.
**Ferrer et al., 2014 [7]**	Facilitators and barriers to decision-making by key stakeholders	Facilitators and barriers associated with decision-making	In healthcare settings, an important prompt to uptake appeared to be the decision for the healthcare professional to recommend vaccination. This has been reported in a systematic review, comprising predominantly quantitative primary studies, of barriers to HPV vaccination and a statistical analysis linking HPV vaccine uptake with survey data from the US-Teen Survey. The qualitative data also suggest the decision to recommend was influenced by concerns about safety of the HPV vaccine. However, value judgements about a young women's likely sexual activity were also influential."
**Kim et al., 2017 [8]**	Awareness, intention, and uptake among immigrant parents	Intention and uptake	"This review found that significant correlates of HPV vaccination comprise intrapersonal factors such as awareness and knowledge of HPV vaccine, concerns over vaccine safety and effectiveness, and sexual promiscuity; interpersonal factors, including providers' recommendation; and community and public policy factors, including misunderstanding of immigration laws and school requirements."
**Newman et al., 2013 [9]**	Acceptability and factors correlated with acceptability	Factors associated with acceptability	"A moderate level of human papillomavirus (HPV) vaccine acceptability (50.4 on a 100-point scale) was reported among 8360 men across 22 studies.<br><br>Perceived HPV vaccine benefits and healthcare provider recommendation were the two most influential correlates of HPV vaccine acceptability among men.<br><br>HPV vaccine cost and logistical barriers may pose significant obstacles to uptake.<br><br>HPV vaccination campaigns targeting men should promote awareness of HPV, HPV-associated cancer risks and HPV vaccine efficacy, and healthcare providers' recommendation of HPV vaccination for boys".

**Newman et al., 2018 [10]**	Parents' uptake, examine factors correlated with parents' uptake, possible moderating influences of sex of child and parent on uptake	Uptake and factors associated with uptake	"In line with previous descriptive reviews largely focused on uptake for girls, physician recommendation had the single greatest effect on parents' uptake of HPV vaccines for their children, supported by evidence from over 20 studies. As the first meta-analysis of HPV vaccine uptake, to our knowledge, to test for the moderating influence of child's sex—and based on previous studies that suggest a tendency on the part of healthcare providers to offer HPV vaccine recommendations to those they perceive to be more likely to benefit from and to accept vaccination (ie, girls, patients with health insurance)—this highlights the importance of physicians making recommendations for boys as well as girls in order to increase parents' HPV vaccine uptake for their children."
**Radisic et al., 2016 [11]**	Factors associated with acceptability in parents of adolescent boys	Factors associated with acceptability	"The majority of studies (n = 15) gave an indication of the level of knowledge of HPV or the HPV vaccine in their sample using a variety of methods, but only 10 directly reported findings of the association between knowledge and the vaccine acceptability outcome.<br>Of the 18 studies, 10 studies (56%) asked about the perceived risk of adolescent boys acquiring a disease and whether it would influence the decision to vaccinate them. Parents in six studies believed their sons to be at risk of contracting the HPV infection and this was positively associated with intention to vaccinate. Twelve studies (67%) reported a link between the perceived benefits of HPV vaccination and HPV vaccination acceptance. Perceived barriers were associated with non-acceptability of the vaccine. One of the most common barriers included fear of side effects and concern about vaccine safety. "
**Rambout et al., 2013 [12]**	Barriers and facilitators to vaccination	For quantitative studies: Barriers and facilitators associated with intention or uptake. For qualitative studies: Reported barriers and facilitators to uptake.	"We have found that young women up to the age of 26 often feel that they do not need the HPV vaccine and that they view cost as an important barrier to vaccination. Safety and side effects are additional concerns. We found that young women are most encouraged to seek vaccination if they perceive a benefit to vaccination. And, while endorsement by influential others, such as parents and health care providers, appears to be important factors in prompting vaccination, communication can some- times be suboptimal or unclear. Furthermore, we found that adolescent girls do not perceive a need to get vaccinated until becoming sexually active. They commonly fear needles, want more information about vaccine safety and efficacy, and consider parental approval important. Our results should be

	interpreted and applied cautiously, due to the limitations identified in the included studies.”

1 Only includes one study related to HPV vaccination

1. Badawy SM, Kuhns LM. Texting and Mobile Phone App Interventions for Improving Adherence to Preventive Behavior in Adolescents: A Systematic Review. JMIR mHealth and uHealth. 2017;5(4):e50.
2. Fu LY, Bonhomme LA, Cooper SC, Joseph JG, Zimet GD. Educational interventions to increase HPV vaccination acceptance: a systematic review. Vaccine. 2014;32(17):1901-20.
3. Johnson LG, Armstrong A, Joyce CM, Teitelman AM, Buttenheim AM. Implementation strategies to improve cervical cancer prevention in sub-Saharan Africa: a systematic review. Implementation science : IS. 2018;13(1):28.
4. Kang HS, De Gagne JC, Son YD, Chae S-M. Completeness of Human Papilloma Virus Vaccination: A Systematic Review. J Pediatr Nurs. 2018;39:7-14.
5. Abdullahi LH, Kagina BM, Cassidy T, Adebayo EF, Wiysonge CS, Hussey GD. Knowledge, attitudes and practices on adolescent vaccination among adolescents, parents and teachers in Africa: A systematic review. Vaccine. 2016;34(34):3950-60.
6. Hendry M, Lewis R, Clements A, Damery S, Wilkinson C. "HPV? Never heard of it!": a systematic review of girls' and parents' information needs, views and preferences about human papillomavirus vaccination. Vaccine. 2013;31(45):5152-67.
7. Ferrer HB, Trotter C, Hickman M, Audrey S. Barriers and facilitators to HPV vaccination of young women in high-income countries: a qualitative systematic review and evidence synthesis. BMC Public Health. 2014;14:700.
8. Kim K, LeClaire AR. A systematic review of factors influencing human papillomavirus vaccination among immigrant parents in the United States. Health Care Women Int. 2017:0.
9. Newman PA, Logie CH, Doukas N, Asakura K. HPV vaccine acceptability among men: a systematic review and meta-analysis. Sex Transm Infect. 2013;89(7):568-74.
10. Newman PA, Logie CH, Lacombe-Duncan A, Baiden P, Tepjan S, Rubincam C, et al. Parents' uptake of human papillomavirus vaccines for their children: a systematic review and meta-analysis of observational studies. BMJ open. 2018;8(4):e019206.
11. Radisic G, Chapman J, Flight I, Wilson C. Factors associated with parents' attitudes to the HPV vaccination of their adolescent sons : A systematic review. Prev Med. 2017;95:26-37.
12. Rambout L, Tashkandi M, Hopkins L, Tricco AC. Self-reported barriers and facilitators to preventive human papillomavirus vaccination among adolescent girls and young women: a systematic review. Prev Med. 2014;58(1):22-32.
