# Supplementary material for: Communication around HPV vaccination for adolescents in low- and middle-income countries: a systematic scoping overview of systematic reviews
Source: Syst Rev. 2019 Aug 1;8:190. doi: 10.1186/s13643-019-1100-y (PMC6670236; doi:10.1186/s13643-019-1100-y)
Supplement: Supplementary file 9 — Applicability of the evidence to LMICs, with explanations of assessments. (PDF 56 kb) [file 13643_2019_1100_MOESM9_ESM.pdf]

Additional file 9: Applicability of the evidence to LMICs, with explanations of assessments

Source	Were some or all of the studies included in the systematic review conducted in LMICs or were the findings in the review consistent across settings or time periods and therefore suggest wide applicability? [Proportion of studies conducted in LMICs]	Are there important differences in on-the-ground realities and constraints in LMICs that might substantially alter the feasibility and acceptability of the intervention (where applicable) or raise questions about the applicability of the review findings?	Reasoning and notes related to previous question	Are there important differences in health system arrangements that may mean an intervention could not work in the same way in LMICs or review findings may not be applicable to LMICs?	Reasoning and notes related to previous question
**Abdullahi et al., 2016 [1]**	Yes [18/18]	Likely no	This review focused specifically on African settings. However, judgement would be required regarding the applicability of the findings to LMIC settings outside of Africa	Largely not applicable	Most of the studies included in the review did not evaluate interventions
**Badawy et al., 2017 [2]**	No[1]	Likely yes	The review included only one study on HPV and this study was conducted in the USA	Yes	The one HPV study included in the review was conducted in an urban paediatric clinic in the USA, a setting with very different health systems arrangements to most LMICs
**Ferrer et al., 2014 [3][3]**	No	Likely yes	Although the review included studies from HICs only, some of the cross-cutting themes may be broadly applicable to LMICs	Likely yes	Many findings seem relevant to LMICs. However, other factors related to the costs of vaccination may play out differently in many LMIC settings depending on how vaccination is funded
**Fu et al., 2014 [4][3]**	Yes [2/33]	Likely yes	Many of the interventions were based on written information and required participants to be literate. Differences in levels of literacy may therefore impact on the applicability of these findings	Likely yes	Although the health systems settings were varied, most studies were undertaken in HICs
**Hendry et al., 2013 [5]**	Yes [11/72]	Likely no	Included a fair number of studies form LMICs and the results seem transferrable	Likely no	People's views on associated factors are probably not closely linked to health system arrangements
**Johnson et al., 2018 [6]**	Yes [53/53]	Likely no	This review focused specifically on African settings. However, judgement would be required regarding the applicability of the findings to LMIC settings outside of Africa	Likely no	[2]

**Kang et al., 2018 [7]**	No	Likely yes	Many of the included studies used digital intervention strategies, such as email or internet messaging, that may be not be available to many populations in LMICs	Likely yes	Digital messaging systems require population registries that may not be available or very complete in many LMICs
**Kim et al., 2017 [8]**	No	Likely no	Although the review only included studies from the USA, these focused on immigrant populations and many of the issues identified may therefore be applicable in LMICs	Likely no	See reasoning in column 4
**Newman et al., 2013 [9]**	Yes [2/29]	Likely yes	The review included few LMIC studies and it is unclear if the results are broadly applicable to men in LMICs, particularly as HPV vaccination is not offered routinely to men in most LMICs	Likely yes	HPV vaccination is not offered routinely to men in most LMICs. If it were to be offered, health systems factors such as cost and access are likely to be different from HIC settings
**Newman et al,. 2018 [10]**	Yes [6/79]	Likely no	The review included a number of studies from LMICs. However differences in findings between LMIC and HIC settings were not examined	Likely no	The key factors identified in the review as affecting HPV uptake, such as safety concerns, may be applicable across settings. However, other factors related to the costs of vaccination may play out differently in many LMIC settings depending on how vaccination is funded
**Radisic et al., 2017 [11]**	No	Yes	The review did not include any studies from LMICs and it is unclear if the results are broadly applicable to men in these settings, particularly as HPV vaccination is not offered routinely to men in most LMICs	Likely yes	HPV vaccination is not offered routinely to men in most LMICs. If it were to be offered, health systems factors such as cost and access are likely to be different from HIC settings
**Rambout et al., 2014 [12][3]**	No	Likely yes	The review did not included any studies from LMICs and it is unclear if the results are broadly applicable to these settings	Likely yes	The dominant factor identified in the review as affecting HPV uptake – the costs of vaccination – may play out differently in many LMIC settings, depending on how vaccination is funded

1 The review includes one study from a LMIC, however this study does not focus on HPV
2 Difficult to assess due to superficial reporting of the results. In addition, many of the included studies did not focus on HPV vaccination specifically
3 Did not intend to include studies from LMICs

1. Abdullahi LH, Kagina BM, Cassidy T, Adebayo EF, Wiysonge CS, Hussey GD. Knowledge, attitudes and practices on adolescent vaccination among adolescents, parents and teachers in Africa: A systematic review. Vaccine. 2016;34(34):3950-60.

2. Badawy SM, Kuhns LM. Texting and Mobile Phone App Interventions for Improving Adherence to Preventive Behavior in Adolescents: A Systematic Review. JMIR mHealth and uHealth. 2017;5(4):e50.

3. Ferrer HB, Trotter C, Hickman M, Audrey S. Barriers and facilitators to HPV vaccination of young women in high-income countries: a qualitative systematic review and evidence synthesis. BMC Public Health. 2014;14:700.

4. Fu LY, Bonhomme LA, Cooper SC, Joseph JG, Zimet GD. Educational interventions to increase HPV vaccination acceptance: a systematic review. Vaccine. 2014;32(17):1901-20.

5. Hendry M, Lewis R, Clements A, Damery S, Wilkinson C. "HPV? Never heard of it!": a systematic review of girls' and parents' information needs, views and preferences about human papillomavirus vaccination. Vaccine. 2013;31(45):5152-67.

6. Johnson LG, Armstrong A, Joyce CM, Teitelman AM, Buttenheim AM. Implementation strategies to improve cervical cancer prevention in sub-Saharan Africa: a systematic review. Implementation science : IS. 2018;13(1):28.

7. Kang HS, De Gagne JC, Son YD, Chae S-M. Completeness of Human Papilloma Virus Vaccination: A Systematic Review. J Pediatr Nurs. 2018;39:7-14.

8. Kim K, LeClaire AR. A systematic review of factors influencing human papillomavirus vaccination among immigrant parents in the United States. Health Care Women Int. 2017:0.

9. Newman PA, Logie CH, Doukas N, Asakura K. HPV vaccine acceptability among men: a systematic review and meta-analysis. Sex Transm Infect. 2013;89(7):568-74.

10. Newman PA, Logie CH, Lacombe-Duncan A, Baiden P, Tepjan S, Rubincam C, et al. Parents' uptake of human papillomavirus vaccines for their children: a systematic review and meta-analysis of observational studies. BMJ open. 2018;8(4):e019206.

11. Radisic G, Chapman J, Flight I, Wilson C. Factors associated with parents' attitudes to the HPV vaccination of their adolescent sons : A systematic review. Prev Med. 2017;95:26-37.

12. Rambout L, Tashkandi M, Hopkins L, Tricco AC. Self-reported barriers and facilitators to preventive human papillomavirus vaccination among adolescent girls and young women: a systematic review. Prev Med. 2014;58(1):22-32.
