# Supplementary material for: Communication around HPV vaccination for adolescents in low- and middle-income countries: a systematic scoping overview of systematic reviews
Source: Syst Rev. 2019 Aug 1;8:190. doi: 10.1186/s13643-019-1100-y (PMC6670236; doi:10.1186/s13643-019-1100-y)
Supplement: Supplementary file 10 — Individual AMSTAR 2 assessments. (PDF 770 kb) [file 13643_2019_1100_MOESM10_ESM.pdf]

Additional file 10: Individual AMSTAR 2 assessments

Criteria for assessing reviews (adapted from AMSTAR)

Review author, year: Abdullahi et al., 2016.

Criterion	Assessment	Comments
1. Did the review have a clear research question and inclusion criteria?	Yes	
2. Did the report of the review contain an explicit statement that the review methods were established prior to the conduct of the review and did the report justify any significant deviations from the protocol?	Yes	https://www.crd.york.ac.uk/PROSPERO/display_record.php?RecordID=10395<br>No significant deviations from the protocol found.
3. Did the review authors explain their selection of the study designs for inclusion in the review?	No	Included study designs thoroughly specified, but not the reasoning. Minor limitation.
4. Did the review authors use a comprehensive literature search strategy?<br><br>*For Partial Yes (all the following):*<br>☐ **searched** at least 2 databases (relevant to research question)<br>☐ **provided** key word and/or search strategy<br>☐ **justified** publication restrictions (e.g. language)<br><br>*For Yes, should also have (all the following):*<br>☐ **searched** the reference lists / bibliographies of included studies<br>☐ included/consulted content experts in the field<br>☐ **where** relevant, searched for grey literature	☐ Yes<br>☐ **Partial** Yes<br>☐ No	Did not consult experts in the field. Minor limitation.

☐ **conducted** search within 24 months of completion of the review		

5. Did the review authors perform study selection in duplicate?	Yes	
6. Did the review authors perform data extraction in duplicate?	Yes	
7. Did the review authors provide a list of excluded studies and justify the exclusions?<br><br>*For Partial Yes:*<br>☐ provided a list of all potentially relevant studies that were read in full-text form but excluded from the review<br><br>*For Yes, must also have:*<br>☐ Justified the exclusion from the review of each potentially relevant study	☐ **Yes**<br>☐ Partial Yes<br>☐ No	
8. Did the review authors describe the included studies in adequate detail?	Yes	
9. Did the review authors use a satisfactory technique for assessing the methodological limitations / risk of bias (RoB) in individual studies that were included in the review?	Yes	The assessment of methodological limitations is not explained per individual study, but is used and explained overall.
10. Did the review authors report on the sources of funding for the studies included in the review?	No	Minor limitation.
11. If a synthesis was performed did the review authors use appropriate methods to combine the results of individual studies?	Yes	
12. If synthesis was performed, did the review authors assess the potential impact of the methodological limitations / RoB in individual studies on the results of the evidence synthesis?	Yes	
13. Did the review authors account for methodological limitations / RoB in individual studies when interpreting/ discussing the results of the review?	Yes	

14. Did the review authors provide a satisfactory explanation for, and discussion of, any heterogeneity observed in the results of the review?<br><br>(*Omit for QES and for some reviews of survey data*)	No	Minor limitation.
15. If they performed quantitative synthesis did the review authors carry out an adequate investigation of publication bias (small study bias) and discuss its likely impact on the results of the review?<br><br>(*Omit for QES*)	Yes	
16. Did the review authors report any potential sources of conflict of interest, including any funding they received for conducting the review?	Yes	No conflict of interest. No funding received.

**Overall assessment**	**Minor limitations.**
**Explanation of overall assessment**	**Four minor limitations.**

Criteria for assessing reviews (adapted from AMSTAR)

Review author, year: Allen et al., 2010.

Criterion	Assessment	Comments
1. Did the review have a clear research question and inclusion criteria?	Yes	
2. Did the report of the review contain an explicit statement that the review methods were established prior to the conduct of the review and did the report justify any significant deviations from the protocol?	No	Not found on Prospero or BMC Systematic Reviews. Minor limitation.
3. Did the review authors explain their selection of the study designs for inclusion in the review?	No	Minor limitation.
4. Did the review authors use a comprehensive literature search strategy?<br><br>*For Partial Yes (all the following):*<br>☐ **searched** at least 2 databases (relevant to research question)<br>☐ **provided** key word and/or search strategy<br>☐ justified publication restrictions (e.g. language)<br><br>*For Yes, should also have (all the following):*<br>☐ searched the reference lists / bibliographies of included studies<br>☐ included/consulted content experts in the field<br>☐ where relevant, searched for grey literature<br>☐ conducted search within 24 months of completion of the review	☐ Yes<br>☐ Partial Yes<br>☐ **No**	Did not justify language restriction to English. Minor limitation.

5. Did the review authors perform study selection in duplicate?	No	Minor limitation.
6. Did the review authors perform data extraction in duplicate?	Yes	
7. Did the review authors provide a list of excluded studies and justify the exclusions?<br><br>*For Partial Yes:*<br>☐ provided a list of all potentially relevant studies that were read in full-text form but excluded from the review<br><br>*For Yes, must also have:*<br>☐ Justified the exclusion from the review of each potentially relevant study	☐ Yes<br>☐ Partial Yes<br>☐ **No**	Minor limitation.
8. Did the review authors describe the included studies in adequate detail?	Yes	
9. Did the review authors use a satisfactory technique for assessing the methodological limitations / risk of bias (RoB) in individual studies that were included in the review?	No	Minor limitation.
10. Did the review authors report on the sources of funding for the studies included in the review?	No	Minor limitation.
11. If a synthesis was performed did the review authors use appropriate methods to combine the results of individual studies?	Yes	
12. If synthesis was performed, did the review authors assess the potential impact of the methodological limitations / RoB in individual studies on the results of the evidence synthesis?	No	Minor limitation.
13. Did the review authors account for methodological limitations / RoB in individual studies when interpreting/ discussing the results of the review?	No	Minor limitation.

14. Did the review authors provide a satisfactory explanation for, and discussion of, any heterogeneity observed in the results of the review?<br><br>(*Omit for QES and for some reviews of survey data*)	No	Minor limitation.
15. If they performed quantitative synthesis did the review authors carry out an adequate investigation of publication bias (small study bias) and discuss its likely impact on the results of the review?<br><br>(*Omit for QES*)	No	Minor limitation.
16. Did the review authors report any potential sources of conflict of interest, including any funding they received for conducting the review?	No	Minor limitation.

**Overall assessment**	**Important limitations.**
**Explanation of overall assessment**	**Twelve minor limitations.**

Criteria for assessing reviews (adapted from AMSTAR)

Review author, year: Badawy et al., 2017.

Criterion	Assessment	Comments
1. Did the review have a clear research question and inclusion criteria?	Yes	
2. Did the report of the review contain an explicit statement that the review methods were established prior to the conduct of the review and did the report justify any significant deviations from the protocol?	Yes	
3. Did the review authors explain their selection of the study designs for inclusion in the review?	Yes	No selection.
4. Did the review authors use a comprehensive literature search strategy?<br><br>*For Partial Yes (all the following):*<br>☐ **searched** at least 2 databases (relevant to research question)<br>☐ **provided** key word and/or search strategy<br>☐ justified publication restrictions (e.g. language)<br><br>*For Yes, should also have (all the following):*<br>☐ searched the reference lists / bibliographies of included studies<br>☐ included/consulted content experts in the field<br>☐ where relevant, searched for grey literature<br>☐ **conducted** search within 24 months of completion of the review	☐ Yes<br>☐ **Partial** Yes<br>☐ No	No publication restrictions. Minor limitation.

5. Did the review authors perform study selection in duplicate?	Yes	
6. Did the review authors perform data extraction in duplicate?	Yes	
7. Did the review authors provide a list of excluded studies and justify the exclusions?<br><br>*For Partial Yes:*<br>☐ provided a list of all potentially relevant studies that were read in full-text form but excluded from the review<br><br>*For Yes, must also have:*<br>☐ Justified the exclusion from the review of each potentially relevant study	☐ Yes<br>☐ Partial Yes<br>☐ **No**	Minor limitation.
8. Did the review authors describe the included studies in adequate detail?	Yes	
9. Did the review authors use a satisfactory technique for assessing the methodological limitations / risk of bias (RoB) in individual studies that were included in the review?	No	Major limitation.
10. Did the review authors report on the sources of funding for the studies included in the review?	No	Minor limitation.
11. If a synthesis was performed did the review authors use appropriate methods to combine the results of individual studies?	No	Minor limitation.
12. If synthesis was performed, did the review authors assess the potential impact of the methodological limitations / RoB in individual studies on the results of the evidence synthesis?	No	Minor limitation.
13. Did the review authors account for methodological limitations / RoB in individual studies when interpreting/ discussing the results of the review?	Yes	

14. Did the review authors provide a satisfactory explanation for, and discussion of, any heterogeneity observed in the results of the review?<br><br>(*Omit for QES and for some reviews of survey data*)	No	Minor limitation.
15. If they performed quantitative synthesis did the review authors carry out an adequate investigation of publication bias (small study bias) and discuss its likely impact on the results of the review?<br><br>(*Omit for QES*)	Yes	
16. Did the review authors report any potential sources of conflict of interest, including any funding they received for conducting the review?	Yes	None declared.

**Overall assessment**	**Minor limitations.**
**Explanation of overall assessment**	**One major limitation. Six minor limitations.**

Criteria for assessing reviews (adapted from AMSTAR)

Review author, year: Brewer et al., 2007.

Criterion	Assessment	Comments
1. Did the review have a clear research question and inclusion criteria?	Yes	
2. Did the report of the review contain an explicit statement that the review methods were established prior to the conduct of the review and did the report justify any significant deviations from the protocol?	No	Minor limitation.
3. Did the review authors explain their selection of the study designs for inclusion in the review?	No	Minor limitation.
4. Did the review authors use a comprehensive literature search strategy?<br><br>*For Partial Yes (all the following):*<br>☐ **searched** at least 2 databases (relevant to research question)<br>☐ **provided** key word and/or search strategy<br>☐ **justified** publication restrictions (e.g. language)<br><br>*For Yes, should also have (all the following):*<br>☐ **searched** the reference lists / bibliographies of included studies<br>☐ included/consulted content experts in the field<br>☐ where relevant, searched for grey literature<br>☐ conducted search within 24 months of completion of the review	☐ Yes<br>☐ **Partial** Yes<br>☐ No	Minor limitation.

5. Did the review authors perform study selection in duplicate?	Yes	
6. Did the review authors perform data extraction in duplicate?	No	Minor limitation.
7. Did the review authors provide a list of excluded studies and justify the exclusions?<br><br>*For Partial Yes:*<br>☐ provided a list of all potentially relevant studies that were read in full-text form but excluded from the review<br><br>*For Yes, must also have:*<br>☐ Justified the exclusion from the review of each potentially relevant study	☐ Yes<br>☐ Partial Yes<br>☐ **No**	Minor limitation.
8. Did the review authors describe the included studies in adequate detail?	Yes	
9. Did the review authors use a satisfactory technique for assessing the methodological limitations / risk of bias (RoB) in individual studies that were included in the review?	No	Major limitation.
10. Did the review authors report on the sources of funding for the studies included in the review?	No	Minor limitation.
11. If a synthesis was performed did the review authors use appropriate methods to combine the results of individual studies?	Yes	
12. If synthesis was performed, did the review authors assess the potential impact of the methodological limitations / RoB in individual studies on the results of the evidence synthesis?	No	Minor limitation.
13. Did the review authors account for methodological limitations / RoB in individual studies when interpreting/ discussing the results of the review?	No	Minor limitation.

14. Did the review authors provide a satisfactory explanation for, and discussion of, any heterogeneity observed in the results of the review?<br><br>(*Omit for QES and for some reviews of survey data*)	No	Minor limitation.
15. If they performed quantitative synthesis did the review authors carry out an adequate investigation of publication bias (small study bias) and discuss its likely impact on the results of the review?<br><br>(*Omit for QES*)	No	Minor limitation.
16. Did the review authors report any potential sources of conflict of interest, including any funding they received for conducting the review?	Yes	

**Overall assessment**	**Important limitations.**
**Explanation of overall assessment**	**One major limitation. Ten minor limitations.**

Criteria for assessing reviews (adapted from AMSTAR)

Review author, year: Catalan-Matamoros et al., 2017.

Criterion	Assessment	Comments
1. Did the review have a clear research question and inclusion criteria?	Yes	
2. Did the report of the review contain an explicit statement that the review methods were established prior to the conduct of the review and did the report justify any significant deviations from the protocol?	Yes	https://www.crd.york.ac.uk/PROSPERO/display_record.php?RecordID=72849
3. Did the review authors explain their selection of the study designs for inclusion in the review?	Yes	No selection of study design.
4. Did the review authors use a comprehensive literature search strategy?<br><br>*For Partial Yes (all the following):*<br>☐ **searched** at least 2 databases (relevant to research question)<br>☐ **provided** key word and/or search strategy<br>☐ **justified** publication restrictions (e.g. language)<br><br>*For Yes, should also have (all the following):*<br>☐ **searched** the reference lists / bibliographies of included studies<br>☐ included/consulted content experts in the field<br>☐ where relevant, searched for grey literature	☐ Yes<br>☐ **Partial** Yes<br>☐ No	Publication restriction of time explained, but not language. Minor limitation.

☐ **conducted** search within 24 months of completion of the review		

5. Did the review authors perform study selection in duplicate?	No	Not specified. Minor limitation.
6. Did the review authors perform data extraction in duplicate?	No	Coding form developed by review team, but studies only reviewed by one author. Minor limitation.
7. Did the review authors provide a list of excluded studies and justify the exclusions?<br><br>*For Partial Yes:*<br>☐ provided a list of all potentially relevant studies that were read in full-text form but excluded from the review<br><br>*For Yes, must also have:*<br>☐ Justified the exclusion from the review of each potentially relevant study	☐ Yes<br>☐ Partial Yes<br>☐ **No**	Reasons included, but not for individual studies. Minor limitation.
8. Did the review authors describe the included studies in adequate detail?	Yes	In two supplementary tables.
9. Did the review authors use a satisfactory technique for assessing the methodological limitations / risk of bias (RoB) in individual studies that were included in the review?	No	Major limitation.
10. Did the review authors report on the sources of funding for the studies included in the review?	No	Minor limitation.
11. If a synthesis was performed did the review authors use appropriate methods to combine the results of individual studies?	Yes	
12. If synthesis was performed, did the review authors assess the potential impact of the methodological limitations / RoB in individual studies on the results of the evidence synthesis?	No	Minor limitation.
13. Did the review authors account for methodological limitations / RoB in individual studies when interpreting/ discussing the results of the review?	No	Mentioned that RoB evaluation could be done, but not accounted for. Minor limitation.

14. Did the review authors provide a satisfactory explanation for, and discussion of, any heterogeneity observed in the results of the review?<br><br>(*Omit for QES and for some reviews of survey data*)	Yes	
15. If they performed quantitative synthesis did the review authors carry out an adequate investigation of publication bias (small study bias) and discuss its likely impact on the results of the review?<br><br>(*Omit for QES*)	No	Minor limitation.
16. Did the review authors report any potential sources of conflict of interest, including any funding they received for conducting the review?	No.	Minor limitation. Reported as 'none' in the protocol, but not mentioned in the review.

**Overall assessment**	**Important limitations.**
**Explanation of overall assessment**	**One major limitation. Nine minor limitations.**

Criteria for assessing reviews (adapted from AMSTAR)

Review author, year: Chan et al., 2012.

Criterion	Assessment	Comments
1. Did the review have a clear research question and inclusion criteria?	Yes	
2. Did the report of the review contain an explicit statement that the review methods were established prior to the conduct of the review and did the report justify any significant deviations from the protocol?	No	No protocol found after searching Prospero and BMC systematic reviews. No relevant statement found. Minor limitation.
3. Did the review authors explain their selection of the study designs for inclusion in the review?	Yes	No selection made.
4. Did the review authors use a comprehensive literature search strategy?<br><br>*For Partial Yes (all the following):*<br>☐ **searched** at least 2 databases (relevant to research question)<br>☐ **provided** key word and/or search strategy<br>☐ justified publication restrictions (e.g. language)<br><br>*For Yes, should also have (all the following):*<br>☐ searched the reference lists / bibliographies of included studies<br>☐ included/consulted content experts in the field<br>☐ where relevant, searched for grey literature<br>☐ conducted search within 24 months of completion of the review	☐ Yes<br>☐ Partial Yes<br>☐ **No**	No language justification made. Minor limitation.

5. Did the review authors perform study selection in duplicate?	No	Minor limitation.
6. Did the review authors perform data extraction in duplicate?	No	Minor limitation.
7. Did the review authors provide a list of excluded studies and justify the exclusions?<br><br>*For Partial Yes:*<br>☐ provided a list of all potentially relevant studies that were read in full-text form but excluded from the review<br><br>*For Yes, must also have:*<br>☐ Justified the exclusion from the review of each potentially relevant study	☐ Yes<br>☐ Partial Yes<br>☐ **No**	Reasons for exclusion not provided for individual studies. Minor limitation.
8. Did the review authors describe the included studies in adequate detail?	Yes	In supplementary material.
9. Did the review authors use a satisfactory technique for assessing the methodological limitations / risk of bias (RoB) in individual studies that were included in the review?	No	Major limitation.
10. Did the review authors report on the sources of funding for the studies included in the review?	No	Minor limitation.
11. If a synthesis was performed did the review authors use appropriate methods to combine the results of individual studies?	Yes	
12. If synthesis was performed, did the review authors assess the potential impact of the methodological limitations / RoB in individual studies on the results of the evidence synthesis?	No	Minor limitation.
13. Did the review authors account for methodological limitations / RoB in individual studies when interpreting/ discussing the results of the review?	No	Minor limitation.

14. Did the review authors provide a satisfactory explanation for, and discussion of, any heterogeneity observed in the results of the review?<br><br>(*Omit for QES and for some reviews of survey data*)	No	Minor limitation.
15. If they performed quantitative synthesis did the review authors carry out an adequate investigation of publication bias (small study bias) and discuss its likely impact on the results of the review?<br><br>(*Omit for QES*)	No	Minor limitation.
16. Did the review authors report any potential sources of conflict of interest, including any funding they received for conducting the review?	No	Minor limitation.

**Overall assessment**	**Important limitations.**
**Explanation of overall assessment**	**One major limitation. Eleven minor limitations.**

Criteria for assessing reviews (adapted from AMSTAR)

Review author, year: Crocker-Buque et al., 2016.

**Criterion**	**Assessment**	**Comments**
1. Did the review have a clear research question and inclusion criteria?	Yes	
2. Did the report of the review contain an explicit statement that the review methods were established prior to the conduct of the review and did the report justify any significant deviations from the protocol?	Yes	Searches in Prospero and BMC systematic reviews yielded no results. No relevant statement found.
3. Did the review authors explain their selection of the study designs for inclusion in the review?	Yes	
4. Did the review authors use a comprehensive literature search strategy?<br><br>*For Partial Yes (all the following):*<br>☐ **searched** at least 2 databases (relevant to research question)<br>☐ **provided** key word and/or search strategy<br>☐ justified publication restrictions (e.g. language)<br><br>*For Yes, should also have (all the following):*<br>☐ searched the reference lists / bibliographies of included studies<br>☐ included/consulted content experts in the field<br>☐ where relevant, searched for grey literature<br>☐ conducted search within 24 months of completion of the review	☐ Yes<br>☐ Partial Yes<br>☐ **No**	Search strategy provided in supplementary material. Language restriction not justified. Minor limitation.

5. Did the review authors perform study selection in duplicate?	Yes	
6. Did the review authors perform data extraction in duplicate?	No	Not specified. Minor limitation.
7. Did the review authors provide a list of excluded studies and justify the exclusions?<br><br>*For Partial Yes:*<br>☐ provided a list of all potentially relevant studies that were read in full-text form but excluded from the review<br><br>*For Yes, must also have:*<br>☐ Justified the exclusion from the review of each potentially relevant study	☐ Yes<br>☐ Partial Yes<br>☐ **No**	PRISMA flow chart available. Minor limitation.
8. Did the review authors describe the included studies in adequate detail?	Yes	
9. Did the review authors use a satisfactory technique for assessing the methodological limitations / risk of bias (RoB) in individual studies that were included in the review?	No	Major limitation
10. Did the review authors report on the sources of funding for the studies included in the review?	No	Minor limitation
11. If a synthesis was performed did the review authors use appropriate methods to combine the results of individual studies?	Yes	
12. If synthesis was performed, did the review authors assess the potential impact of the methodological limitations / RoB in individual studies on the results of the evidence synthesis?	No	Minor limitation
13. Did the review authors account for methodological limitations / RoB in individual studies when interpreting/ discussing the results of the review?	No	Minor limitation

14. Did the review authors provide a satisfactory explanation for, and discussion of, any heterogeneity observed in the results of the review?<br><br>(*Omit for QES and for some reviews of survey data*)	Yes	Explains heterogeneity of interventions as a reason for not being able to draw firm conclusions. Minor limitation.
15. If they performed quantitative synthesis did the review authors carry out an adequate investigation of publication bias (small study bias) and discuss its likely impact on the results of the review?<br><br>(*Omit for QES*)	No	Minor limitation.
16. Did the review authors report any potential sources of conflict of interest, including any funding they received for conducting the review?	Yes	

**Overall assessment**	**Important limitations.**
**Explanation of overall assessment**	**One major limitation. Seven minor limitations.**

Criteria for assessing reviews (adapted from AMSTAR)

Review author, year: Cunningham et al., 2014.

Criterion	Assessment	Comments
1. Did the review have a clear research question and inclusion criteria?	Yes	
2. Did the report of the review contain an explicit statement that the review methods were established prior to the conduct of the review and did the report justify any significant deviations from the protocol?	No	Searches in Prospero and BMC Systematic Reviews yielded no results. No relevant statement found. Minor limitation.
3. Did the review authors explain their selection of the study designs for inclusion in the review?	Yes	No limit was placed on study design; however, included articles were required to report original data (i.e. not reviews, editorials or commentary).
4. Did the review authors use a comprehensive literature search strategy?<br><br>*For Partial Yes (all the following):*<br>☐ **searched** at least 2 databases (relevant to research question)<br>☐ **provided** key word and/or search strategy<br>☐ **justified** publication restrictions (e.g. language)<br><br>*For Yes, should also have (all the following):*<br>☐ **searched** the reference lists / bibliographies of included studies<br>☐ included/consulted content experts in the field<br>☐ where relevant, searched for grey literature<br>☐ **conducted** search within 24 months of completion of the review	☐ Yes<br>☐ **Partial** Yes<br>☐ No	Expert consultation and search for grey literature not reported. Minor limitation.

5. Did the review authors perform study selection in duplicate?	No	Minor limitation.
6. Did the review authors perform data extraction in duplicate?	No	Minor limitation.
7. Did the review authors provide a list of excluded studies and justify the exclusions?<br><br>*For Partial Yes:*<br>☐ provided a list of all potentially relevant studies that were read in full-text form but excluded from the review<br><br>*For Yes, must also have:*<br>☐ Justified the exclusion from the review of each potentially relevant study	☐ **Yes**<br>☐ Partial Yes<br>☐ No	Provided in appendix A.
8. Did the review authors describe the included studies in adequate detail?	Yes	
9. Did the review authors use a satisfactory technique for assessing the methodological limitations / risk of bias (RoB) in individual studies that were included in the review?	No	Major limitation.
10. Did the review authors report on the sources of funding for the studies included in the review?	No	Minor limitation.
11. If a synthesis was performed did the review authors use appropriate methods to combine the results of individual studies?	Yes	
12. If synthesis was performed, did the review authors assess the potential impact of the methodological limitations / RoB in individual studies on the results of the evidence synthesis?	No	Minor limitation
13. Did the review authors account for methodological limitations / RoB in individual studies when interpreting/ discussing the results of the review?	No	Minor limitation.

14. Did the review authors provide a satisfactory explanation for, and discussion of, any heterogeneity observed in the results of the review?<br><br>(*Omit for QES and for some reviews of survey data*)	No	Minor limitation.
15. If they performed quantitative synthesis did the review authors carry out an adequate investigation of publication bias (small study bias) and discuss its likely impact on the results of the review?<br><br>(*Omit for QES*)	No	Mentioned as a possible limitation, but not investigated. Minor limitation.
16. Did the review authors report any potential sources of conflict of interest, including any funding they received for conducting the review?	Yes	

**Overall assessment**	**Important limitations.**
**Explanation of overall assessment**	**One major limitation. Nine minor limitations.**

Criteria for assessing reviews (adapted from AMSTAR)

Review author, year: Das et al., 2016.

Criterion	Assessment	Comments
1. Did the review have a clear research question and inclusion criteria?	Yes	'to ascertain the effectiveness of interventions to improve immunization coverage among adolescents'<br><br>'For the purpose of this review, the adolescent population was defined as aged 11-19 years; however, since many studies targeted youth along with adolescents, exceptions were made to include studies targeting adolescents and youth. Based on the current recommended vaccines for adolescents [19], search was conducted to identify studies focusing on improving coverage for HPV; measles, mumps, rubella (MMR); TDaP; meningococcal conjugate vaccine; and varicella vaccines among adolescents and youth. Studies were excluded if they targeted age groups other than adolescents and youth or did not report segregated data for the age group of interest. Studies were excluded if the intervention was aimed at comparing the efficacy/effectiveness of different vaccine preparations, assessing changes in antibody titers in individual subjects, or comparing various modes of delivering vaccines without control or baseline data'
2. Did the report of the review contain an explicit statement that the review methods were established prior to the conduct of the review and did the report justify any significant deviations from the protocol?	No	Not reported
3. Did the review authors explain their selection of the study designs for inclusion in the review?	No	

4. Did the review authors use a comprehensive literature search strategy?<br><br>*For Partial Yes (all the following):*<br>☐ searched at least 2 databases (relevant to research question)<br>☐ provided key word and/or search strategy<br>☐ justified publication restrictions (e.g. language)<br><br>*For Yes, should also have (all the following):*<br>☐ searched the reference lists / bibliographies of included studies<br>☐ included/consulted content experts in the field<br>☐ where relevant, searched for grey literature<br>☐ conducted search within 24 months of completion of the review	☐ Yes<br>☐ Partial Yes<br>X No	Do not provide key words and / or search strategy

5. Did the review authors perform study selection in duplicate?	Yes	'The titles and abstracts of all studies identified were screened independently by two reviewers for relevance and matched. Any disagreements on selection of studies between these two primary abstractors were resolved by the third reviewer.' (pS43)
6. Did the review authors perform data extraction in duplicate?	Yes	'data from each study were abstracted independently and in duplicate into a standardized form. Studies that met the inclusion criteria were selected and double data abstracted on a standardized abstraction sheet.' (pS43)
7. Did the review authors provide a list of excluded studies and justify the exclusions?<br><br>*For Partial Yes:*<br>☐ provided a list of all potentially relevant studies that were read in full-text form but excluded from the review<br><br>*For Yes, must also have:*<br>☐ Justified the exclusion from the review of each potentially relevant study	☐ Yes<br>☐ Partial Yes<br>X No	No list provided
8. Did the review authors describe the included studies in adequate detail?	Yes	See Table 1
9. Did the review authors use a satisfactory technique for assessing the methodological limitations / risk of bias (RoB) in individual studies that were included in the review?	No	The authors note that 'Quality assessment of the included RCTs was done according to the Cochrane risk of bias assessment tool' (pS43) but do not explain how RoB was done for before-after studies or quasi-trials.
10. Did the review authors report on the sources of funding for the studies included in the review?	No	Not reported
11. If a synthesis was performed did the review authors use appropriate methods to combine the results of individual studies?	No	A meta-analysis was undertaken in which RCTs, quasi-RCTs and B-A studies were combined in a single analysis. In addition, the meta-analysis shows high stat heterogeneity, suggesting that pooling may not have been appropriate

12. If synthesis was performed, did the review authors assess the potential impact of the methodological limitations / RoB in individual studies on the results of the evidence synthesis?	No	RoB assessment for each study not reported, and not taken into account adequately in the results
13. Did the review authors account for methodological limitations / RoB in individual studies when interpreting/ discussing the results of the review?	Yes	Somewhat: 'However, these findings should be interpreted with caution since these are from single studies with low or very low quality.' (pS45)
14. Did the review authors provide a satisfactory explanation for, and discussion of, any heterogeneity observed in the results of the review?<br><br>(*Omit for QES and for some reviews of survey data*)	No	Not discussed
15. If they performed quantitative synthesis did the review authors carry out an adequate investigation of publication bias (small study bias) and discuss its likely impact on the results of the review?<br><br>(*Omit for QES*)	No	Not reported
16. Did the review authors report any potential sources of conflict of interest, including any funding they received for conducting the review?	Yes	Conflicts of interest and funding reported

**Overall assessment**	**Important limitations**
**Explanation of overall assessment**	**Limitations in relation to RoB assessment, analysis and interpretation which may undermine the reliability of the findings**

Criteria for assessing reviews (adapted from AMSTAR)

Review author, year: Ferrer et al., 2014.

Criterion	Assessment	Comments
1. Did the review have a clear research question and inclusion criteria?	Yes	
2. Did the report of the review contain an explicit statement that the review methods were established prior to the conduct of the review and did the report justify any significant deviations from the protocol?	No	Not found. Minor limitation.
3. Did the review authors explain their selection of the study designs for inclusion in the review?	Yes	
4. Did the review authors use a comprehensive literature search strategy?<br><br>*For Partial Yes (all the following):*<br>☐ **searched** at least 2 databases (relevant to research question)<br>☐ **provided** key word and/or search strategy<br>☐ justified publication restrictions (e.g. language)<br><br>*For Yes, should also have (all the following):*<br>☐ searched the reference lists / bibliographies of included studies<br>☐ included/consulted content experts in the field<br>☐ where relevant, searched for grey literature<br>☐ conducted search within 24 months of completion of the review	☐ Yes<br>☐ Partial Yes<br>☐ **No**	Language restriction not justified. Minor limitation.

5. Did the review authors perform study selection in duplicate?	Yes	
6. Did the review authors perform data extraction in duplicate?	No	Minor limitation.
7. Did the review authors provide a list of excluded studies and justify the exclusions?<br><br>*For Partial Yes:*<br>☐ provided a list of all potentially relevant studies that were read in full-text form but excluded from the review<br><br>*For Yes, must also have:*<br>☐ Justified the exclusion from the review of each potentially relevant study	☐ Yes<br>☐ Partial Yes<br>☐ **No**	Reasons included, but not a list of excluded studies. Minor limitation.
8. Did the review authors describe the included studies in adequate detail?	Yes	
9. Did the review authors use a satisfactory technique for assessing the methodological limitations / risk of bias (RoB) in individual studies that were included in the review?	Yes	
10. Did the review authors report on the sources of funding for the studies included in the review?	No	Minor limitation.
11. If a synthesis was performed did the review authors use appropriate methods to combine the results of individual studies?	Yes	
12. If synthesis was performed, did the review authors assess the potential impact of the methodological limitations / RoB in individual studies on the results of the evidence synthesis?	No	Minor limitation.
13. Did the review authors account for methodological limitations / RoB in individual studies when interpreting/ discussing the results of the review?	Yes	

14. Did the review authors provide a satisfactory explanation for, and discussion of, any heterogeneity observed in the results of the review?<br><br>(*Omit for QES and for some reviews of survey data*)	No	Minor limitation.
15. If they performed quantitative synthesis did the review authors carry out an adequate investigation of publication bias (small study bias) and discuss its likely impact on the results of the review?<br><br>(*Omit for QES*)	Not applicable.	
16. Did the review authors report any potential sources of conflict of interest, including any funding they received for conducting the review?	Yes	"The work was supported by the Centre for the Development and Evaluation of Complex Interventions for Public Health Improvement (DECIPHer) which receives funding from the British Heart Foundation, Cancer Research UK, Economic and Social Research Council (RES-590-28-0005), Medical Research Council, the Welsh Government and the Wellcome Trust (WT087640MA), under the auspices of the UK Clinical Research Collaboration. The funding bodies had no input into the design, analysis or interpretation of the data."

**Overall assessment**	**Minor limitations.**
**Explanation of overall assessment**	**Seven minor limitations.**

Criteria for assessing reviews (adapted from AMSTAR)

Review author, year: Francis et al., 2017.

**Criterion**	**Assessment**	**Comments**
1. Did the review have a clear research question and inclusion criteria?	Yes	
2. Did the report of the review contain an explicit statement that the review methods were established prior to the conduct of the review and did the report justify any significant deviations from the protocol?	No	Searches in Prospero and BMC Systematic Reviews yielded no results. No relevant statement found. Minor limitation.
3. Did the review authors explain their selection of the study designs for inclusion in the review?	Yes	
4. Did the review authors use a comprehensive literature search strategy?<br><br>*For Partial Yes (all the following):*<br>☐ **searched** at least 2 databases (relevant to research question)<br>☐ **provided** key word and/or search strategy<br>☐ **justified** publication restrictions (e.g. language)<br><br>*For Yes, should also have (all the following):*<br>☐ searched the reference lists / bibliographies of included studies<br>☐ included/consulted content experts in the field<br>☐ where relevant, searched for grey literature<br>☐ conducted search within 24 months of completion of the review	☐ Yes<br>☐ **Partial** Yes<br>☐ No	No major publication restrictions in need of justification. No search within reference lists other than three systematic reviews, for grey literature or consultation of field experts. Minor limitation.

5. Did the review authors perform study selection in duplicate?	Yes	
6. Did the review authors perform data extraction in duplicate?	No	Not specified. Minor limitation.
7. Did the review authors provide a list of excluded studies and justify the exclusions?<br><br>*For Partial Yes:*<br>☐ provided a list of all potentially relevant studies that were read in full-text form but excluded from the review<br><br>*For Yes, must also have:*<br>☐ Justified the exclusion from the review of each potentially relevant study	☐ Yes<br>☐ Partial Yes<br>☐ **No**	Excluded full texts categorized by reason for exclusion, but not listed. Minor limitation.
8. Did the review authors describe the included studies in adequate detail?	Yes	
9. Did the review authors use a satisfactory technique for assessing the methodological limitations / risk of bias (RoB) in individual studies that were included in the review?	No	Major limitation.
10. Did the review authors report on the sources of funding for the studies included in the review?	No	Minor limitation.
11. If a synthesis was performed did the review authors use appropriate methods to combine the results of individual studies?	Yes	
12. If synthesis was performed, did the review authors assess the potential impact of the methodological limitations / RoB in individual studies on the results of the evidence synthesis?	No	Minor limitation.
13. Did the review authors account for methodological limitations / RoB in individual studies when interpreting/ discussing the results of the review?	No	Minor limitation.

14. Did the review authors provide a satisfactory explanation for, and discussion of, any heterogeneity observed in the results of the review?<br><br>(*Omit for QES and for some reviews of survey data*)	Yes	
15. If they performed quantitative synthesis did the review authors carry out an adequate investigation of publication bias (small study bias) and discuss its likely impact on the results of the review?<br><br>(*Omit for QES*)	No	Minor limitation.
16. Did the review authors report any potential sources of conflict of interest, including any funding they received for conducting the review?	No	Minor limitation.

**Overall assessment**	**Important limitations.**
**Explanation of overall assessment**	**One major limitation. Nine minor limitations.**

Criteria for assessing reviews (adapted from AMSTAR)

Review author, year: Fu et al., 2014.

**Criterion**	**Assessment**	**Comments**
1. Did the review have a clear research question and inclusion criteria?	Yes	Clear inclusion criteria.
2. Did the report of the review contain an explicit statement that the review methods were established prior to the conduct of the review and did the report justify any significant deviations from the protocol?	No	Searches in Prospero and BMC Systematic Reviews yielded no results. No relevant statement found. Minor limitation.
3. Did the review authors explain their selection of the study designs for inclusion in the review?	No	Minor limitation.
4. Did the review authors use a comprehensive literature search strategy?<br><br>*For Partial Yes (all the following):*<br>☐ **searched** at least 2 databases (relevant to research question)<br>☐ **provided** key word and/or search strategy<br>☐ **justified** publication restrictions (e.g. language)<br><br>*For Yes, should also have (all the following):*<br>☐ **searched** the reference lists / bibliographies of included studies<br>☐ included/consulted content experts in the field<br>☐ where relevant, searched for grey literature<br>☐ **conducted** search within 24 months of completion of the review	☐ Yes<br>☐ **Partial** Yes<br>☐ No	Justified time as a publication restriction, but not language. Minor limitation.

5. Did the review authors perform study selection in duplicate?	Yes	
6. Did the review authors perform data extraction in duplicate?	Yes	
7. Did the review authors provide a list of excluded studies and justify the exclusions?<br><br>*For Partial Yes:*<br>☐ provided a list of all potentially relevant studies that were read in full-text form but excluded from the review<br><br>*For Yes, must also have:*<br>☐ Justified the exclusion from the review of each potentially relevant study	☐ Yes<br>☐ Partial Yes<br>☐ **No**	Minor limitation.
8. Did the review authors describe the included studies in adequate detail?	Yes	
9. Did the review authors use a satisfactory technique for assessing the methodological limitations / risk of bias (RoB) in individual studies that were included in the review?	Yes	Risk of bias in individual studies not found but reported done.
10. Did the review authors report on the sources of funding for the studies included in the review?	No	Minor limitation.
11. If a synthesis was performed did the review authors use appropriate methods to combine the results of individual studies?	Yes	
12. If synthesis was performed, did the review authors assess the potential impact of the methodological limitations / RoB in individual studies on the results of the evidence synthesis?	Yes	
13. Did the review authors account for methodological limitations / RoB in individual studies when interpreting/ discussing the results of the review?	Yes	

14. Did the review authors provide a satisfactory explanation for, and discussion of, any heterogeneity observed in the results of the review?<br><br>(*Omit for QES and for some reviews of survey data*)	Yes	
15. If they performed quantitative synthesis did the review authors carry out an adequate investigation of publication bias (small study bias) and discuss its likely impact on the results of the review?<br><br>(*Omit for QES*)	Not applicable.	Not performed.
16. Did the review authors report any potential sources of conflict of interest, including any funding they received for conducting the review?	Yes	

**Overall assessment**	**Minor limitations.**
**Explanation of overall assessment**	**Five minor limitations.**

Criteria for assessing reviews (adapted from AMSTAR)

Review author, year: Galbraith et al., 2016.

Criterion	Assessment	Comments
1. Did the review have a clear research question and inclusion criteria?	Yes	
2. Did the report of the review contain an explicit statement that the review methods were established prior to the conduct of the review and did the report justify any significant deviations from the protocol?	No	Searches in Prospero and BMC Systematic Reviews yielded no results. No relevant statement found. Minor limitation.
3. Did the review authors explain their selection of the study designs for inclusion in the review?	Not applicable.	No study design selection to explain.
4. Did the review authors use a comprehensive literature search strategy?<br><br>*For Partial Yes (all the following):*<br>☐ **searched** at least 2 databases (relevant to research question)<br>☐ **provided** key word and/or search strategy<br>☐ justified publication restrictions (e.g. language)<br><br>*For Yes, should also have (all the following):*<br>☐ searched the reference lists / bibliographies of included studies<br>☐ included/consulted content experts in the field<br>☐ where relevant, searched for grey literature<br>☐ conducted search within 24 months of completion of the review	☐ Yes<br>☐ **Partial** Yes<br>☐ No	No major publication restrictions.<br>Minor limitation.

5. Did the review authors perform study selection in duplicate?	No	Not specified. Minor limitation.
6. Did the review authors perform data extraction in duplicate?	No	Not specified. Minor limitation.
7. Did the review authors provide a list of excluded studies and justify the exclusions?<br><br>*For Partial Yes:*<br>☐ provided a list of all potentially relevant studies that were read in full-text form but excluded from the review<br><br>*For Yes, must also have:*<br>☐ Justified the exclusion from the review of each potentially relevant study	☐ Yes<br>☐ Partial Yes<br>☐ **No**	Minor limitation.
8. Did the review authors describe the included studies in adequate detail?	Yes	
9. Did the review authors use a satisfactory technique for assessing the methodological limitations / risk of bias (RoB) in individual studies that were included in the review?	No	Major limitation.
10. Did the review authors report on the sources of funding for the studies included in the review?	No	Minor limitation.
11. If a synthesis was performed did the review authors use appropriate methods to combine the results of individual studies?	Yes	
12. If synthesis was performed, did the review authors assess the potential impact of the methodological limitations / RoB in individual studies on the results of the evidence synthesis?	Yes	
13. Did the review authors account for methodological limitations / RoB in individual studies when interpreting/ discussing the results of the review?	No	Minor limitation.

14. Did the review authors provide a satisfactory explanation for, and discussion of, any heterogeneity observed in the results of the review?<br><br>(*Omit for QES and for some reviews of survey data*)	No	Minor limitation.
15. If they performed quantitative synthesis did the review authors carry out an adequate investigation of publication bias (small study bias) and discuss its likely impact on the results of the review?<br><br>(*Omit for QES*)	Not applicable.	Not performed.
16. Did the review authors report any potential sources of conflict of interest, including any funding they received for conducting the review?	Yes	Not funded.

**Overall assessment**	**Important limitations.**
**Explanation of overall assessment**	**One major limitation. Eight minor limitations.**

Criteria for assessing reviews (adapted from AMSTAR)

Review author, year: Gilkey et al., 2016.

Criterion	Assessment	Comments
1. Did the review have a clear research question and inclusion criteria?	Yes	
2. Did the report of the review contain an explicit statement that the review methods were established prior to the conduct of the review and did the report justify any significant deviations from the protocol?	No	Searches in Prospero and BMC Systematic Reviews yielded no results. No relevant statement found. Minor limitation.
3. Did the review authors explain their selection of the study designs for inclusion in the review?	Yes	No selection of study designs made.
4. Did the review authors use a comprehensive literature search strategy?<br><br>*For Partial Yes (all the following):*<br>☐ **searched** at least 2 databases (relevant to research question)<br>☐ **provided** key word and/or search strategy<br>☐ **justified** publication restrictions (e.g. language)<br><br>*For Yes, should also have (all the following):*<br>☐ **searched** the reference lists / bibliographies of included studies<br>☐ included/consulted content experts in the field<br>☐ where relevant, searched for grey literature<br>☐ **conducted** search within 24 months of completion of the review	☐ Yes<br>☐ **Partial** Yes<br>☐ No	Experts not consulted and grey literature not searched for. Minor limitation

5. Did the review authors perform study selection in duplicate?	No	One author reviewed titles and abstract, and another then checked them. Minor limitation
6. Did the review authors perform data extraction in duplicate?	No	One author reviewed the full texts, and another then checked them. Minor limitation
7. Did the review authors provide a list of excluded studies and justify the exclusions?<br><br>*For Partial Yes:*<br>☐ provided a list of all potentially relevant studies that were read in full-text form but excluded from the review<br><br>*For Yes, must also have:*<br>☐ Justified the exclusion from the review of each potentially relevant study	☐ Yes<br>☐ Partial Yes<br>☐ **No**	Reasons for exclusion reported by number, but not for individual studies. Minor limitation.
8. Did the review authors describe the included studies in adequate detail?	Yes	
9. Did the review authors use a satisfactory technique for assessing the methodological limitations / risk of bias (RoB) in individual studies that were included in the review?	No	Major limitation
10. Did the review authors report on the sources of funding for the studies included in the review?	No	Minor limitation.
11. If a synthesis was performed did the review authors use appropriate methods to combine the results of individual studies?	Yes	
12. If synthesis was performed, did the review authors assess the potential impact of the methodological limitations / RoB in individual studies on the results of the evidence synthesis?	No	Minor limitation.
13. Did the review authors account for methodological limitations / RoB in individual studies when interpreting/ discussing the results of the review?	Yes	

14. Did the review authors provide a satisfactory explanation for, and discussion of, any heterogeneity observed in the results of the review?<br><br>(*Omit for QES and for some reviews of survey data*)	No	Minor limitation.
15. If they performed quantitative synthesis did the review authors carry out an adequate investigation of publication bias (small study bias) and discuss its likely impact on the results of the review?<br><br>(*Omit for QES*)	Not applicable.	Not performed.
16. Did the review authors report any potential sources of conflict of interest, including any funding they received for conducting the review?	Yes	"This study was supported by a career development award from the National Cancer Institute (K22 CA186979). The funder did not play a role in study design, data analysis, report writing, or the decision to submit the article for publication."

**Overall assessment**	**Important limitations.**
**Explanation of overall assessment**	**One major limitation. Eight minor limitations.**

Criteria for assessing reviews (adapted from AMSTAR)

Review author, year: Hendry et al., 2013.

Criterion	Assessment	Comments
1. Did the review have a clear research question and inclusion criteria?	Yes	
2. Did the report of the review contain an explicit statement that the review methods were established prior to the conduct of the review and did the report justify any significant deviations from the protocol?	No	Searches in Prospero and BMC Systematic Reviews yielded no results. No relevant statement found. Minor limitation.
3. Did the review authors explain their selection of the study designs for inclusion in the review?	No	Minor limitation
4. Did the review authors use a comprehensive literature search strategy?<br><br>*For Partial Yes (all the following):*<br>☐ **searched** at least 2 databases (relevant to research question)<br>☐ **provided** key word and/or search strategy<br>☐ **justified** publication restrictions (e.g. language)<br><br>*For Yes, should also have (all the following):*<br>☐ searched the reference lists / bibliographies of included studies<br>☐ included/consulted content experts in the field<br>☐ where relevant, searched for grey literature<br>☐ conducted search within 24 months of completion of the review	☐ Yes<br>☐ **Partial** Yes<br>☐ No	Time limit explained. No other major publication restrictions set. Minor limitation.

5. Did the review authors perform study selection in duplicate?	Yes	
6. Did the review authors perform data extraction in duplicate?	No	Done collaboratively by the review team. Minor limitation.
7. Did the review authors provide a list of excluded studies and justify the exclusions?<br><br>*For Partial Yes:*<br>☐ provided a list of all potentially relevant studies that were read in full-text form but excluded from the review<br><br>*For Yes, must also have:*<br>☐ Justified the exclusion from the review of each potentially relevant study	☐ Yes<br>☐ Partial Yes<br>☐ **No**	Minor limitation.
8. Did the review authors describe the included studies in adequate detail?	Yes	
9. Did the review authors use a satisfactory technique for assessing the methodological limitations / risk of bias (RoB) in individual studies that were included in the review?	Yes	For qualitative studies.
10. Did the review authors report on the sources of funding for the studies included in the review?	Yes	For qualitative studies.
11. If a synthesis was performed did the review authors use appropriate methods to combine the results of individual studies?	Yes	
12. If synthesis was performed, did the review authors assess the potential impact of the methodological limitations / RoB in individual studies on the results of the evidence synthesis?	No	Minor limitation.
13. Did the review authors account for methodological limitations / RoB in individual studies when interpreting/ discussing the results of the review?	No	Minor limitation.

14. Did the review authors provide a satisfactory explanation for, and discussion of, any heterogeneity observed in the results of the review?<br><br>(*Omit for QES and for some reviews of survey data*)	Yes	Satisfactory explanation for heterogeneity of populations.
15. If they performed quantitative synthesis did the review authors carry out an adequate investigation of publication bias (small study bias) and discuss its likely impact on the results of the review?<br><br>(*Omit for QES*)	Not applicable.	Not performed.
16. Did the review authors report any potential sources of conflict of interest, including any funding they received for conducting the review?	Yes	"This review is part of the multi-method HPV Core Messages project, funded by Cancer Research UK (Ref. C1273/A9479) to inform the development of evidence-based informational material for use in the context of HPV vaccination and testing programmes."

**Overall assessment**	**Minor limitations.**
**Explanation of overall assessment**	**Seven minor limitations.**

Criteria for assessing reviews (adapted from AMSTAR)

Review author, year: Holman et al., 2014.

Criterion	Assessment	Comments
1. Did the review have a clear research question and inclusion criteria?	No	Research question is clear. Inclusion criteria not reported. Minor limitation.
2. Did the report of the review contain an explicit statement that the review methods were established prior to the conduct of the review and did the report justify any significant deviations from the protocol?	No	Searches in Prospero and BMC Systematic Reviews yielded no results. No relevant statement found. Minor limitation.
3. Did the review authors explain their selection of the study designs for inclusion in the review?	Yes	No selection of study design mentioned.
4. Did the review authors use a comprehensive literature search strategy?<br><br>*For Partial Yes (all the following):*<br>☐ searched at least 2 databases (relevant to research question)<br>☐ provided key word and/or search strategy<br>☐ justified publication restrictions (e.g. language)<br><br>*For Yes, should also have (all the following):*<br>☐ searched the reference lists / bibliographies of included studies<br>☐ included/consulted content experts in the field<br>☐ where relevant, searched for grey literature<br>☐ conducted search within 24 months of completion of the review	☐ Yes<br>☐ Partial Yes<br>☐ **No**	Only searched one database, but bibliographies of selected articles for additional relevant studies. Major limitation.

5. Did the review authors perform study selection in duplicate?	No	Only one author reviewed the titles and abstracts of all articles returned in the search and retrieved the full texts of potentially relevant articles to make a final determination of their relevance. Minor limitation.
6. Did the review authors perform data extraction in duplicate?	Yes	
7. Did the review authors provide a list of excluded studies and justify the exclusions?<br><br>*For Partial Yes:*<br>☐ provided a list of all potentially relevant studies that were read in full-text form but excluded from the review<br><br>*For Yes, must also have:*<br>☐ Justified the exclusion from the review of each potentially relevant study	☐ Yes<br>☐ Partial Yes<br>☐ **No**	Minor limitation
8. Did the review authors describe the included studies in adequate detail?	No	No summary of details of included studies with information such as study design and population. Major limitation.
9. Did the review authors use a satisfactory technique for assessing the methodological limitations / risk of bias (RoB) in individual studies that were included in the review?	No	Major limitation.
10. Did the review authors report on the sources of funding for the studies included in the review?	No	Minor limitation.
11. If a synthesis was performed did the review authors use appropriate methods to combine the results of individual studies?	No	Minor limitation.
12. If synthesis was performed, did the review authors assess the potential impact of the methodological limitations / RoB in individual studies on the results of the evidence synthesis?	No	Minor limitation.

13. Did the review authors account for methodological limitations / RoB in individual studies when interpreting/ discussing the results of the review?	No	Minor limitation.
14. Did the review authors provide a satisfactory explanation for, and discussion of, any heterogeneity observed in the results of the review?<br><br>(*Omit for QES and for some reviews of survey data*)	No	No satisfactory explanation for the heterogeneity observed in some of the results. Minor limitation.
15. If they performed quantitative synthesis did the review authors carry out an adequate investigation of publication bias (small study bias) and discuss its likely impact on the results of the review?<br><br>(*Omit for QES*)	Not applicable.	Not performed.
16. Did the review authors report any potential sources of conflict of interest, including any funding they received for conducting the review?	Yes	"Conflict of Interest of Disclosures: None reported."

**Overall assessment**	**Important limitations.**
**Explanation of overall assessment**	**Two major limitations. Nine minor limitations.**

Criteria for assessing reviews (adapted from AMSTAR)

Review author, year: Hyde et al., 2012.

Criterion	Assessment	Comments
1. Did the review have a clear research question and inclusion criteria?	Yes	
2. Did the report of the review contain an explicit statement that the review methods were established prior to the conduct of the review and did the report justify any significant deviations from the protocol?	No	Searches in Prospero and BMC Systematic Reviews yielded no results. No relevant statement found. Minor limitation.
3. Did the review authors explain their selection of the study designs for inclusion in the review?	Yes	Reason for excluding clinical trials explained.
4. Did the review authors use a comprehensive literature search strategy?<br><br>*For Partial Yes (all the following):*<br>☐ **searched** at least 2 databases (relevant to research question)<br>☐ **provided** key word and/or search strategy<br>☐ justified publication restrictions (e.g. language)<br><br>*For Yes, should also have (all the following):*<br>☐ searched the reference lists / bibliographies of included studies<br>☐ included/consulted content experts in the field<br>☐ where relevant, searched for grey literature<br>☐ conducted search within 24 months of completion of the review	☐ Yes<br>☐ Partial Yes<br>☐ **No**	Language restriction not justified. Minor limitation.

5. Did the review authors perform study selection in duplicate?	No	Not specified. Minor limitation.
6. Did the review authors perform data extraction in duplicate?	No	Not specified. Minor limitation.
7. Did the review authors provide a list of excluded studies and justify the exclusions?<br><br>*For Partial Yes:*<br>☐ provided a list of all potentially relevant studies that were read in full-text form but excluded from the review<br><br>*For Yes, must also have:*<br>☐ Justified the exclusion from the review of each potentially relevant study	☐ Yes<br>☐ Partial Yes<br>☐ **No**	Minor limitation.
8. Did the review authors describe the included studies in adequate detail?	No	Information such as study design and population not reported adequately. Minor limitation.
9. Did the review authors use a satisfactory technique for assessing the methodological limitations / risk of bias (RoB) in individual studies that were included in the review?	No	Major limitation.
10. Did the review authors report on the sources of funding for the studies included in the review?	No	Minor limitation.
11. If a synthesis was performed did the review authors use appropriate methods to combine the results of individual studies?	Yes	
12. If synthesis was performed, did the review authors assess the potential impact of the methodological limitations / RoB in individual studies on the results of the evidence synthesis?	No	Minor limitation.
13. Did the review authors account for methodological limitations / RoB in individual studies when interpreting/ discussing the results of the review?	No	Minor limitation.

14. Did the review authors provide a satisfactory explanation for, and discussion of, any heterogeneity observed in the results of the review?<br><br>(*Omit for QES and for some reviews of survey data*)	No	Minor limitation.
15. If they performed quantitative synthesis did the review authors carry out an adequate investigation of publication bias (small study bias) and discuss its likely impact on the results of the review?<br><br>(*Omit for QES*)	Not applicable.	Not performed.
16. Did the review authors report any potential sources of conflict of interest, including any funding they received for conducting the review?	No	Minor limitation.

**Overall assessment**	**Important limitations.**
**Explanation of overall assessment**	**One major limitation. Eleven minor limitations.**

Criteria for assessing reviews (adapted from AMSTAR)

Review author, year: Johnson et al., 2018.

Criterion	Assessment	Comments
1. Did the review have a clear research question and inclusion criteria?	Yes	
2. Did the report of the review contain an explicit statement that the review methods were established prior to the conduct of the review and did the report justify any significant deviations from the protocol?	No	Searches in Prospero and BMC systematic reviews yielded no results. No relevant statement found. Minor limitation.
3. Did the review authors explain their selection of the study designs for inclusion in the review?	No	Minor limitation.
4. Did the review authors use a comprehensive literature search strategy?<br><br>*For Partial Yes (all the following):*<br>☐ **searched** at least 2 databases (relevant to research question)<br>☐ **provided** key word and/or search strategy<br>☐ justified publication restrictions (e.g. language)<br><br>*For Yes, should also have (all the following):*<br>☐ searched the reference lists / bibliographies of included studies<br>☐ included/consulted content experts in the field<br>☐ where relevant, searched for grey literature<br>☐ conducted search within 24 months of completion of the review	☐ Yes<br>☐ Partial Yes<br>☐ **No**	Language restriction not justified. Minor limitation.

5. Did the review authors perform study selection in duplicate?	Yes	
6. Did the review authors perform data extraction in duplicate?	No	Only for a sample of initial articles. Minor limitation.
7. Did the review authors provide a list of excluded studies and justify the exclusions?<br><br>*For Partial Yes:*<br>☐ provided a list of all potentially relevant studies that were read in full-text form but excluded from the review<br><br>*For Yes, must also have:*<br>☐ Justified the exclusion from the review of each potentially relevant study	☐ Yes<br>☐ Partial Yes<br>☐ **No**	Reasons listed, but not per review. Minor limitation.
8. Did the review authors describe the included studies in adequate detail?	Yes	
9. Did the review authors use a satisfactory technique for assessing the methodological limitations / risk of bias (RoB) in individual studies that were included in the review?	No	A satisfactory technique for assessing risk of bias was used and also reported for the individual studie. The details were on the other hand not included. Minor limitation.
10. Did the review authors report on the sources of funding for the studies included in the review?	No	Minor limitation.
11. If a synthesis was performed did the review authors use appropriate methods to combine the results of individual studies?	Yes	
12. If synthesis was performed, did the review authors assess the potential impact of the methodological limitations / RoB in individual studies on the results of the evidence synthesis?	Yes	
13. Did the review authors account for methodological limitations / RoB in individual studies when interpreting/ discussing the results of the review?	Yes	

14. Did the review authors provide a satisfactory explanation for, and discussion of, any heterogeneity observed in the results of the review?<br><br>(*Omit for QES and for some reviews of survey data*)	No	Minor limitation
15. If they performed quantitative synthesis did the review authors carry out an adequate investigation of publication bias (small study bias) and discuss its likely impact on the results of the review?<br><br>(*Omit for QES*)	Not applicable	
16. Did the review authors report any potential sources of conflict of interest, including any funding they received for conducting the review?	Yes	

**Overall assessment**	**Minor limitations.**
**Explanation of overall assessment**	**Eight minor limitations.**

Criteria for assessing reviews (adapted from AMSTAR)

Review author, year: Kabakama et al., 2016.

Criterion	Assessment	Comments
1. Did the review have a clear research question and inclusion criteria?	Yes	
2. Did the report of the review contain an explicit statement that the review methods were established prior to the conduct of the review and did the report justify any significant deviations from the protocol?	No	Searches in Prospero and BMC Systematic Reviews yielded no results. No relevant statement found. Minor limitation.
3. Did the review authors explain their selection of the study designs for inclusion in the review?	Yes	No selection of study design.
4. Did the review authors use a comprehensive literature search strategy?<br><br>*For Partial Yes (all the following):*<br>☐ **searched** at least 2 databases (relevant to research question)<br>☐ **provided** key word and/or search strategy<br>☐ justified publication restrictions (e.g. language)<br><br>*For Yes, should also have (all the following):*<br>☐ searched the reference lists / bibliographies of included studies<br>☐ included/consulted content experts in the field<br>☐ where relevant, searched for grey literature<br>☐ conducted search within 24 months of completion of the review	☐ Yes<br>☐ Partial Yes<br>☐ **No**	Time restriction not explained. Minor limitation.

5. Did the review authors perform study selection in duplicate?	No	Not specified. Minor limitation.
6. Did the review authors perform data extraction in duplicate?	No	Not specified. Minor limitation.
7. Did the review authors provide a list of excluded studies and justify the exclusions?<br><br>*For Partial Yes:*<br>☐ provided a list of all potentially relevant studies that were read in full-text form but excluded from the review<br><br>*For Yes, must also have:*<br>☐ Justified the exclusion from the review of each potentially relevant study	☐ Yes<br>☐ Partial Yes<br>☐ **No**	Minor limitation.
8. Did the review authors describe the included studies in adequate detail?	No	Information such as populations and study designs included not to be found. Minor limitation.
9. Did the review authors use a satisfactory technique for assessing the methodological limitations / risk of bias (RoB) in individual studies that were included in the review?	No	Major limitation.
10. Did the review authors report on the sources of funding for the studies included in the review?	No	Minor limitation.
11. If a synthesis was performed did the review authors use appropriate methods to combine the results of individual studies?	Yes	
12. If synthesis was performed, did the review authors assess the potential impact of the methodological limitations / RoB in individual studies on the results of the evidence synthesis?	No	Minor limitation.
13. Did the review authors account for methodological limitations / RoB in individual studies when interpreting/ discussing the results of the review?	No	Minor limitation.

14. Did the review authors provide a satisfactory explanation for, and discussion of, any heterogeneity observed in the results of the review?<br><br>(*Omit for QES and for some reviews of survey data*)	Yes	No major heterogeneity observed.
15. If they performed quantitative synthesis did the review authors carry out an adequate investigation of publication bias (small study bias) and discuss its likely impact on the results of the review?<br><br>(*Omit for QES*)	Not applicable.	Not performed.
16. Did the review authors report any potential sources of conflict of interest, including any funding they received for conducting the review?	Yes	This publication is based on research funded by the Bill & Melinda Gates Foundation.

**Overall assessment**	**Important limitations.**
**Explanation of overall assessment**	**One major limitation. Nine minor limitations.**

Criteria for assessing reviews (adapted from AMSTAR)

Review author, year: Kang et al., 2018.

Criterion	Assessment	Comments
1. Did the review have a clear research question and inclusion criteria?	Yes	
2. Did the report of the review contain an explicit statement that the review methods were established prior to the conduct of the review and did the report justify any significant deviations from the protocol?	No	Searches in Prospero and BMC systematic reviews yielded no results. No relevant statement found. Minor limitation.
3. Did the review authors explain their selection of the study designs for inclusion in the review?	No	Minor limitation.
4. Did the review authors use a comprehensive literature search strategy?<br><br>*For Partial Yes (all the following):*<br>☐ **searched** at least 2 databases (relevant to research question)<br>☐ **provided** key word and/or search strategy<br>☐ justified publication restrictions (e.g. language)<br><br>*For Yes, should also have (all the following):*<br>☐ searched the reference lists / bibliographies of included studies<br>☐ included/consulted content experts in the field<br>☐ where relevant, searched for grey literature<br>☐ conducted search within 24 months of completion of the review	☐ Yes<br>☐ Partial Yes<br>☐ **No**	Language restriction not justified. Minor limitation.

5. Did the review authors perform study selection in duplicate?	No	Minor limitation.
6. Did the review authors perform data extraction in duplicate?	No	Minor limitation.
7. Did the review authors provide a list of excluded studies and justify the exclusions?<br><br>*For Partial Yes:*<br>☐ provided a list of all potentially relevant studies that were read in full-text form but excluded from the review<br><br>*For Yes, must also have:*<br>☐ Justified the exclusion from the review of each potentially relevant study	☐ Yes<br>☐ Partial Yes<br>☐ **No**	Reasons provided, but not for each study. A list of excluded studies is not provided. Minor limitation.
8. Did the review authors describe the included studies in adequate detail?	Yes	
9. Did the review authors use a satisfactory technique for assessing the methodological limitations / risk of bias (RoB) in individual studies that were included in the review?	Yes	
10. Did the review authors report on the sources of funding for the studies included in the review?	No	Minor limitation.
11. If a synthesis was performed did the review authors use appropriate methods to combine the results of individual studies?	Yes	
12. If synthesis was performed, did the review authors assess the potential impact of the methodological limitations / RoB in individual studies on the results of the evidence synthesis?	Yes	
13. Did the review authors account for methodological limitations / RoB in individual studies when interpreting/ discussing the results of the review?	Yes	

14. Did the review authors provide a satisfactory explanation for, and discussion of, any heterogeneity observed in the results of the review?<br><br>(*Omit for QES and for some reviews of survey data*)	Yes	
15. If they performed quantitative synthesis did the review authors carry out an adequate investigation of publication bias (small study bias) and discuss its likely impact on the results of the review?<br><br>(*Omit for QES*)	Not applicable.	
16. Did the review authors report any potential sources of conflict of interest, including any funding they received for conducting the review?	Yes	

**Overall assessment**	**Minor limitations.**
**Explanation of overall assessment**	**Seven minor limitations.**

Criteria for assessing reviews (adapted from AMSTAR)

Review author, year: Karafillakis et al., 2017.

Criterion	Assessment	Comments
1. Did the review have a clear research question and inclusion criteria?	Yes	
2. Did the report of the review contain an explicit statement that the review methods were established prior to the conduct of the review and did the report justify any significant deviations from the protocol?	No	Not found. Minor limitation.
3. Did the review authors explain their selection of the study designs for inclusion in the review?	No	Minor limitation.
4. Did the review authors use a comprehensive literature search strategy?<br><br>*For Partial Yes (all the following):*<br>☐ **searched** at least 2 databases (relevant to research question)<br>☐ **provided** key word and/or search strategy<br>☐ justified publication restrictions (e.g. language)<br><br>*For Yes, should also have (all the following):*<br>☐ searched the reference lists / bibliographies of included studies<br>☐ included/consulted content experts in the field<br>☐ where relevant, searched for grey literature<br>☐ conducted search within 24 months of completion of the review	☐ Yes<br>☐ Partial Yes<br>☐ **No**	Time and language restrictions not justified. Minor limitation.

5. Did the review authors perform study selection in duplicate?	No	Minor limitation.
6. Did the review authors perform data extraction in duplicate?	No	Not specified. Minor limitation.
7. Did the review authors provide a list of excluded studies and justify the exclusions?<br><br>*For Partial Yes:*<br>☐ provided a list of all potentially relevant studies that were read in full-text form but excluded from the review<br><br>*For Yes, must also have:*<br>☐ Justified the exclusion from the review of each potentially relevant study	☐ Yes<br>☐ Partial Yes<br>☐ **No**	Reasons said to be included, but not found in article or online. Minor limitation.
8. Did the review authors describe the included studies in adequate detail?	No	Minor limitation.
9. Did the review authors use a satisfactory technique for assessing the methodological limitations / risk of bias (RoB) in individual studies that were included in the review?	No	Major limitation.
10. Did the review authors report on the sources of funding for the studies included in the review?	No	Minor limitation.
11. If a synthesis was performed did the review authors use appropriate methods to combine the results of individual studies?	Yes	
12. If synthesis was performed, did the review authors assess the potential impact of the methodological limitations / RoB in individual studies on the results of the evidence synthesis?	No	Minor limitation.
13. Did the review authors account for methodological limitations / RoB in individual studies when interpreting/ discussing the results of the review?	No	Minor limitation.

14. Did the review authors provide a satisfactory explanation for, and discussion of, any heterogeneity observed in the results of the review?<br><br>(*Omit for QES and for some reviews of survey data*)	No	Minor limitation.
15. If they performed quantitative synthesis did the review authors carry out an adequate investigation of publication bias (small study bias) and discuss its likely impact on the results of the review?<br><br>(*Omit for QES*)	Not applicable.	
16. Did the review authors report any potential sources of conflict of interest, including any funding they received for conducting the review?	Yes	"This study was conducted under the ADVANCE project, by the Innovative Medicines Initiative (Grant agreement n 115557), resources of which are composed of financial contribution from the European Union's Seventh Framework Programme (FP7/2007- 2013) and EFPIA companies' in kind contribution. The funder had no involvement in the study design, collection, analysis, interpreta- tion of data, in the writing of the report, and in the decision to sub- mit the article for publication."

**Overall assessment**	**Important limitations.**
**Explanation of overall assessment**	**One major limitation. Eleven minor limitations.**

Criteria for assessing reviews (adapted from AMSTAR)

Review author, year: Kessels et al., 2012.

Criterion	Assessment	Comments
1. Did the review have a clear research question and inclusion criteria?	Yes	
2. Did the report of the review contain an explicit statement that the review methods were established prior to the conduct of the review and did the report justify any significant deviations from the protocol?	No	Searches in Prospero and BMC Systematic Reviews yielded no results. No relevant statement found. Minor limitation.
3. Did the review authors explain their selection of the study designs for inclusion in the review?	No	Minor limitation.
4. Did the review authors use a comprehensive literature search strategy?<br><br>*For Partial Yes (all the following):*<br>☐ **searched** at least 2 databases (relevant to research question)<br>☐ **provided** key word and/or search strategy<br>☐ **justified** publication restrictions (e.g. language)<br><br>*For Yes, should also have (all the following):*<br>☐ searched the reference lists / bibliographies of included studies<br>☐ included/consulted content experts in the field<br>☐ where relevant, searched for grey literature<br>☐ conducted search within 24 months of completion of the review	☐ Yes<br>☐ **Partial** Yes<br>☐ No	Minor limitation.

5. Did the review authors perform study selection in duplicate?	No	Not specified. Minor limitation.
6. Did the review authors perform data extraction in duplicate?	No	Minor limitation.
7. Did the review authors provide a list of excluded studies and justify the exclusions?<br><br>*For Partial Yes:*<br>☐ provided a list of all potentially relevant studies that were read in full-text form but excluded from the review<br><br>*For Yes, must also have:*<br>☐ Justified the exclusion from the review of each potentially relevant study	☐ Yes<br>☐ Partial Yes<br>☐ **No**	Minor limitation.
8. Did the review authors describe the included studies in adequate detail?	Yes	
9. Did the review authors use a satisfactory technique for assessing the methodological limitations / risk of bias (RoB) in individual studies that were included in the review?	Yes	
10. Did the review authors report on the sources of funding for the studies included in the review?	No	Minor limitation.
11. If a synthesis was performed did the review authors use appropriate methods to combine the results of individual studies?	Yes	
12. If synthesis was performed, did the review authors assess the potential impact of the methodological limitations / RoB in individual studies on the results of the evidence synthesis?	Yes	
13. Did the review authors account for methodological limitations / RoB in individual studies when interpreting/ discussing the results of the review?	Yes	

14. Did the review authors provide a satisfactory explanation for, and discussion of, any heterogeneity observed in the results of the review?<br><br>(*Omit for QES and for some reviews of survey data*)	Yes	
15. If they performed quantitative synthesis did the review authors carry out an adequate investigation of publication bias (small study bias) and discuss its likely impact on the results of the review?<br><br>(*Omit for QES*)	Not applicable.	
16. Did the review authors report any potential sources of conflict of interest, including any funding they received for conducting the review?	No	Minor limitation.

**Overall assessment**	**Important limitations.**
**Explanation of overall assessment**	**Eight minor limitations.**

Criteria for assessing reviews (adapted from AMSTAR)

Review author, year: Kim et al., 2017.

Criterion	Assessment	Comments
1. Did the review have a clear research question and inclusion criteria?	Yes	
2. Did the report of the review contain an explicit statement that the review methods were established prior to the conduct of the review and did the report justify any significant deviations from the protocol?	No	Searches in Prospero and BMC Systematic Reviews yielded no results. No relevant statement found. Minor limitation.
3. Did the review authors explain their selection of the study designs for inclusion in the review?	Yes	No selection of study design made.
4. Did the review authors use a comprehensive literature search strategy?<br><br>*For Partial Yes (all the following):*<br>☐ **searched** at least 2 databases (relevant to research question)<br>☐ **provided** key word and/or search strategy<br>☐ **justified** publication restrictions (e.g. language)<br><br>*For Yes, should also have (all the following):*<br>☐ searched the reference lists / bibliographies of included studies<br>☐ included/consulted content experts in the field<br>☐ where relevant, searched for grey literature<br>☐ conducted search within 24 months of completion of the review	☐ Yes<br>☐ **Partial** Yes<br>☐ No	Minor limitation.w

5. Did the review authors perform study selection in duplicate?	No	Abstract screening: No. Full-text screening: Yes. Minor limitation.
6. Did the review authors perform data extraction in duplicate?	No	Minor limitation.
7. Did the review authors provide a list of excluded studies and justify the exclusions?<br><br>*For Partial Yes:*<br>☐ provided a list of all potentially relevant studies that were read in full-text form but excluded from the review<br><br>*For Yes, must also have:*<br>☐ Justified the exclusion from the review of each potentially relevant study	☐ Yes<br>☐ Partial Yes<br>☐ **No**	Minor limitation
8. Did the review authors describe the included studies in adequate detail?	Yes	
9. Did the review authors use a satisfactory technique for assessing the methodological limitations / risk of bias (RoB) in individual studies that were included in the review?	Yes	STROBE checklist used to assess the methodological rigor of studies selected in this review.
10. Did the review authors report on the sources of funding for the studies included in the review?	No	Minor limitation.
11. If a synthesis was performed did the review authors use appropriate methods to combine the results of individual studies?	Yes	
12. If synthesis was performed, did the review authors assess the potential impact of the methodological limitations / RoB in individual studies on the results of the evidence synthesis?	Yes	
13. Did the review authors account for methodological limitations / RoB in individual studies when interpreting/ discussing the results of the review?	No	Minor limitation.

14. Did the review authors provide a satisfactory explanation for, and discussion of, any heterogeneity observed in the results of the review?<br><br>(*Omit for QES and for some reviews of survey data*)	No	No satisfactory explanation made for the heterogeneity observed in the results. Minor limitation.
15. If they performed quantitative synthesis did the review authors carry out an adequate investigation of publication bias (small study bias) and discuss its likely impact on the results of the review?<br><br>(*Omit for QES*)	Not applicable.	Not performed due to heterogeneity of settings and sample characteristics.
16. Did the review authors report any potential sources of conflict of interest, including any funding they received for conducting the review?	No	"The authors have no funding support to report. "

**Overall assessment**	**Minor limitations.**
**Explanation of overall assessment**	**Eight minor limitations.**

Criteria for assessing reviews (adapted from AMSTAR)

Review author, year: Loke et al., 2017.

Criterion	Assessment	Comments
1. Did the review have a clear research question and inclusion criteria?	Yes	
2. Did the report of the review contain an explicit statement that the review methods were established prior to the conduct of the review and did the report justify any significant deviations from the protocol?	No	Not mentioned and no protocol to be found in Prospero or BMC Systematic Reviews. Minor limitation.
3. Did the review authors explain their selection of the study designs for inclusion in the review?	No	Reasoning not explained. Minor limitation.
4. Did the review authors use a comprehensive literature search strategy?<br><br>*For Partial Yes (all the following):*<br>☐ **searched** at least 2 databases (relevant to research question)<br>☐ **provided** key word and/or search strategy<br>☐ **justified** publication restrictions (e.g. language)<br><br>*For Yes, should also have (all the following):*<br>☐ searched the reference lists / bibliographies of included studies<br>☐ included/consulted content experts in the field<br>☐ where relevant, searched for grey literature<br>☐ **conducted** search within 24 months of completion of the review	☐ Yes<br>☐ **Partial** Yes<br>☐ No	Fulfills a partial yes, but did not search reference lists, consult content experts or search for grey literature. Minor limitation.

5. Did the review authors perform study selection in duplicate?	No	Not specified. Minor limitation.
6. Did the review authors perform data extraction in duplicate?	Yes	
7. Did the review authors provide a list of excluded studies and justify the exclusions?<br><br>*For Partial Yes:*<br>☐ provided a list of all potentially relevant studies that were read in full-text form but excluded from the review<br><br>*For Yes, must also have:*<br>☐ Justified the exclusion from the review of each potentially relevant study	☐ Yes<br>☐ Partial Yes<br>☐ **No**	Provided number of excluded studies with reasons in their PRISMA flow diagram. Minor limitation.
8. Did the review authors describe the included studies in adequate detail?	Yes	Yes, but only for 28 out of the 42 included studies.
9. Did the review authors use a satisfactory technique for assessing the methodological limitations / risk of bias (RoB) in individual studies that were included in the review?	Yes	
10. Did the review authors report on the sources of funding for the studies included in the review?	No	Minor limitation.
11. If a synthesis was performed did the review authors use appropriate methods to combine the results of individual studies?	Yes	
12. If synthesis was performed, did the review authors assess the potential impact of the methodological limitations / RoB in individual studies on the results of the evidence synthesis?	Yes	
13. Did the review authors account for methodological limitations / RoB in individual studies when interpreting/ discussing the results of the review?	No	Major limitation.

14. Did the review authors provide a satisfactory explanation for, and discussion of, any heterogeneity observed in the results of the review?<br><br>(*Omit for QES and for some reviews of survey data*)	Yes	Heterogeneity of the population and the inconsistent variables mentioned as a reason for not being able to pool the results for statistical significance.
15. If they performed quantitative synthesis did the review authors carry out an adequate investigation of publication bias (small study bias) and discuss its likely impact on the results of the review?<br><br>(*Omit for QES*)	No	Minor limitation.
16. Did the review authors report any potential sources of conflict of interest, including any funding they received for conducting the review?	Yes	

**Overall assessment**	Does not meet threshold.
**Explanation of overall assessment**	**Two major limitations. Seven minor limitations.**

Criteria for assessing reviews (adapted from AMSTAR)

Review author, year: Mishra, A. 2011.

Criterion	Assessment	Comments
1. Did the review have a clear research question and inclusion criteria?	Yes	
2. Did the report of the review contain an explicit statement that the review methods were established prior to the conduct of the review and did the report justify any significant deviations from the protocol?	No	Searches in Prospero and BMC Systematic Reviews yielded no results. No relevant statement found. Minor limitation.
3. Did the review authors explain their selection of the study designs for inclusion in the review?	Yes	
4. Did the review authors use a comprehensive literature search strategy?<br><br>*For Partial Yes (all the following):*<br>☐ searched at least 2 databases (relevant to research question)<br>☐ provided key word and/or search strategy<br>☐ justified publication restrictions (e.g. language)<br><br>*For Yes, should also have (all the following):*<br>☐ searched the reference lists / bibliographies of included studies<br>☐ included/consulted content experts in the field<br>☐ where relevant, searched for grey literature<br>☐ conducted search within 24 months of completion of the review	☐ Yes<br>☐ Partial Yes<br>☐ **No**	Only one database searched. Major limitation.

5. Did the review authors perform study selection in duplicate?	No	Minor limitation.
6. Did the review authors perform data extraction in duplicate?	No	Minor limitation.
7. Did the review authors provide a list of excluded studies and justify the exclusions?<br><br>*For Partial Yes:*<br>☐ provided a list of all potentially relevant studies that were read in full-text form but excluded from the review<br><br>*For Yes, must also have:*<br>☐ Justified the exclusion from the review of each potentially relevant study	☐ Yes<br>☐ Partial Yes<br>☐ **No**	Minor limitation.
8. Did the review authors describe the included studies in adequate detail?	Yes	
9. Did the review authors use a satisfactory technique for assessing the methodological limitations / risk of bias (RoB) in individual studies that were included in the review?	No	Major limitation.
10. Did the review authors report on the sources of funding for the studies included in the review?	No	Minor limitation.
11. If a synthesis was performed did the review authors use appropriate methods to combine the results of individual studies?	Yes	
12. If synthesis was performed, did the review authors assess the potential impact of the methodological limitations / RoB in individual studies on the results of the evidence synthesis?	No	Minor limitation.
13. Did the review authors account for methodological limitations / RoB in individual studies when interpreting/ discussing the results of the review?	Yes	

14. Did the review authors provide a satisfactory explanation for, and discussion of, any heterogeneity observed in the results of the review?<br><br>(*Omit for QES and for some reviews of survey data*)	No	Minor limitation.
15. If they performed quantitative synthesis did the review authors carry out an adequate investigation of publication bias (small study bias) and discuss its likely impact on the results of the review?<br><br>(*Omit for QES*)	Not applicable.	Not performed.
16. Did the review authors report any potential sources of conflict of interest, including any funding they received for conducting the review?	No	Minor limitation.

**Overall assessment**	**Important limitations.**
**Explanation of overall assessment**	**Two major limitations. Eight minor limitations.**

Criteria for assessing reviews (adapted from AMSTAR)

Review author, year: Newman et al., 2013.

Criterion	Assessment	Comments
1. Did the review have a clear research question and inclusion criteria?	Yes	
2. Did the report of the review contain an explicit statement that the review methods were established prior to the conduct of the review and did the report justify any significant deviations from the protocol?	No	Minor limitation.
3. Did the review authors explain their selection of the study designs for inclusion in the review?	No	Minor limitation.
4. Did the review authors use a comprehensive literature search strategy?<br><br>*For Partial Yes (all the following):*<br>☐ **searched** at least 2 databases (relevant to research question)<br>☐ **provided** key word and/or search strategy<br>☐ **justified** publication restrictions (e.g. language)<br><br>*For Yes, should also have (all the following):*<br>☐ searched the reference lists / bibliographies of included studies<br>☐ included/consulted content experts in the field<br>☐ where relevant, searched for grey literature<br>☐ conducted search within 24 months of completion of the review	☐ Yes<br>☐ **Partial** Yes<br>☐ No	Search strategy not provided in detail. No major publication restrictions. Minor limitation.

5. Did the review authors perform study selection in duplicate?	No	Minor limitation.
6. Did the review authors perform data extraction in duplicate?	No	Minor limitation.
7. Did the review authors provide a list of excluded studies and justify the exclusions?<br><br>*For Partial Yes:*<br>☐ provided a list of all potentially relevant studies that were read in full-text form but excluded from the review<br><br>*For Yes, must also have:*<br>☐ Justified the exclusion from the review of each potentially relevant study	☐ Yes<br>☐ Partial Yes<br>☐ **No**	Minor limitation.
8. Did the review authors describe the included studies in adequate detail?	Yes	
9. Did the review authors use a satisfactory technique for assessing the methodological limitations / risk of bias (RoB) in individual studies that were included in the review?	Yes	"We assessed risk of bias using items from the Effective Public Health Practice Project (EPHPP) 'Quality Assessment Tool for Quantitative Studies',28 which we modified for use with cross-sectional studies."
10. Did the review authors report on the sources of funding for the studies included in the review?	No	Minor limitation.
11. If a synthesis was performed did the review authors use appropriate methods to combine the results of individual studies?	Yes	
12. If synthesis was performed, did the review authors assess the potential impact of the methodological limitations / RoB in individual studies on the results of the evidence synthesis?	Yes	
13. Did the review authors account for methodological limitations / RoB in individual studies when interpreting/ discussing the results of the review?	Yes	

14. Did the review authors provide a satisfactory explanation for, and discussion of, any heterogeneity observed in the results of the review?<br><br>(*Omit for QES and for some reviews of survey data*)	Yes	
15. If they performed quantitative synthesis did the review authors carry out an adequate investigation of publication bias (small study bias) and discuss its likely impact on the results of the review?<br><br>(*Omit for QES*)	No	Minor limitation.
16. Did the review authors report any potential sources of conflict of interest, including any funding they received for conducting the review?	Yes	"This research was funded in part by grants from the Canadian Institutes of Health Research (funding reference number THA-118570) through the Canadian HIV Vaccine Initiative, and the Canada Research Chairs program (950-204522)."

**Overall assessment**	**Minor limitations.**
**Explanation of overall assessment**	**Eight minor limitations.**

Criteria for assessing reviews (adapted from AMSTAR)

Review author, year: Newman et al., 2018.

Criterion	Assessment	Comments
1. Did the review have a clear research question and inclusion criteria?	Yes	
2. Did the report of the review contain an explicit statement that the review methods were established prior to the conduct of the review and did the report justify any significant deviations from the protocol?	No	Searches in Prospero and BMC systematic reviews yielded no results. No relevant statement found. Minor limitation.
3. Did the review authors explain their selection of the study designs for inclusion in the review?	No	Minor limitation.
4. Did the review authors use a comprehensive literature search strategy?<br><br>*For Partial Yes (all the following):*<br>☐ **searched** at least 2 databases (relevant to research question)<br>☐ **provided** key word and/or search strategy<br>☐ **justified** publication restrictions (e.g. language)<br><br>*For Yes, should also have (all the following):*<br>☐ **searched** the reference lists / bibliographies of included studies<br>☐ included/consulted content experts in the field<br>☐ where relevant, searched for grey literature<br>☐ conducted search within 24 months of completion of the review	☐ Yes<br>☐ **Partial** Yes<br>☐ No	No publication limitations to justify. Minor limitation.

5. Did the review authors perform study selection in duplicate?	Yes	
6. Did the review authors perform data extraction in duplicate?	Yes	
7. Did the review authors provide a list of excluded studies and justify the exclusions?<br><br>*For Partial Yes:*<br>☐ provided a list of all potentially relevant studies that were read in full-text form but excluded from the review<br><br>*For Yes, must also have:*<br>☐ Justified the exclusion from the review of each potentially relevant study	☐ Yes<br>☐ Partial Yes<br>☐ **No**	No list of excluded studies provided. Overall reasons for exclusion provided, but not for each study. Minor limitation.
8. Did the review authors describe the included studies in adequate detail?	Yes	
9. Did the review authors use a satisfactory technique for assessing the methodological limitations / risk of bias (RoB) in individual studies that were included in the review?	Yes	
10. Did the review authors report on the sources of funding for the studies included in the review?	Yes	
11. If a synthesis was performed did the review authors use appropriate methods to combine the results of individual studies?	Yes	
12. If synthesis was performed, did the review authors assess the potential impact of the methodological limitations / RoB in individual studies on the results of the evidence synthesis?	Yes	
13. Did the review authors account for methodological limitations / RoB in individual studies when interpreting/ discussing the results of the review?	Yes	

14. Did the review authors provide a satisfactory explanation for, and discussion of, any heterogeneity observed in the results of the review?<br><br>(*Omit for QES and for some reviews of survey data*)	Yes	
15. If they performed quantitative synthesis did the review authors carry out an adequate investigation of publication bias (small study bias) and discuss its likely impact on the results of the review?<br><br>(*Omit for QES*)	Yes	
16. Did the review authors report any potential sources of conflict of interest, including any funding they received for conducting the review?	Yes	

**Overall assessment**	**Minor limitations.**
**Explanation of overall assessment**	**Four minor limitations.**

Criteria for assessing reviews (adapted from AMSTAR)

Review author, year: Niccolai et al., 2015.

Criterion	Assessment	Comments
1. Did the review have a clear research question and inclusion criteria?	Yes	
2. Did the report of the review contain an explicit statement that the review methods were established prior to the conduct of the review and did the report justify any significant deviations from the protocol?	No	Searches in Prospero and BMC Systematic Reviews yielded no results. No relevant statement found. Minor limitation.
3. Did the review authors explain their selection of the study designs for inclusion in the review?	No	Minor limitation.
4. Did the review authors use a comprehensive literature search strategy?<br><br>*For Partial Yes (all the following):*<br>☐ **searched** at least 2 databases (relevant to research question)<br>☐ **provided** key word and/or search strategy<br>☐ **justified** publication restrictions (e.g. language)<br><br>*For Yes, should also have (all the following):*<br>☐ **searched** the reference lists / bibliographies of included studies<br>☐ included/consulted content experts in the field<br>☐ where relevant, searched for grey literature<br>☐ conducted search within 24 months of completion of the review	☐ Yes<br>☐ **Partial** Yes<br>☐ No	No major publication restrictions to explain. Minor limitation

5. Did the review authors perform study selection in duplicate?	No	Not specified. Minor limitation.
6. Did the review authors perform data extraction in duplicate?	Yes	
7. Did the review authors provide a list of excluded studies and justify the exclusions?<br><br>*For Partial Yes:*<br>☐ provided a list of all potentially relevant studies that were read in full-text form but excluded from the review<br><br>*For Yes, must also have:*<br>☐ Justified the exclusion from the review of each potentially relevant study	☐ Yes<br>☐ Partial Yes<br>☐ **No**	Minor limitation.
8. Did the review authors describe the included studies in adequate detail?	Yes	
9. Did the review authors use a satisfactory technique for assessing the methodological limitations / risk of bias (RoB) in individual studies that were included in the review?	No	Major limitation.
10. Did the review authors report on the sources of funding for the studies included in the review?	No	Minor limitation.
11. If a synthesis was performed did the review authors use appropriate methods to combine the results of individual studies?	Yes	
12. If synthesis was performed, did the review authors assess the potential impact of the methodological limitations / RoB in individual studies on the results of the evidence synthesis?	No	Minor limitation.
13. Did the review authors account for methodological limitations / RoB in individual studies when interpreting/ discussing the results of the review?	No	Minor limitation.

14. Did the review authors provide a satisfactory explanation for, and discussion of, any heterogeneity observed in the results of the review?<br><br>(*Omit for QES and for some reviews of survey data*)	No	Formal meta-analysis was not performed because of heterogeneity across studies in outcomes measures. No heterogeneity in results explained. Minor limitation.
15. If they performed quantitative synthesis did the review authors carry out an adequate investigation of publication bias (small study bias) and discuss its likely impact on the results of the review?<br><br>(*Omit for QES*)	Not applicable.	Not performed.
16. Did the review authors report any potential sources of conflict of interest, including any funding they received for conducting the review?	Yes	"The funding source had no role in the design and conduct of the study; collection, management, analysis, and interpretation of the data; preparation, review, or approval of the manuscript; and the decision to submit the manuscript for publication. This work was supported by grant T32 AI007210 from the National Institutes of Health (Dr Hansen)."

**Overall assessment**	**Important limitations.**
**Explanation of overall assessment**	**One major limitation. Nine minor limitations.**

Criteria for assessing reviews (adapted from AMSTAR)

Review author, year: Paul et al., 2013.

Criterion	Assessment	Comments
1. Did the review have a clear research question and inclusion criteria?	Yes	
2. Did the report of the review contain an explicit statement that the review methods were established prior to the conduct of the review and did the report justify any significant deviations from the protocol?	No	Not found. Minor limitation.
3. Did the review authors explain their selection of the study designs for inclusion in the review?	Yes	No selection made.
4. Did the review authors use a comprehensive literature search strategy?<br><br>*For Partial Yes (all the following):*<br>☐ searched at least 2 databases (relevant to research question)<br>☐ **provided** key word and/or search strategy<br>☐ justified publication restrictions (e.g. language)<br><br>*For Yes, should also have (all the following):*<br>☐ searched the reference lists / bibliographies of included studies<br>☐ included/consulted content experts in the field<br>☐ where relevant, searched for grey literature<br>☐ conducted search within 24 months of completion of the review	☐ Yes<br>☐ Partial Yes<br>☐ **No**	Only one database searched. Publication restrictions not justified. Major limitation.

5. Did the review authors perform study selection in duplicate?	No	Not specified. Minor limitation.
6. Did the review authors perform data extraction in duplicate?	No	Not specified. Minor limitation.
7. Did the review authors provide a list of excluded studies and justify the exclusions?<br><br>*For Partial Yes:*<br>☐ provided a list of all potentially relevant studies that were read in full-text form but excluded from the review<br><br>*For Yes, must also have:*<br>☐ Justified the exclusion from the review of each potentially relevant study	☐ Yes<br>☐ Partial Yes<br>☐ **No**	Minor limitation.
8. Did the review authors describe the included studies in adequate detail?	Yes	
9. Did the review authors use a satisfactory technique for assessing the methodological limitations / risk of bias (RoB) in individual studies that were included in the review?	No	Major limitation.
10. Did the review authors report on the sources of funding for the studies included in the review?	No	Minor limitation.
11. If a synthesis was performed did the review authors use appropriate methods to combine the results of individual studies?	Yes	
12. If synthesis was performed, did the review authors assess the potential impact of the methodological limitations / RoB in individual studies on the results of the evidence synthesis?	No	Minor limitation.
13. Did the review authors account for methodological limitations / RoB in individual studies when interpreting/ discussing the results of the review?	No	Minor limitation.

14. Did the review authors provide a satisfactory explanation for, and discussion of, any heterogeneity observed in the results of the review?<br><br>(*Omit for QES and for some reviews of survey data*)	Yes	
15. If they performed quantitative synthesis did the review authors carry out an adequate investigation of publication bias (small study bias) and discuss its likely impact on the results of the review?<br><br>(*Omit for QES*)	Not applicable.	
16. Did the review authors report any potential sources of conflict of interest, including any funding they received for conducting the review?	No	Minor limitation.

**Overall assessment**	**Important limitations.**
**Explanation of overall assessment**	**Two major limitations. Eight minor limitations.**

Criteria for assessing reviews (adapted from AMSTAR)

Review author, year: Perlman, S. 2014.

Criterion	Assessment	Comments
1. Did the review have a clear research question and inclusion criteria?	Yes	
2. Did the report of the review contain an explicit statement that the review methods were established prior to the conduct of the review and did the report justify any significant deviations from the protocol?	No	Searches in Prospero and BMC Systematic Reviews yielded no results. No relevant statement found. Minor limitation.
3. Did the review authors explain their selection of the study designs for inclusion in the review?	Yes	No selection to explain.
4. Did the review authors use a comprehensive literature search strategy?<br><br>*For Partial Yes (all the following):*<br>☐ **searched** at least 2 databases (relevant to research question)<br>☐ **provided** key word and/or search strategy<br>☐ **justified** publication restrictions (e.g. language)<br><br>*For Yes, should also have (all the following):*<br>☐ **searched** the reference lists / bibliographies of included studies<br>☐ included/consulted content experts in the field<br>☐ where relevant, searched for grey literature<br>☐ conducted search within 24 months of completion of the review	☐ Yes<br>☐ **Partial** Yes<br>☐ No	Minor limitation.

5. Did the review authors perform study selection in duplicate?	No	Minor limitation.
6. Did the review authors perform data extraction in duplicate?	No	Minor limitation.
7. Did the review authors provide a list of excluded studies and justify the exclusions?<br><br>*For Partial Yes:*<br>☐ provided a list of all potentially relevant studies that were read in full-text form but excluded from the review<br><br>*For Yes, must also have:*<br>☐ Justified the exclusion from the review of each potentially relevant study	☐ Yes<br>☐ Partial Yes<br>☐ **No**	Minor limitation.
8. Did the review authors describe the included studies in adequate detail?	Yes	
9. Did the review authors use a satisfactory technique for assessing the methodological limitations / risk of bias (RoB) in individual studies that were included in the review?	No	Major limitation.
10. Did the review authors report on the sources of funding for the studies included in the review?	No	Minor limitation.
11. If a synthesis was performed did the review authors use appropriate methods to combine the results of individual studies?	Yes	
12. If synthesis was performed, did the review authors assess the potential impact of the methodological limitations / RoB in individual studies on the results of the evidence synthesis?	No	Minor limitation.
13. Did the review authors account for methodological limitations / RoB in individual studies when interpreting/ discussing the results of the review?	No	Minor limitation.

14. Did the review authors provide a satisfactory explanation for, and discussion of, any heterogeneity observed in the results of the review?<br><br>(*Omit for QES and for some reviews of survey data*)	No	Minor limitation.
15. If they performed quantitative synthesis did the review authors carry out an adequate investigation of publication bias (small study bias) and discuss its likely impact on the results of the review?<br><br>(*Omit for QES*)	Not applicable.	Not performed.
16. Did the review authors report any potential sources of conflict of interest, including any funding they received for conducting the review?	No	"Funding: The authors have no support or funding to report."

**Overall assessment**	**Important limitations.**
**Explanation of overall assessment**	**One major limitation. Nine minor limitations.**

Criteria for assessing reviews (adapted from AMSTAR)

Review author, year: Radisic et al., 2016.

Criterion	Assessment	Comments
1. Did the review have a clear research question and inclusion criteria?	Yes	
2. Did the report of the review contain an explicit statement that the review methods were established prior to the conduct of the review and did the report justify any significant deviations from the protocol?	No	Searches in Prospero and BMC Systematic Reviews yielded no results. No relevant statement found. Minor limitation.
3. Did the review authors explain their selection of the study designs for inclusion in the review?	Yes	No selection of study designs needing explanation.
4. Did the review authors use a comprehensive literature search strategy?<br><br>*For Partial Yes (all the following):*<br>☐ **searched** at least 2 databases (relevant to research question)<br>☐ **provided** key word and/or search strategy<br>☐ justified publication restrictions (e.g. language)<br><br>*For Yes, should also have (all the following):*<br>☐ searched the reference lists / bibliographies of included studies<br>☐ included/consulted content experts in the field<br>☐ where relevant, searched for grey literature<br>☐ conducted search within 24 months of completion of the review	☐ Yes<br>☐ Partial Yes<br>☐ **No**	Language restriction not justified. Minor limitation.

5. Did the review authors perform study selection in duplicate?	No	Minor limitation.
6. Did the review authors perform data extraction in duplicate?	No	Minor limitation.
7. Did the review authors provide a list of excluded studies and justify the exclusions?<br><br>*For Partial Yes:*<br>☐ provided a list of all potentially relevant studies that were read in full-text form but excluded from the review<br><br>*For Yes, must also have:*<br>☐ Justified the exclusion from the review of each potentially relevant study	☐ Yes<br>☐ Partial Yes<br>☐ **No**	Minor limitation.
8. Did the review authors describe the included studies in adequate detail?	Yes	
9. Did the review authors use a satisfactory technique for assessing the methodological limitations / risk of bias (RoB) in individual studies that were included in the review?	Yes	MMAT provided for individual studies.
10. Did the review authors report on the sources of funding for the studies included in the review?	No	Minor limitation.
11. If a synthesis was performed did the review authors use appropriate methods to combine the results of individual studies?	Yes	
12. If synthesis was performed, did the review authors assess the potential impact of the methodological limitations / RoB in individual studies on the results of the evidence synthesis?	Yes	
13. Did the review authors account for methodological limitations / RoB in individual studies when interpreting/ discussing the results of the review?	Yes	

14. Did the review authors provide a satisfactory explanation for, and discussion of, any heterogeneity observed in the results of the review?<br><br>(*Omit for QES and for some reviews of survey data*)	Yes	
15. If they performed quantitative synthesis did the review authors carry out an adequate investigation of publication bias (small study bias) and discuss its likely impact on the results of the review?<br><br>(*Omit for QES*)	Not applicable.	
16. Did the review authors report any potential sources of conflict of interest, including any funding they received for conducting the review?	No	

**Overall assessment**	**Minor limitations.**
**Explanation of overall assessment**	**Six minor limitations.**

Criteria for assessing reviews (adapted from AMSTAR)

Review author, year: Rambout, L. 2013.

Criterion	Assessment	Comments
1. Did the review have a clear research question and inclusion criteria?	Yes	
2. Did the report of the review contain an explicit statement that the review methods were established prior to the conduct of the review and did the report justify any significant deviations from the protocol?	No	Searches in Prospero and BMC Systematic Reviews yielded no results. No relevant statement found. Minor limitation.
3. Did the review authors explain their selection of the study designs for inclusion in the review?	Yes	No selection of study designs needing explanation.
4. Did the review authors use a comprehensive literature search strategy?<br><br>*For Partial Yes (all the following):*<br>☐ **searched** at least 2 databases (relevant to research question)<br>☐ **provided** key word and/or search strategy<br>☐ **justified** publication restrictions (e.g. language)<br><br>*For Yes, should also have (all the following):*<br>☐ **searched** the reference lists / bibliographies of included studies<br>☐ included/consulted content experts in the field<br>☐ where relevant, searched for grey literature<br>☐ conducted search within 24 months of completion of the review	☐ Yes<br>☐ **Partial** Yes<br>☐ No	Minor limitation.

5. Did the review authors perform study selection in duplicate?	No	Minor limitation.
6. Did the review authors perform data extraction in duplicate?	Yes	
7. Did the review authors provide a list of excluded studies and justify the exclusions?<br><br>*For Partial Yes:*<br>☐ provided a list of all potentially relevant studies that were read in full-text form but excluded from the review<br><br>*For Yes, must also have:*<br>☐ Justified the exclusion from the review of each potentially relevant study	☐ Yes<br>☐ Partial Yes<br>☐ **No**	Minor limitation.
8. Did the review authors describe the included studies in adequate detail?	Yes	
9. Did the review authors use a satisfactory technique for assessing the methodological limitations / risk of bias (RoB) in individual studies that were included in the review?	Yes	
10. Did the review authors report on the sources of funding for the studies included in the review?	Yes	
11. If a synthesis was performed did the review authors use appropriate methods to combine the results of individual studies?	Yes	
12. If synthesis was performed, did the review authors assess the potential impact of the methodological limitations / RoB in individual studies on the results of the evidence synthesis?	Yes	
13. Did the review authors account for methodological limitations / RoB in individual studies when interpreting/ discussing the results of the review?	Yes	

14. Did the review authors provide a satisfactory explanation for, and discussion of, any heterogeneity observed in the results of the review?<br><br>(*Omit for QES and for some reviews of survey data*)	No	Any heterogeneity in results was not explained. Minor limitation.
15. If they performed quantitative synthesis did the review authors carry out an adequate investigation of publication bias (small study bias) and discuss its likely impact on the results of the review?<br><br>(*Omit for QES*)	Not applicable.	Heterogeneity among studies precluded meta-analysis.
16. Did the review authors report any potential sources of conflict of interest, including any funding they received for conducting the review?	Yes	"All authors have completed the Conflict of Interest Policy available on request from the corresponding author and declare that: (1) LR, MT, LH, & ACT did not receive any financial support for the submitted work; (2) LR, MT, & LH have no relationships with any companies that might have an interest in the submitted work in the previous 3 years, ACT was a consultant for GlaxoSmithKline Canada vaccine products, including their human papillomavirus vaccine from December 2003 until September 2011; (3) their spouses, partners, or children have no financial relationships that may be relevant to the submitted work; and (4) LR, MT, LH, & ACT have no non-financial interests that may be relevant to the submitted work."

**Overall assessment**	**Minor limitations.**
**Explanation of overall assessment**	**Five minor limitations.**

Criteria for assessing reviews (adapted from AMSTAR)

Review author, year: Rosen et al., 2017.

Criterion	Assessment	Comments
1. Did the review have a clear research question and inclusion criteria?	Yes	
2. Did the report of the review contain an explicit statement that the review methods were established prior to the conduct of the review and did the report justify any significant deviations from the protocol?	No	Searches in Prospero and BMC Systematic Reviews yielded no results. No relevant statement found. Minor limitation.
3. Did the review authors explain their selection of the study designs for inclusion in the review?	Yes	No selection in need of an explanation.
4. Did the review authors use a comprehensive literature search strategy?<br><br>*For Partial Yes (all the following):*<br>☐ **searched** at least 2 databases (relevant to research question)<br>☐ **provided** key word and/or search strategy<br>☐ **justified** publication restrictions (e.g. language)<br><br>*For Yes, should also have (all the following):*<br>☐ searched the reference lists / bibliographies of included studies<br>☐ included/consulted content experts in the field<br>☐ where relevant, searched for grey literature<br>☐ conducted search within 24 months of completion of the review	☐ Yes<br>☐ **Partial** Yes<br>☐ No	No publication restrictions in need of an explanation. Minor limitation.

5. Did the review authors perform study selection in duplicate?	No	Not reported. Minor limitation.
6. Did the review authors perform data extraction in duplicate?	No	Minor limitation.
7. Did the review authors provide a list of excluded studies and justify the exclusions?<br><br>*For Partial Yes:*<br>☐ provided a list of all potentially relevant studies that were read in full-text form but excluded from the review<br><br>*For Yes, must also have:*<br>☐ Justified the exclusion from the review of each potentially relevant study	☐ Yes<br>☐ Partial Yes<br>☐ **No**	Minor limitation.
8. Did the review authors describe the included studies in adequate detail?	Yes	
9. Did the review authors use a satisfactory technique for assessing the methodological limitations / risk of bias (RoB) in individual studies that were included in the review?	No	Major limitation.
10. Did the review authors report on the sources of funding for the studies included in the review?	No	Minor limitation.
11. If a synthesis was performed did the review authors use appropriate methods to combine the results of individual studies?	Yes	
12. If synthesis was performed, did the review authors assess the potential impact of the methodological limitations / RoB in individual studies on the results of the evidence synthesis?	No	Minor limitation.
13. Did the review authors account for methodological limitations / RoB in individual studies when interpreting/ discussing the results of the review?	Yes	

14. Did the review authors provide a satisfactory explanation for, and discussion of, any heterogeneity observed in the results of the review?<br><br>(*Omit for QES and for some reviews of survey data*)	No	Minor limitation.
15. If they performed quantitative synthesis did the review authors carry out an adequate investigation of publication bias (small study bias) and discuss its likely impact on the results of the review?<br><br>(*Omit for QES*)	No	Minor limitation.
16. Did the review authors report any potential sources of conflict of interest, including any funding they received for conducting the review?	Yes	

**Overall assessment**	**Important limitations.**
**Explanation of overall assessment**	**One major limitation. Nine minor limitations.**

Criteria for assessing reviews (adapted from AMSTAR)

Review author, year: Ryan et al., 2018.

Criterion	Assessment	Comments
1. Did the review have a clear research question and inclusion criteria?	Yes	
2. Did the report of the review contain an explicit statement that the review methods were established prior to the conduct of the review and did the report justify any significant deviations from the protocol?	No	Searches in Prospero and BMC systematic reviews yielded no results. No relevant statement found. Minor limitation.
3. Did the review authors explain their selection of the study designs for inclusion in the review?	No	Minor limitation.
4. Did the review authors use a comprehensive literature search strategy?<br><br>*For Partial Yes (all the following):*<br>☐ **searched** at least 2 databases (relevant to research question)<br>☐ **provided** key word and/or search strategy<br>☐ justified publication restrictions (e.g. language)<br><br>*For Yes, should also have (all the following):*<br>☐ searched the reference lists / bibliographies of included studies<br>☐ included/consulted content experts in the field<br>☐ where relevant, searched for grey literature<br>☐ conducted search within 24 months of completion of the review	☐ Yes<br>☐ **Partial** Yes<br>☐ No	No publication restrictions in need of justification. Minor limitation.

5. Did the review authors perform study selection in duplicate?	No	
6. Did the review authors perform data extraction in duplicate?	No	
7. Did the review authors provide a list of excluded studies and justify the exclusions?<br><br>*For Partial Yes:*<br>☐ provided a list of all potentially relevant studies that were read in full-text form but excluded from the review<br><br>*For Yes, must also have:*<br>☐ Justified the exclusion from the review of each potentially relevant study	☐ Yes<br>☐ Partial Yes<br>☐ **No**	No reported reasoning behind or list of exclusion of studies. Minor limitation.
8. Did the review authors describe the included studies in adequate detail?	Yes	
9. Did the review authors use a satisfactory technique for assessing the methodological limitations / risk of bias (RoB) in individual studies that were included in the review?	No	Major limitation.
10. Did the review authors report on the sources of funding for the studies included in the review?	No	Minor limitation.
11. If a synthesis was performed did the review authors use appropriate methods to combine the results of individual studies?	Yes	
12. If synthesis was performed, did the review authors assess the potential impact of the methodological limitations / RoB in individual studies on the results of the evidence synthesis?	No	Minor limitation.
13. Did the review authors account for methodological limitations / RoB in individual studies when interpreting/ discussing the results of the review?	No	Minor limitation.

14. Did the review authors provide a satisfactory explanation for, and discussion of, any heterogeneity observed in the results of the review?<br><br>(*Omit for QES and for some reviews of survey data*)	Not applicable	
15. If they performed quantitative synthesis did the review authors carry out an adequate investigation of publication bias (small study bias) and discuss its likely impact on the results of the review?<br><br>(*Omit for QES*)	Not applicable	
16. Did the review authors report any potential sources of conflict of interest, including any funding they received for conducting the review?	No	Minor limitation.

**Overall assessment**	**Important limitations.**
**Explanation of overall assessment**	**One major limitations. Eight minor limitations.**

Criteria for assessing reviews (adapted from AMSTAR)

Review author, year: Small et al., 2014.

Criterion	Assessment	Comments
1. Did the review have a clear research question and inclusion criteria?	Yes	
2. Did the report of the review contain an explicit statement that the review methods were established prior to the conduct of the review and did the report justify any significant deviations from the protocol?	No	Minor limitation.
3. Did the review authors explain their selection of the study designs for inclusion in the review?	Yes	No selection of study designs made.
4. Did the review authors use a comprehensive literature search strategy?<br><br>*For Partial Yes (all the following):*<br>☐ **searched** at least 2 databases (relevant to research question)<br>☐ **provided** key word and/or search strategy<br>☐ **justified** publication restrictions (e.g. language)<br><br>*For Yes, should also have (all the following):*<br>☐ **searched** the reference lists / bibliographies of included studies<br>☐ included/consulted content experts in the field<br>☐ where relevant, searched for grey literature<br>☐ conducted search within 24 months of completion of the review	☐ Yes<br>☐ **Partial** Yes<br>☐ No	Minor limitation.

5. Did the review authors perform study selection in duplicate?	No	Not specified. Minor limitation.
6. Did the review authors perform data extraction in duplicate?	No	Not specified. Minor limitation.
7. Did the review authors provide a list of excluded studies and justify the exclusions?<br><br>*For Partial Yes:*<br>☐ provided a list of all potentially relevant studies that were read in full-text form but excluded from the review<br><br>*For Yes, must also have:*<br>☐ Justified the exclusion from the review of each potentially relevant study	☐ Yes<br>☐ Partial Yes<br>☐ **No**	Minor limitation.
8. Did the review authors describe the included studies in adequate detail?	Yes	
9. Did the review authors use a satisfactory technique for assessing the methodological limitations / risk of bias (RoB) in individual studies that were included in the review?	No	Major limitation.
10. Did the review authors report on the sources of funding for the studies included in the review?	No	Minor limitation.
11. If a synthesis was performed did the review authors use appropriate methods to combine the results of individual studies?	Yes	
12. If synthesis was performed, did the review authors assess the potential impact of the methodological limitations / RoB in individual studies on the results of the evidence synthesis?	No	Minor limitation.
13. Did the review authors account for methodological limitations / RoB in individual studies when interpreting/ discussing the results of the review?	No	Minor limitation.

14. Did the review authors provide a satisfactory explanation for, and discussion of, any heterogeneity observed in the results of the review?<br><br>(*Omit for QES and for some reviews of survey data*)	No	Minor limitation.
15. If they performed quantitative synthesis did the review authors carry out an adequate investigation of publication bias (small study bias) and discuss its likely impact on the results of the review?<br><br>(*Omit for QES*)	Not applicable	Not performed.
16. Did the review authors report any potential sources of conflict of interest, including any funding they received for conducting the review?	Yes	"Investigator support provided by the Health Promotion/Risk Reduction Interventions with Vulnerable Pop- ulations Training Grant (5 T32 NR007073-18)."

**Overall assessment**	**Important limitations.**
**Explanation of overall assessment**	**One major limitation. Nine minor limitations.**

Criteria for assessing reviews (adapted from AMSTAR)

Review author, year: Smulian et al., 2016.

Criterion	Assessment	Comments
1. Did the review have a clear research question and inclusion criteria?	Yes	
2. Did the report of the review contain an explicit statement that the review methods were established prior to the conduct of the review and did the report justify any significant deviations from the protocol?	No	Searches in Prospero and BMC Systematic Reviews yielded no results. No relevant statement found. Minor limitation.
3. Did the review authors explain their selection of the study designs for inclusion in the review?	No	Minor limitation.
4. Did the review authors use a comprehensive literature search strategy?<br><br>*For Partial Yes (all the following):*<br>☐ **searched** at least 2 databases (relevant to research question)<br>☐ provided key word and/or search strategy<br>☐ justified publication restrictions (e.g. language)<br><br>*For Yes, should also have (all the following):*<br>☐ searched the reference lists / bibliographies of included studies<br>☐ included/consulted content experts in the field<br>☐ where relevant, searched for grey literature<br>☐ conducted search within 24 months of completion of the review	☐ Yes<br>☐ Partial Yes<br>☐ **No**	No key word or search strategy provided. Major limitation.

5. Did the review authors perform study selection in duplicate?	No	Not specified. Minor limitation.
6. Did the review authors perform data extraction in duplicate?	No	Not specified. Minor limitation.
7. Did the review authors provide a list of excluded studies and justify the exclusions?<br><br>*For Partial Yes:*<br>☐ provided a list of all potentially relevant studies that were read in full-text form but excluded from the review<br><br>*For Yes, must also have:*<br>☐ Justified the exclusion from the review of each potentially relevant study	☐ Yes<br>☐ Partial Yes<br>☐ **No**	Reasons for exclusion reported by number, but not for individual studies. Minor limitation.
8. Did the review authors describe the included studies in adequate detail?	Yes	
9. Did the review authors use a satisfactory technique for assessing the methodological limitations / risk of bias (RoB) in individual studies that were included in the review?	No	Major limitation.
10. Did the review authors report on the sources of funding for the studies included in the review?	No	Minor limitation.
11. If a synthesis was performed did the review authors use appropriate methods to combine the results of individual studies?	Yes	
12. If synthesis was performed, did the review authors assess the potential impact of the methodological limitations / RoB in individual studies on the results of the evidence synthesis?	No	Minor limitation.
13. Did the review authors account for methodological limitations / RoB in individual studies when interpreting/ discussing the results of the review?	No	Minor limitation.

14. Did the review authors provide a satisfactory explanation for, and discussion of, any heterogeneity observed in the results of the review?<br><br>(*Omit for QES and for some reviews of survey data*)	No	Minor limitation.
15. If they performed quantitative synthesis did the review authors carry out an adequate investigation of publication bias (small study bias) and discuss its likely impact on the results of the review?<br><br>(*Omit for QES*)	No	Minor limitation.
16. Did the review authors report any potential sources of conflict of interest, including any funding they received for conducting the review?	Yes	"This publication was supported by Cooperative Agreement Number 3U36OE000002 from the Centers for Disease Control and Prevention and the Association of Schools and Programs of Public Health. "<br>"This publication was also supported by 2013 Prevention and Public Health Funds, Immunization Program Technical and Analytical Assistance in Support of HPV Vaccination, Contract #200-2009-28537 Task Order-091."

**Overall assessment**	**Important limitations.**
**Explanation of overall assessment**	**Two major limitations. Ten minor limitations.**

Criteria for assessing reviews (adapted from AMSTAR)

Review author, year: Walling et al., 2016.

Criterion	Assessment	Comments
1. Did the review have a clear research question and inclusion criteria?	Yes	
2. Did the report of the review contain an explicit statement that the review methods were established prior to the conduct of the review and did the report justify any significant deviations from the protocol?	No	Searches in Prospero and BMC Systematic Reviews yielded no results. No relevant statement found. Minor limitation.
3. Did the review authors explain their selection of the study designs for inclusion in the review?	Yes	Set to “any”.
4. Did the review authors use a comprehensive literature search strategy?<br><br>*For Partial Yes (all the following):*<br>☐ **searched** at least 2 databases (relevant to research question)<br>☐ **provided** key word and/or search strategy<br>☐ justified publication restrictions (e.g. language)<br><br>*For Yes, should also have (all the following):*<br>☐ searched the reference lists / bibliographies of included studies<br>☐ included/consulted content experts in the field<br>☐ where relevant, searched for grey literature<br>☐ conducted search within 24 months of completion of the review	☐ Yes<br>☐ Partial Yes<br>☐ **No**	Time and language restriction not justified. Minor limitation.

5. Did the review authors perform study selection in duplicate?	No	Minor limitation.
6. Did the review authors perform data extraction in duplicate?	Yes	
7. Did the review authors provide a list of excluded studies and justify the exclusions?<br><br>*For Partial Yes:*<br>☐ provided a list of all potentially relevant studies that were read in full-text form but excluded from the review<br><br>*For Yes, must also have:*<br>☐ Justified the exclusion from the review of each potentially relevant study	☐ Yes<br>☐ Partial Yes<br>☐ **No**	Reasons for exclusion reported by number, but not for individual studies. Minor limitation.
8. Did the review authors describe the included studies in adequate detail?	Yes	
9. Did the review authors use a satisfactory technique for assessing the methodological limitations / risk of bias (RoB) in individual studies that were included in the review?	Yes	
10. Did the review authors report on the sources of funding for the studies included in the review?	No	Minor limitation.
11. If a synthesis was performed did the review authors use appropriate methods to combine the results of individual studies?	No	Major limitation
12. If synthesis was performed, did the review authors assess the potential impact of the methodological limitations / RoB in individual studies on the results of the evidence synthesis?	No	Minor limitation.
13. Did the review authors account for methodological limitations / RoB in individual studies when interpreting/ discussing the results of the review?	Yes	

14. Did the review authors provide a satisfactory explanation for, and discussion of, any heterogeneity observed in the results of the review?<br><br>(*Omit for QES and for some reviews of survey data*)	No	Minor limitation.
15. If they performed quantitative synthesis did the review authors carry out an adequate investigation of publication bias (small study bias) and discuss its likely impact on the results of the review?<br><br>(*Omit for QES*)	Not applicable.	Not performed.
16. Did the review authors report any potential sources of conflict of interest, including any funding they received for conducting the review?	Yes	"No external funding."

**Overall assessment**	**Important limitations.**
**Explanation of overall assessment**	**One major limitation. Seven minor limitations.**

Criteria for assessing reviews (adapted from AMSTAR)

Review author, year: Wigle et al., 2013.

Criterion	Assessment	Comments
1. Did the review have a clear research question and inclusion criteria?	Yes	
2. Did the report of the review contain an explicit statement that the review methods were established prior to the conduct of the review and did the report justify any significant deviations from the protocol?	No	Searches in Prospero and BMC Systematic Reviews yielded no results. No relevant statement found. Minor limitation.
3. Did the review authors explain their selection of the study designs for inclusion in the review?	Yes	No selection made.
4. Did the review authors use a comprehensive literature search strategy?<br><br>*For Partial Yes (all the following):*<br>☐ **searched** at least 2 databases (relevant to research question)<br>☐ **provided** key word and/or search strategy<br>☐ **justified** publication restrictions (e.g. language)<br><br>*For Yes, should also have (all the following):*<br>☐ searched the reference lists / bibliographies of included studies<br>☐ included/consulted content experts in the field<br>☐ where relevant, searched for grey literature<br>☐ conducted search within 24 months of completion of the review	☐ Yes<br>☐ **Partial** Yes<br>☐ No	Language restriction not justified. Minor limitation.

5. Did the review authors perform study selection in duplicate?	No	Minor limitation.
6. Did the review authors perform data extraction in duplicate?	No	Minor limitation.
7. Did the review authors provide a list of excluded studies and justify the exclusions?<br><br>*For Partial Yes:*<br>☐ provided a list of all potentially relevant studies that were read in full-text form but excluded from the review<br><br>*For Yes, must also have:*<br>☐ Justified the exclusion from the review of each potentially relevant study	☐ Yes<br>☐ Partial Yes<br>☐ **No**	Minor limitation.
8. Did the review authors describe the included studies in adequate detail?	No	No information about study designs, populations or health settings found. Minor limitation.
9. Did the review authors use a satisfactory technique for assessing the methodological limitations / risk of bias (RoB) in individual studies that were included in the review?	No	Major limitation.
10. Did the review authors report on the sources of funding for the studies included in the review?	No	Minor limitation.
11. If a synthesis was performed did the review authors use appropriate methods to combine the results of individual studies?	Yes	
12. If synthesis was performed, did the review authors assess the potential impact of the methodological limitations / RoB in individual studies on the results of the evidence synthesis?	No	Minor limitation.
13. Did the review authors account for methodological limitations / RoB in individual studies when interpreting/ discussing the results of the review?	No	Minor limitation.

14. Did the review authors provide a satisfactory explanation for, and discussion of, any heterogeneity observed in the results of the review?<br><br>(*Omit for QES and for some reviews of survey data*)	No	Minor limitation.
15. If they performed quantitative synthesis did the review authors carry out an adequate investigation of publication bias (small study bias) and discuss its likely impact on the results of the review?<br><br>(*Omit for QES*)	Not applicable.	
16. Did the review authors report any potential sources of conflict of interest, including any funding they received for conducting the review?	Yes	"DWJ [key informant] has received research funding from GSK Biologicals SA for a phase IIIb clinical trial of the bivalent HPV vaccine and has received a donation of the quadrivalent vaccine from Axios healthcare Development through the GARDASIL Access Program.."

**Overall assessment**	**Important limitations.**
**Explanation of overall assessment**	**One major limitation. Ten minor limitations.**

Criteria for assessing reviews (adapted from AMSTAR)

Review author, year: Young, A. 2010.

Criterion	Assessment	Comments
1. Did the review have a clear research question and inclusion criteria?	Yes	
2. Did the report of the review contain an explicit statement that the review methods were established prior to the conduct of the review and did the report justify any significant deviations from the protocol?	No	Searches in Prospero and BMC Systematic Reviews yielded no results. No relevant statement found. Minor limitation.
3. Did the review authors explain their selection of the study designs for inclusion in the review?	Yes	No selection made.
4. Did the review authors use a comprehensive literature search strategy?<br><br>*For Partial Yes (all the following):*<br>☐ **searched** at least 2 databases (relevant to research question)<br>☐ **provided** key word and/or search strategy<br>☐ justified publication restrictions (e.g. language)<br><br>*For Yes, should also have (all the following):*<br>☐ searched the reference lists / bibliographies of included studies<br>☐ included/consulted content experts in the field<br>☐ where relevant, searched for grey literature<br>☐ conducted search within 24 months of completion of the review	☐ Yes<br>☐ Partial Yes<br>☐ **No**	Time and language restrictions not justified. Minor limitation.

5. Did the review authors perform study selection in duplicate?	No	Not specified. Minor limitation.
6. Did the review authors perform data extraction in duplicate?	No	Not specified. Minor limitation.
7. Did the review authors provide a list of excluded studies and justify the exclusions?<br><br>*For Partial Yes:*<br>☐ provided a list of all potentially relevant studies that were read in full-text form but excluded from the review<br><br>*For Yes, must also have:*<br>☐ Justified the exclusion from the review of each potentially relevant study	☐ **Yes**<br>☐ Partial Yes<br>☐ No	
8. Did the review authors describe the included studies in adequate detail?	Yes	
9. Did the review authors use a satisfactory technique for assessing the methodological limitations / risk of bias (RoB) in individual studies that were included in the review?	No	Major limitation.
10. Did the review authors report on the sources of funding for the studies included in the review?	No	Minor limitation.
11. If a synthesis was performed did the review authors use appropriate methods to combine the results of individual studies?	Yes	
12. If synthesis was performed, did the review authors assess the potential impact of the methodological limitations / RoB in individual studies on the results of the evidence synthesis?	No	Minor limitation.
13. Did the review authors account for methodological limitations / RoB in individual studies when interpreting/ discussing the results of the review?	Yes	

14. Did the review authors provide a satisfactory explanation for, and discussion of, any heterogeneity observed in the results of the review?<br><br>(*Omit for QES and for some reviews of survey data*)	No	Minor limitation.
15. If they performed quantitative synthesis did the review authors carry out an adequate investigation of publication bias (small study bias) and discuss its likely impact on the results of the review?<br><br>(*Omit for QES*)	Not applicable.	Not performed.
16. Did the review authors report any potential sources of conflict of interest, including any funding they received for conducting the review?	No	Minor limitation.

**Overall assessment**	**Important limitations.**
**Explanation of overall assessment**	**One major limitation. Eight minor limitations.**
