# Supplementary material for: Communication around HPV vaccination for adolescents in low- and middle-income countries: a systematic scoping overview of systematic reviews
Source: Syst Rev. 2019 Aug 1;8:190. doi: 10.1186/s13643-019-1100-y (PMC6670236; doi:10.1186/s13643-019-1100-y)
Supplement: Supplementary file 11 — Plain language summary of the overview. (PDF 37 kb) [file 13643_2019_1100_MOESM11_ESM.pdf]

Additional file 11: Plain language summary of the overview

Communicating with adolescents and others about HPV vaccination, with a specific focus on low- and middle-income countries: a systematic scoping overview of reviews

What is the aim of this overview?

Our aim was to give a broad overview of the available research on communicating with adolescents and others about the human papillomavirus (HPV) vaccine. This was done so that researchers can see where new or updated reviews are needed on the topic. We had a specific focus on low- and middle-income-countries. We collected relevant systematic reviews of research studies and briefly described what they looked at.

Key messages

Most reviews that looked at communication about HPV vaccination for adolescents had problems in the way they were designed or carried out. Also, most of them did not find studies from low- and middle-income countries. This could point to a need for more research in these countries.

What was studied in the overview?

HPV infection can cause changes in the cells of the cervix which can later develop into cancer ("cervical cancer") if not treated. Most people diagnosed with this particular type of cancer live in low- and middle-income countries. The HPV vaccine can help prevent this type of cancer, but some people are skeptical about receiving the vaccine. Communication strategies may be able to change how people think about the vaccine and increase the number of people who are willing to be vaccinated.

We looked for research about adolescents or other people involved in their HPV vaccination, such as parents, communities, health care providers, health service managers and policy makers. The research had to address communication strategies in some way or another, such as by describing these strategies, measuring how effective they are, or finding out what people think of them.

What are the main results of the systematic overview?

We found twelve relevant reviews. Nine reviews searched for studies from low- and middle-income countries, but most only found a small number of studies from these countries. Four reviews described communication strategies or looked at how effective they were. These

included strategies to inform or educate people about HPV and HPV vaccines, such as videos and fact sheets; and strategies to remind people about vaccination appointments, such as text message reminders. Eight reviews found factors tied to communication that might influence how many people get vaccinated. These factors included whether the vaccine was recommended by a doctor and how much people already knew about the vaccine.

How up-to-date is this overview?

The overview authors searched for systematic reviews that had been published up to May 2018.
